# Novel Lanthanide (III) Complexes Derived from an Imidazole–Biphenyl–Carboxylate Ligand: Synthesis, Structure and Luminescence Properties

**DOI:** 10.3390/molecules26226942

**Published:** 2021-11-17

**Authors:** Monica-Cornelia Sardaru, Narcisa Laura Marangoci, Sergiu Shova, Dana Bejan

**Affiliations:** 1 Centre of Advanced Research in Bionanoconjugates and Biopolymers, “Petru Poni” Institute of Macromolecular Chemistry, Romanian Academy, Gr. Ghica Voda Alley, 700487 Iasi, Romania; sardaru.monica@icmpp.ro (M.-C.S.); nmarangoci@icmpp.ro (N.L.M.); 2 Department of Inorganic Polymers, “Petru Poni” Institute of Macromolecular Chemistry, Romanian Academy, Gr. Ghica Voda Alley, 700487 Iasi, Romania; shova@icmpp.ro

**Keywords:** lanthanide-coordination complex, solvothermal synthesis, crystal structure, luminescence

## Abstract

A series of neutral mononuclear lanthanide complexes **[Ln(HL)_2_(NO_3_)_3_]** (Ln = La, Ce, Nd, Eu, Gd, Dy, Ho) with rigid bidentate ligand, HL (4′-(1*H*-imidazol-1-yl)biphenyl-4-carboxylic acid) were synthesized under solvothermal conditions. The coordination compounds have been characterized by infrared spectroscopy, thermogravimetry, powder X-ray diffraction and elemental analysis. According to X-ray diffraction, all the complexes are a series of isostructural compounds crystallized in the *P2/n* monoclinic space group. Additionally, solid-state luminescence measurements of all complexes show that **[Eu(HL)_2_(NO_3_)_3_]** complex displays the characteristic emission peaks of Eu(III) ion at 593, 597, 615, and 651 nm.

## 1. Introduction

Over the last decades, metal-organic complexes formed by rare earth metals and multifunctional bridging ligands became of special interest to the scientific community because of their applications in various analytical, biological, and clinical fields [1,2,3,4]. They are widely used in catalytic processes [5,6,7], gas storage and separation [6,8,9], magnetic [10,11,12,13,14], chemical sensing [15,16], medical imaging and so on [17,18,19,20].

The coordination of lanthanides with various O and N donor ligands is extremely diverse and develops a large variety of materials as coordination polymers (CPs) [21,22,23], porous coordination polymers (PCPs) [24,25,26,27,28,29], and metal-organic framework coordination polymers (MOFs) [30,31,32,33,34,35,36,37]. Due to their architectures and by default of their physicochemical properties (higher thermodynamic stability, chemical stability and adjustable chemical functionalities, tunable pore surface, low density, magnetism, fluorescence, etc.), the rational design and synthesis of these materials is still an open topic for many researchers [38,39,40].

Lanthanides complexes, especially Eu(III) and Tb(III) complexes, are studied due to their luminescence properties [41,42,43], such as the strong emission bands of f-f transitions, broad range lifetimes, and high quantum yields that make them fluorescence materials with applications in medical-diagnostics [44,45]. Wu’s group demonstrated that the Eu-MOF, based on Eu and Gd, are promising biomarkers for a carcinoid tumor where as π-conjugated organic ligands are used carboxylates ligands (i.e., H_4_BTEC = 1,2,4,5-benzenetetracarboxylic acid and H_2_TDA = thiazolidine 2,4-dicaboxylic acid) [46]. Furthermore, due to their structural tunability of CPs and MOFs, their use as biomarkers in the diagnosis and/or detection of cancer disease has expanded significantly during the last decade [47].

During past years, our group has been involved in the research of synthesis and characterization of metal complexes with different ligands, and further exploring of their applications. Our attention was focused on aromatic ligands containing both -COO and n-donor groups, which show strong affinities to lanthanide metal.

It is well known that coordination numbers of lanthanides are determined by the size of the ligand or/and by the number of donor atoms that may pack around the metal. In the case of one bulky and rigid ligand, the coordination numbers of metal are strongly dependent on the interactions between distant groups and thus decide how many ligands may bind to the lanthanide ion. To our knowledge, lanthanide complexes with imidazole–biphenyl–carboxylate ligand are not reported yet. Therefore, in this work, we chose (4′-(1*H*-imidazol-1-yl)biphenyl-4-carboxylic acid) HL as ligand to construct a series of lanthanide complexes **[Ln(HL)_2_(NO_3_)_3_]** [Ln = La (1), Ce (2), Nd (3), Eu (4), Gd (5), Dy (6), Ho (7)] and these were characterized by elemental analysis, Fourier transform infrared spectroscopy (FT-IR), thermogravimetric analysis (TGA), single crystal X-ray diffraction and powder X-ray diffraction (PXRD) analysis. Also, the luminescence properties of complexes were investigated.

## 2. Results and Discussion

### 2.1. Synthesis and Characterization of [Ln(HL)_2_(NO_3_)_3_] Complexes

**Scheme 1:**
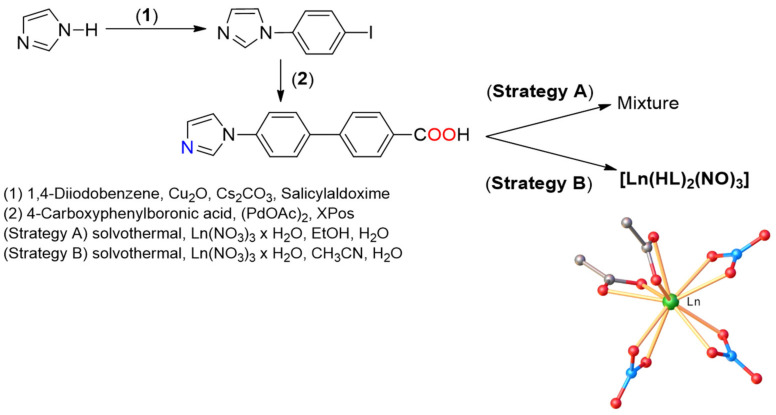
Strategy for the preparation of **[Ln(HL)_2_(NO_3_)_3_]**.

The present work deals with the multi-step synthesis of the rigid imidazole–biphenyl–carboxylate ligand **HL** [48]. The synthesis of **HL** involved the Ullmann coupling of 1,4-diiodobenzene with imidazole, followed by the Suzuki coupling of the reaction product with 4-carboxyphenylboronic acid as presented in Scheme 1. The mononuclear Ln^3+^ complexes were obtained by mixing hydrated lanthanide nitrate and the bidentate ligand, at the ratio 3:1 (metal salt/ligand) in different solvents. Several approaches for the complexation of ligand **HL** were explored (Scheme 1). First, the complexation reactions were attempted in an ethanol-water mixture at 80 °C for two to five days. Under these experimental conditions (see Strategy A), a mixture of product, starting material, and also unidentified complexes was obtained, as shown in the PXRD patterns in Figure S1 (Supplementary Materials). When the synthesized complexes were performed in solvents as acetonitrile and water (see Strategy B), at 120 °C (on the programmable oven), for two days, the products were realized in good yield and high purity. The conversion and the purity of the products were monitored by PXRD, as presented in Figure S2 (Supplementary Materials).

### 2.2. Spectroscopic Details

**Figure 1:**
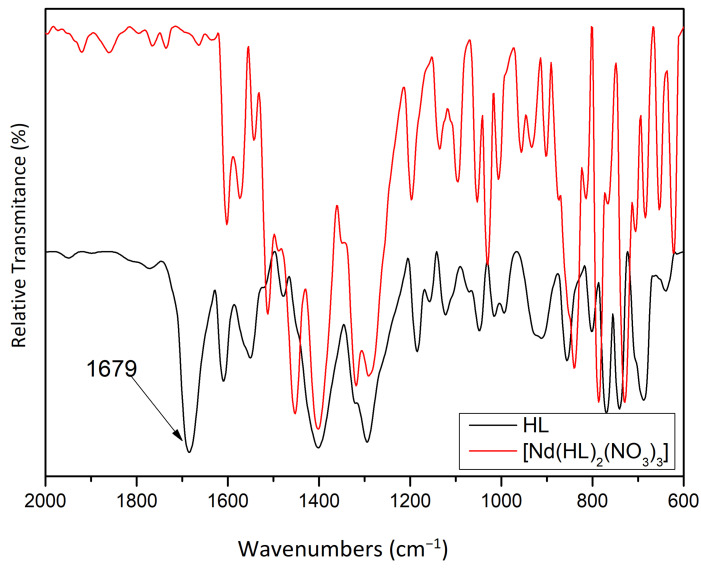
IR-spectra of **HL** and **[Nd(HL)_2_(NO_3_)_3_]** complex.

The ATR spectra of the synthesized lanthanide complexes **[Ln(HL)_2_(NO_3_)_3_]** are very similar and are presented in the Supplementary Materials (Figures S3–S9). The infrared spectra of the linker **HL** and **[Ln(HL)_2_(NO_3_)_3_]** (Ln = La, Nd, Eu, Gd, Dy, Ho) complexes show weak and medium absorption bands attributed to the asymmetric and symmetric C-H at 3123 cm^−1^ and 3084 cm^−1^ (for the HL) and 3136 cm^−1^, 3070 cm^−1^ (for the complexes), respectively. Additionally, the infrared spectra presented in Figures S3–S9 show week absorption bands between 2850 cm^−1^ and 2925 cm^−1^, which are residual artifacts from acetonitrile used as solvent. Also, as can be seen in Figure 1, the characteristic peak at 1679 cm^−1^ present in the IR spectrum of the HL is ascribed to ν(C-O), ν(O-H) stretching and δ(O-H) bending, corresponding to the COOH groups. The strong and medium absorption bands were observed in the range 1602–1460 cm^−1^ and are assigned to the aromatic stretch ν(C = C), ν(C = N), and asymmetric/symmetric vibrations of the complete deprotonation of the carboxylic acid (COO^−^). The bands from 1325 to 1300 cm^−1^ correspond to the ν_as_(NO_2_) vibrations of the coordinated nitrate [49], and bands at 1292 cm^−1^ are due to the ν(C = C) and ν(C-O) stretch. Furthermore, the strong band at 1031 cm^−1^ and medium bands at 684 and 625 cm^−1^, are assigned to Ln-O stretching vibrations for the mentioned complexes.

### 2.3. Powder X-ray Diffraction Studies (PXRD)

**Figure 2:**
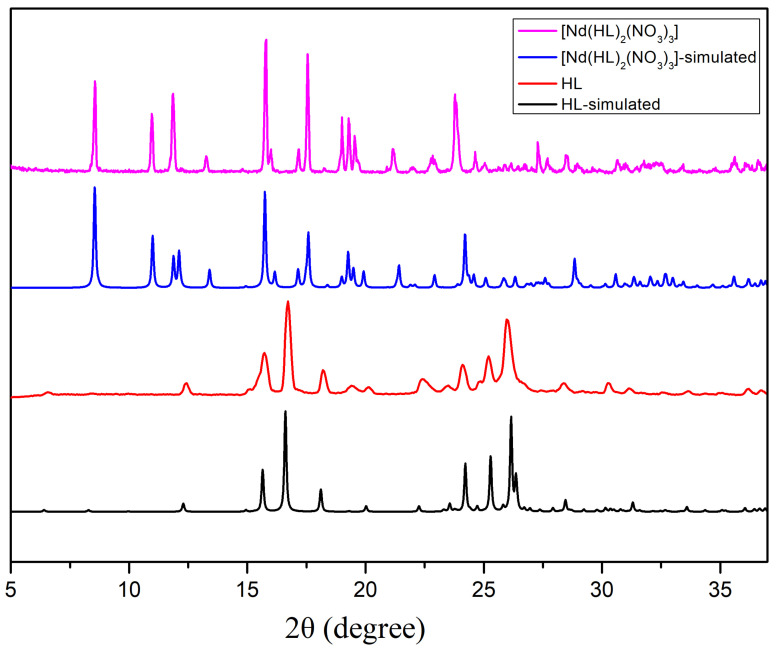
PXDR patterns of the synthesized **[Nd(HL)_2_(NO_3_)_3_]** and ligand **HL** in comparison with the simulated patterns from single crystal data.

The PXRD analysis of synthesized compounds provides important information about purity. All PXDR patterns were recorded after the compounds were washed, filtrated, air-dried and exposed to air at least one week. The powder X-ray diffraction patterns (PXRD) of the synthesized **HL** and **[Nd(HL)_2_(NO_3_)_3_]** are shown in Figure 2. The PXDR pattern of the **[Nd(HL)_2_(NO_3_)_3_]** presents diffraction patterns different from the PXDR pattern of ligand (**HL**), which indicates the formation of new complexes. Diffraction peaks appearing in lower angles for **3** and 2θ at 8.56, 10.96, 11.8, 13.2, 15.8 17.5 and 24.6 value is rather typical for coordination networks containing imidazole–biphenyl–carboxylate ligands as could be seen for other complex, for example **[ZnLAc]_n_** complex [50]. Furthermore, the diffraction peaks of the synthesized **[Ln(HL)_2_(NO_3_)_3_]** compounds are in good agreement with the simulated data, confirming that the **[Ln(HL)_2_(NO_3_)_3_]** were successfully synthesized as shown in Figure S2 (see Scheme 1, Strategy B). The powder materials obtained by the solvothermal method in ethanol and water as solvents (see Scheme 1, Strategy A) were impure, as shown in Figure S1 (Supplementary Materials), because PXRD analysis revealed supplementary peaks that corresponded to the ligand and other unidentified Ln-complexes.

### 2.4. Thermogravimetric Analysis (TGA)

**Figure 3:**
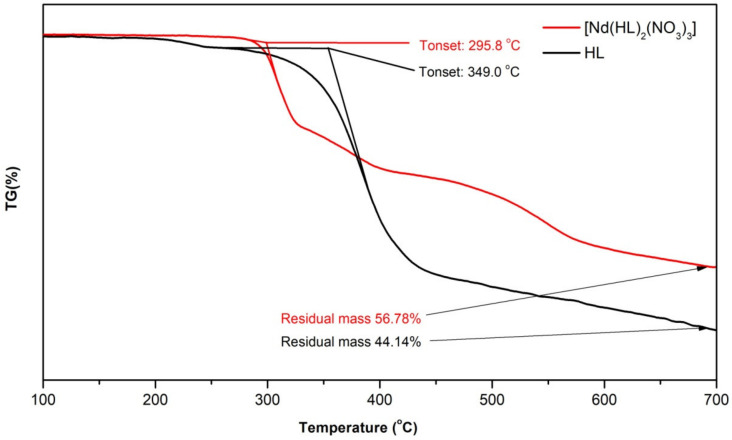
Thermogravimetric analysis of the synthesized **[Nd(HL)_2_(NO_3_)_3_]** and ligand **HL** (Tonset is established as the point of intersection of the starting-mass baseline and the tangent to the TGA curve at the point of maximum gradient).

The TGA analysis of synthesized materials was carried out from 30 to 700 °C, at a heating rate of 5 °C/min, under the nitrogen atmosphere. The TGA profiles of isostructural compounds **[Ln(HL)_2_(NO_3_)_3_]** (**1**–**9**) are quite similar, except small differences in the weight mass loss due to the differences in mass of the metal (see Supplementary Materials, Figures S10–S13). The TGA curves of **[Ln(HL)_2_(NO_3_)_3_]** show multi decomposition steps. Figure 3 presents the thermal decomposition of **[Nd(HL)_2_(NO_3_)_3_]** complex, compared to **HL**, as a representative example for explaining the dehydration and decomposition of all complexes: (i) the first stage was in the temperature range of 260–360 °C, and the percentage of mass loss is attributed to the release of three nitrate groups (inner ions) NO_3_^−^ (Found ~20 percent and calc. ~21 percent); (ii) the second stage corresponds to the loss of part of the ligand in the range between 360–500 °C (found ~9% mass loss); and (iii) the third stage until 700 °C reflected the loss of the other part of organic ligand (found ~13% mass loss). The residual weight of ~57% at 700 °C is attributed to the metal oxide and organic residue from the linker and above this temperature the decomposition is still continued (the framework continued to collapse with the loss of other molecules).

### 2.5. Luminiscence

**Figure 4:**
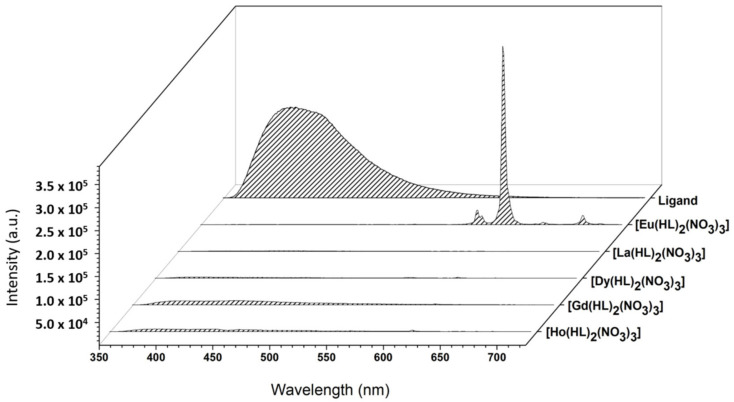
Emission spectra of five complexes and ligands obtained under excitation at 315 nm.

An important characteristic property of the complexes-class with lanthanides is the spectral narrow emission obtained from the Ln^3+^ 4f → 4f intra-atomic transitions and long radioactive lifetime [31,42]. Luminescence spectra of synthesized ligand and complexes with Ln^3+^ were recorded at room temperature as solid state. As can be seen in Figure 4, upon excitation at a wavelength of 315 nm, the ligand **HL** displays a broad fluorescence emission band between 356 nm and 556 nm with a maximum at 413 nm and a longer tail after the bigger wavelength value. The materials doped with Eu^3+^ exhibit ^5^D_0_ → ^7^F_J_ transition. Furthermore, we proved that the complex containing Eu^3+^ also exhibits as well ^5^D_0_ → ^7^F_4_ transition. Upon excitation at 315 nm, the **[Eu(HL)_2_(NO_3_)_3_]** exhibits characteristic sharp emissions at λ = 593 nm (weak, ^5^D_0_ → ^7^F_0_), λ = 597 nm (weak, ^5^D_0_ →^7^F_1_), λ = 615 nm (very strong, ^5^D_0_ → ^7^F_2_), λ = 651 nm (very weak, ^5^D_0_ → ^7^F_3_), and λ = 686 nm (very weak, ^5^D_0_ → ^7^F_4_). A high hypersensitivity of ^5^D_0_ → ^7^F_2_ transition at 615 nm (red light), followed by ^5^D_0_ → ^7^F_0_ transition at 593 nm, could be remarked in the spectra of **[Eu(HL)_2_(NO_3_)_3_]** complex. In addition, the luminescence characteristics of three additional complexes (**[Ln(HL)_2_(NO_3_)_3_]**, Ln = Dy, Gd, Ho) upon excitation at 315 nm (Figure S14) as well as excitation spectra at 440 nm (Figure S15) are shown in the Supplementary Materials. Besides, the emission band (350–560 nm) of the intermolecular transition of the ligand was not observed for the coordinated complexes. Experimentally, in this study, it was proved that the ligand and complexes display intense and narrow emissions at room temperature that make them good candidates for red and green emitter luminescence materials.

### 2.6. Crystallographic Studies

**Figure 5:**
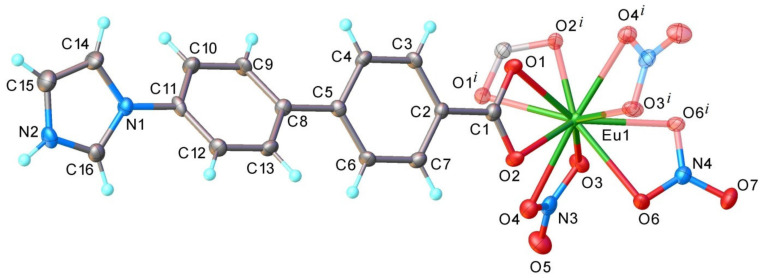
View of the asymmetric part of the unit cell in the crystal **4** with atom labeling and thermal ellipsoids at 50% level showing the coordination environment of Eu atom. Symmetry generated fragments are shown with faded colours. Symmetry code: 1.5 − *x*, *y*, 0.5 − *z*. Selected bond distances (Å): Eu-O1 2.512(2), Eu-O2 2.437(2), Eu-O3 2.508(2), Eu-O4 2.523(2), Eu-61 2.515(3).

**Figure 6:**
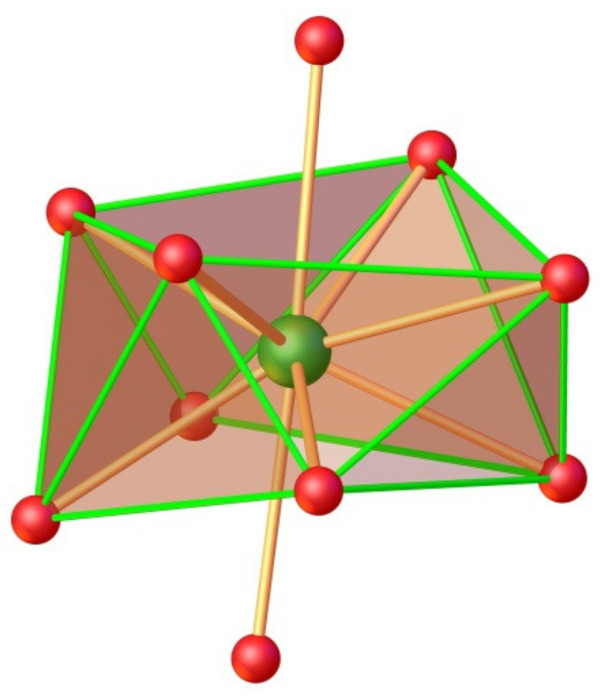
The coordination environment of the Eu atom. The triangular faces of square antiprism are drawn with green lines.

**Figure 7:**
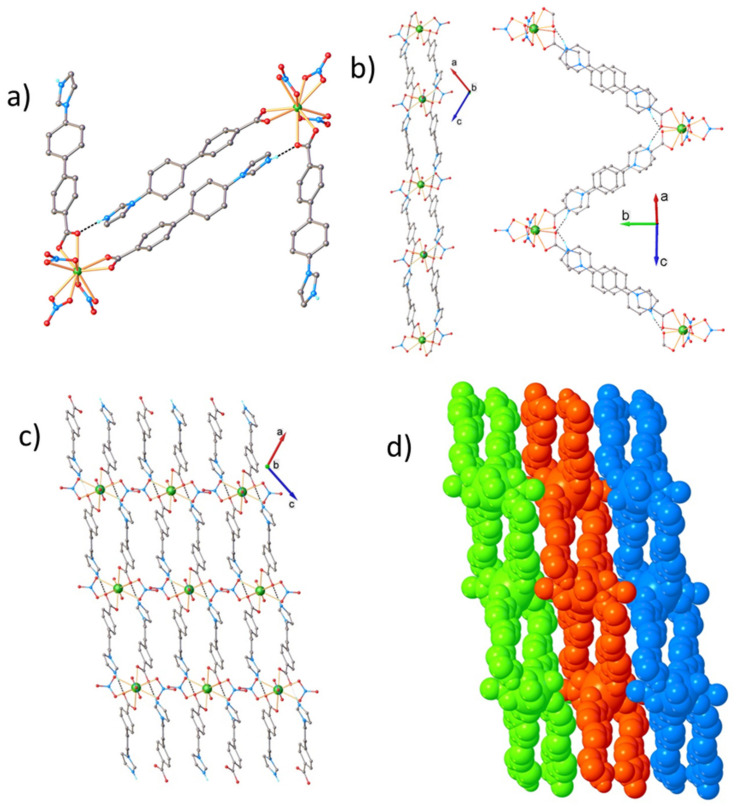
(**a**) The interaction of neutral **[Eu(HL)_2_(NO_3_)_3_]** complexes through N-H⋯O hydrogen bonds; (**b**) A view of zig-zag double chain along 010 and 101 directions; (**c**) Partial crystal packing viewed along *b* axis; (**d**) space filling representation showing the packing of 2D layers. Non-relevant H-atoms are omitted. H bond parameters: N2-H⋯O1 [N2-H 0.86 Å, H⋯O1 1.89 Å, N2⋯O1(*x* − 0.5, −*y*, 0.5 + *z*) 2.737(4)Å, ∠N2HO1 167.8°].

In order to elucidate the crystal structure of the synthesized compounds, a complete single-crystal X-ray diffraction study for **1**–**7** compounds have been carried out. According to X-ray crystallography the investigated compound form a series of isostructural crystals: they crystallize in *P2/n* space group of monoclinic system with close unit cell parameters (see Table 1). That is why, below, as an example, only the crystal structure for compound **[Eu(HL)_2_(NO_3_)_3_]** will be presented. The asymmetric part of the unit cell (Figure 5) comprises one Eu^3+^ cation, one imidazole–biphenyl–carboxylate zwitterion (**HL**) and one and a half NO_3_^−^ anions, so that the charge balance is in agreement with the formation of species **[Eu(HL)_2_(NO_3_)_3_]**. Three positive charges provided by Eu^3+^ ions are balanced by three negative charges of the nitrate anions acting as bidentate ligands. There no co-crystalized solvate molecules in **1**–**7** crystals. The **HL** zwitterion behaves as a non-bridging ligand being coordinated to Eu atom through carboxylate group in *к^2^O,O*′ bidentate-chelating mode. The Eu atom, located in special position on two-fold axis, is ten-coordinated by two carboxylate groups and three nitrate anions. It’s coordination polyhedral can be characterized as a distorted bicapped square antiprism in C_4v_ local geometry. A view of the coordination environment of Eu atom is shown in Figure 6. The further analysis of the crystal structure has shown, that non-coordinated nitrogen atom N2 is involved in the intermolecular hydrogen bonding towards carboxylate oxygen from adjacent unit (Figure 7a). As a result, the asymmetric units are assembled into one-dimensional supramolecular zig-zag double chains, as depicted in Figure 7b. The crystal structure is built up with the quite dense packing of 2D layers consisted from 1D double chains. The partial view of the crystal structure along *b* axis is depicted in Figure 7c,d.

## 3. Experimental Section

### 3.1. Material and Methods

All metal salts and solvents were purchased from Sigma Aldrich, Alfa Aesar, or other commercial sources, and used as received without additional purification. Fourier transform infrared (FT-IR) spectra were recorded on a Bruker Vertex 70 FTIR spectrometer (Bruker Optics GmbH, Ettlingen, Germany). The measurements were performed in ATR Golden Gate^®^ (Attenuated Total Reflectance) mode in the 600–4000 cm^−1^ range at room temperature with a resolution of 4 cm^−1^ over a total of 64 scans. Thermogravimetric analyses (TGA) were performed under nitrogen stream with a flow rate of 50 mL min^−1^ using a STA 449 F1 Jupiter (Netzsch, GmbH, Selb, Germany) device. The temperature range used for measurements was between 30 to 700 °C with a heating rate of 5 °C min^−1^ and the data was processed with the NETZSCH Proteus 4.2 software. The Powder X-ray diffraction patterns (PXRD) were collected employing a Bruker AXS D8 Phaser powder diffractometer with a monochromated Cu-Kα (k = 1.5418 Å) radiation in Bragg-Brentano arrangement, with scan speed of 4 s/step and a step size of 0.02° (2θ) at RT in the range of 2θ = 3.5–50°. Simulated PXRD patterns were calculated with CCDC Mercury 4.1 program using the single-crystal data of the compounds. CHN elemental analysis was performed on a VARIO-EL-III elemental analyzer. The photoluminescence spectra were measured for solid state of complexes at room temperature on a Horiba Fluoromax-4 fluorescence spectroscopy instrument using a 370 nm long pass filter from Edmund Optics.

### 3.2. General Procedure for [Ln(HL)_2_(NO_3_)_3_]

The organic linker **HL** (4′-(1*H*-imidazol-1-yl)biphenyl-4-carboxylic acid) was prepared using a slightly modified procedure described by our group and NMR; IR spectroscopy was used to establish its structure and purity [48]. Starting with the functionalized aromatic building blocks **HL**, the desired products **[Ln(HL)_2_(NO_3_)_3_]** ( Ln = La, Ce, Nd, Eu, Gd, Dy, Ho) were prepared using two different strategies:

Strategy A: A mixture of Ln(NO_3_)_3_·yH_2_O (0.3 mmol; Ln = La, Nd, Gd, Dy, Ho), HL (0.1 mmol), ethanol (1 mL) and water (0.5 mL) was transferred to a 10 mL culture tub with a PTFE-lined PBT screw and kept under static conditions for two to five days at 80 °C. After cooling, the crystalline product formed was filtered, washed with ethanol (2 mL), and then dried under air. The materials obtained were investigated by PXRD and from them, only three crystals (La, Nd, Dy) were suitable for single-crystal X-ray diffraction analysis. Strategy B: A mixture of Ln(NO_3_)_3_·yH_2_O (0.3 mmol; Ln = La, Ce, Nd, Eu, Gd, Dy, Ho), HL (0.1 mmol), acetonitrile (10 mL) and water (1 mL) was transferred to a 10 mL culture tub with a PTFE-lined PBT screw. The tube with suspension was kept under static conditions in a controlled Memmert oven, for two days at 120 °C. The heating and cooling programs were run with a heating/cooling rate of 1 °C/min. At the end of the reaction time, the crystals were collected by filtration or centrifugation, and washed with acetonitrile three times. Finally, the colored compound (yellow to brawn) was allowed to dry at room temperature. The amount, stoichiometry and yields (based on the linker) are given in Table 2.

#### Synthesis of the Lanthanide (III) Complexes

**[La(HL)_2_(NO_3_)_3_] (1):** Dark yellow; Yield 46 %; Anal. Calc. for C_32_H_24_LaO_13_N_7_: C 45.03, H 2.83, N 11.49 %. Found: C 44.80, H 2.70, N 11.30%; IR (ATR): ν (cm^−1^) = 621.04 (m); 653.83 (m); 682.76 (m); 705.9 (m); 729.05 (vs); 765.69 (m); 786.91 (vs); 815.84 (m); 840.91 (s); 873.70 (m); 900.70 (m); 931.56 (m); 956.63 (m); 1006.78 (m); 1029.92 (s); 1053.07 (m); 1134.07 (m); 1195.79 (m); 1280.65 (s); 1290.30 (s); 1315.37 (s); 1134.07 (m); 1195.79 (m); 1280.65 (s); 1290.30 (s); 1315.307 (s); 1510.17 (s); 1542.96 (m); 1573.82 (m); 1602.75 (m); 2854.47 (w); 2923.90 (w); 3070.49 (w); 3136.06 (m).

**[Ce(HL)_2_(NO_3_)_3_] (2):** Yellowish; Yield 64 %; Anal. Calc. for C_32_H_24_CeO_13_N_7_: C 44.97, H 2.83, N 11.47 %. Found: C 44.85, H 2.75, N 11.38 %.

**[Nd(HL)_2_(NO_3_)_3_] (3):** Yellow; Yield 25%; Yield 62%; Anal. Calc. for C_32_H_24_NdO_13_N_7_: C 44.75, H 2.82, N 11.42 %. Found: C 44.65, H 2.78, N 11.27 %; IR (ATR): ν (cm^−1^) = 622.97 (m); 653.83 (m); 684.69 (m); 705.9 (m); 729.05 (vs); 767.62 (m); 786.91 (vs); 813.91 (m); 840.91 (s); 873.70 (m); 902.70 (m); 933.49 (m); 956.63 (m); 1006.78 (m); 1029.92 (m); 1053.07 (m); 1095.50 (m); 1136.00 (m); 1197.72 (m); 1290.30 (s); 1319.23 (s); 1350.09 (m); 1402.16 (vs); 1452.31 (vs); 1488.95 (m); 1512.10 (s); 1542.96 (m); 1573.82 (m); 1602.75 (m); 2854.47 (w); 2925.83 (w); 3070.49 (w); 3134.06 (m).

**[Eu(HL)_2_(NO_3_)_3_] (4):** Brown; Yield 49%; Anal. Calc. for C_32_H_24_EuO_13_N_7_: C 44.35, H 2.79, N 11.31 %. Found: C 44.18, H 2.60, N 11.12 %; IR (ATR): ν (cm^−1^) = 621.04 (m); 653.83 (m); 684.69 (m); 705.9 (m); 730.98 (vs); 767.62 (m); 786.91 (vs); 813.91 (m); 840.91 (s); 900.7 (m); 935.43 (m); 956.63 (m); 1006.78 (m); 1031.85 (s); 1053.07 (m); 1095.5 (m); 1136.00 (m); 1097.72 (m); 1292.23 (vs); 1323.09 (s); 1404.09 (vs); 1454.24 (vs); 1488.95 (m); 1515.95 (s); 1542.96 (m); 1573.82 (m); 1602.75 (m); 2854.47 (w); 2923.9 (w); 3070.49 (w); 3134.13 (m).

**[Gd(HL)_2_(NO_3_)_3_] (5)**: Brown; Yield 47%; Anal. Calc. for C_32_H_24_GdO_13_N_7_: C 44.08, H 2.77, N 11.25 %. Found: C 42.87, H 2.70, N 11.10%; IR (ATR): ν (cm^−1^) = 622.97 (m); 653.83 (m); 684.69 (m); 705.9 (m); 730.98 (vs); 767.62 (m); 768.91 (vs); 813.91 (m); 840.91 (vs); 854.41 (s); 902.63 (m); 935.42 (m); 956.63 (m); 1006.78 (m); 1031.85 (s); 1053.07 (m); 1095.5 (m); 1136.00 (m); 1197.72 (m); 1292.23 (s); 1325.01 (s); 1406.02 (vs); 1454.24 (vs); 1488.95 (m); 1515.96 (s); 1542.96 (m); 1575.75 (m); 1602.75 (m); 2854.47 (w); 2923.90 (w); 3070.49 (w); 3134.13 (m).

**[Dy(HL)_2_(NO_3_)_3_] (6)**: Dark yellow; Yield 43%; Anal. Calc. for C_32_H_24_DyO_13_N_7_: C 43.82, H 2.76, N 11.18 %. Found: C 42.51, H 2.60, N 11.09%; IR (ATR): ν (cm^−1^) = 622.97 (m); 653.83 (m); 684.69 (m); 705.9 (m); 729.05 (s);769.55 (m); 786.91 (vs); 813.91 (m); 840.91 (s); 862.13 (s); 902.63 (m); 937.35 (m); 956.63 (m); 1006.78 (m); 1033.78 (m); 1053.07 (m); 1095.50 (m); 1136.00 (m); 1197.72 (m); 1292.23 (s); 1325.01 (s); 1406.02 (vs); 1454.24 (vs); 1490.88 (s); 1515.96 (s); 1542.96 (m); 1575.75 (m); 1602.75 (m); 2854.47 (w); 2923.90 (w); 3070.49 (w); 3134.13 (m).

**[Ho(HL)_2_(NO_3_)_3_] (7)**: Dark yellow; Yield 25%; Anal. Calc. for C_32_H_24_HoO_13_N_7_: C 43.70, H 2.75, N 11.15 %. Found: C 42.58, H 2.70, N 11.03%; IR (ATR): ν (cm^−1^) = 622.97 (m); 653.83 (m); 684.69 (m); 705.9 (m); 729.05 (s); 786.91 (vs); 813.91 (m); 840.91 (s); 862.13 (s); 902.63 (m); 937.35 (m); 956.63 (m); 1006.78 (m); 1033.78 (m); 1055.00 (m); 1095.50 (m); 1136.00 (w); 1197.72 (m); 1294.16 (s); 1328.87 (s); 1407.95 (vs); 1456.17 (vs); 1490.88 (s); 1517.88 (s); 1542.96 (m); 1575.75 (m); 1602.75 (m); 2854.47 (w); 2923.90 (w); 3070.49 (w); 3134.13 (w); 3155.35 (w).

### 3.3. Single Crystal X-ray Diffraction

X-ray diffraction measurements were carried out with a Rigaku Oxford-Diffraction XCALIBUR E CCD diffractometer equipped with graphite-monochromated MoKα radiation. The unit cell determination and data integration were carried out using the CrysAlis package of Oxford Diffraction [51]. The structures were solved by Intrinsic Phasing using Olex2 [52] software with the SHELXT [53] structure solution program and refined by full-matrix least-squares on F^2^ with SHELXL-2015 [54] using an anisotropic model for non-hydrogen atoms. All H atoms were introduced in idealized positions (d_CH_ = 0.96 Å) using the riding model. The H-atoms attached to nitrogen were localized from difference Fourier maps and their positional parameters verified according to H-bonds geometry. The molecular plots were obtained using the Olex2 program. The crystallographic data and refinement details are quoted in Table 1, while bond lengths and angles are given in the Supplementary Materials. CCDC-2107718-2107724 for **1**–**7**, respectively, contain the Supplementary Crystallographic Data for this contribution. These data can be obtained free of charge via www.ccdc.cam.ac.uk/conts/retrieving.html (or from the Cambridge Crystallographic Data Centre, 12 Union Road, Cambridge CB2 1EZ, UK; Fax: (+44) 1223-336-033; or deposit@ccdc.ca.ac.uk).

## 4. Conclusions

This work was focused on the structure, thermal stability, and luminescence properties of lanthanides complexes with an imidazole–biphenyl–carboxylate ligand. Here, we report the optimized synthesis conditions of a series of neutral seven coordinate lanthanide complexes. These complexes were obtained by solvothermal reactions of simple salts with acid ligands, in good yields and high purity. According to X-ray crystallography, the studied complexes form a series of isostructural **[Ln(HL)_2_(NO_3_)_3_]** molecular species. Furthermore, the complex with Eu^3+^ shows good luminescence properties, and this could be further developed, investigated and applied in medical imaging.

## Figures and Tables

**Table 1:** Crystal data and details of data collection.

Parameter	Compound						
	1	2	3	4	5	6	7
Empirical Formula	C_32_H_24_LaN_7_O_13_	C_32_H_24_CeN_7_O_13_	C_32_H_24_N_7_NdO_13_	C_32_H_24_EuN_7_O_13_	C_32_H_24_GdN_7_O_13_	C_32_H_24_DyN_7_O_13_	C_32_H_24_HoN_7_O_13_
*Fw*	853.49	854.70	858.82	866.54	871.83	877.08	879.51
Space Group	*P*2/*n*	*P*2/*n*	*P*2/*n*	*P*2/*n*	*P*2/*n*	*P*2/*n*	*P*2/*n*
*a* [Å]	11.7434(7)	11.6491(5)	11.6264(8)	11.6229(7)	11.6154(7)	11.5987(4)	11.6138(3)
*b* [Å]	10.0978(5)	10.1346(4)	10.1166(6)	10.0972(5)	10.0797(5)	10.0803(3)	10.0845(2)
*c* [Å]	14.0667(9)	14.0436(7)	14.0056(8)	14.0020(8)	13.9905(7)	13.9615(5)	14.0087(4)
*α* [°]	90	90	90	90	90	90	90
*β* [°]	108.771(7)	109.849(5)	109.845(7)	109.798(7)	109.817(7)	109.871(4)	109.726(3)
*γ* [°]	90	90	90	90	90	90	90
*V* [Å^3^]	1579.36(17)	1559.48(13)	1549.51(17)	1546.14(16)	1541.00(16)	1535.17(10)	1544.41(7)
*Z*	2	2	2	2	2	2	2
*r*_calcd_ [g cm^−3^]	1.795	1.820	1.841	1.861	1.879	1.897	1.891
Crystal Size [mm]	0.25 × 0.05 × 0.02	0.20 × 0.10 × 0.05	0.25 × 0.10 × 0.05	0.30 × 0.05 × 0.05	0.30 × 0.05 × 0.05	0.10 × 0.05 × 0.05	0.25 × 0.05 × 0.05
*T* [K]	293	180	180	180	180	180	180
*μ* [mm^−1^]	1.436	1.544	1.761	2.114	2.238	2.520	2.647
2Θ range [°]	4.034 to 50.054	3.942 to 58.712	3.952 to 50.052	3.954 to 58.262	3.956 to 50.048	3.962 to 50.054	3.956 to 50.044
Reflections Collected	5552	7773	5738	7681	5840	11,123	22,162
Independent Reflections	2786[*R*_int_ = 0.0333]	3657[*R*_int_ = 0.0356]	2743[*R*_int_ = 0.0401]	3599[*R*_int_ = 0.0387]	2712[*R*_int_ = 0.0267]	2713[*R*_int_ = 0.0431]	2727[*R*_int_ = 0.0557]
Data/Restraints/Parameters	2786/0/241	3657/0/241	2743/0/241	3599/0/241	2712/0/241	2713/0/241	2727/0/241
*R*_1_ ^a^	0.0326	0.0341	0.0365	0.0419	0.0300	0.0301	0.0304
*wR*_2_ ^b^	0.0483	0.0668	0.0609	0.0700	0.0467	0.0622	0.0764
GOF ^c^	0.976	1.050	1.007	1.042	1.057	1.070	1.040
Largest Diff. Peak/Hole [e Å^−3^]	0.51/−0.37	0.63/−0.50	0.48/−0.76	0.82/−0.71	0.49/−0.42	0.55/−0.60	0.86/−0.66

^a^ *R*_1_ = Σ||*F*_o_| − |*F*_c_||/Σ|*F*_o_|. ^b^ *wR*_2_ = {Σ[*w*(*F*_o_^2^ − *F*_c_^2^)^2^]/Σ[*w*(*F*_o_^2^)^2^]}^1/2^. ^c^ GOF = {Σ[*w*(*F*_o_^2^ − *F*_c_^2^)^2^]/(*n* − *p*)}^1/2^, where *n* is the number of reflections and *p* is the total number of parameters refined.

**Table 2:** An overview of the optimized synthesis conditions for the all [Ln(HL)_2_(NO_3_)_3_].

Compound	Strategy	Metal Source	Ligand (mmol)	Salt (mmol)	EtOH (mL)	MeCN (mL)	H_2_O (mL)	Yield/ Color
**[La(HL)_2_(NO_3_)_3_] (1)**	A	La(NO_3_)_3_·6H_2_O	0.06	0.17	3.80	-	1.0	47%/ Yellow **(Mixture)**
**[La(HL)_2_(NO_3_)_3_] (1)**	B	La(NO_3_)_3_·6H_2_O	0.026	0.82	-	28.6	2.25	46%/ Dark Yellow
**[Ce(HL)_2_(NO_3_)_3_] (2)**	B	Ce(NO_3_)_3_·6H_2_O	0.026	0.72	-	3.20	0.25	64%/ Yellowish
**[Nd(HL)_2_(NO_3_)_3_] (3)**	A	Nd(NO_3_)_3_·6H_2_O	0.03	0.09	1.90	-	-	82% Yellowish **(Mixture)**
**[Nd(HL)_2_(NO_3_)_3_] (3)**	B	Nd(NO_3_)_3_·6H_2_O	0.15	0.50	-	19.0	1.5	25%/ Yellow
**[Eu(HL)_2_(NO_3_)_3_] (4)**	B	Eu(NO_3_)_3_·5H_2_O	0.18	0.53	-	28.6	2.25	49%/ Brown
**[Gd(HL)_2_(NO_3_)_3_] (5)**	A	Gd(NO_3_)_3_·6H_2_O	0.08	0.25	1.30	-	1.0	3%/ Yellow **(Mixture)**
**[Gd(HL)_2_(NO_3_)_3_] (5)**	B	Gd(NO_3_)_3_·6H_2_O	0.19	0.53	-	28.6	2.25	47%/ Brown
**[Dy(HL)_2_(NO_3_)_3_] (6)**	A	Dy(NO_3_)_3_·5H_2_O	0.12	0.36	1.90	-	0.50	29%/ Yellow **(Mixture)**
**[Dy(HL)_2_(NO_3_)_3_] (6)**	B	Dy(NO_3_)_3_·5H_2_O	0.12	0.37	-	9.50	0.75	43%/ Dark Yellow
**[Ho(HL)_2_(NO_3_)_3_] (7)**	A	Ho(NO_3_)_3_·5H_2_O	0.02	0.06	1.90	-	-	75%/ Yellow **(Mixture)**
**[Ho(HL)_2_(NO_3_)_3_] (7)**	B	Ho(NO_3_)_3_·5H_2_O	0.12	0.34	-	19.0	1.50	25%/ Dark Yellow

## Data Availability

Supplementary Crystallographic Data can be obtained free of charge via www.ccdc.cam.ac.uk/conts/retrieving.html (or from the Cambridge Crystallographic Data Centre, 12 Union Road, Cambridge CB2 1EZ, UK; Fax: (+44) 1223-336-033; or deposit@ccdc.ca.ac.uk).

## Supplementary Materials

The following are available online; Figure S1: PXRD patterns of the isostructural compounds versus simulated powder PXRD pattern generated from a single crystal cif file of **[Nd(HL)_2_(NO_3_)_3_]** (**3**) compound (in EtOH/H_2_O as presented in Scheme 1, Strategy A); Figure S2: PXRD patterns of the isostructural compounds versus simulated powder XRD pattern generated from a single crystal cif file of **[Nd(HL)_2_(NO_3_)_3_]** (**3**) compound (in CH_3_CN/H_2_O as presented in Scheme 1, Strategy B); Figure S3: IR-spectrum of 4′-(1*H*-imidazol-1-yl)biphenyl-4-carboxylic acid (**HL**); Figure S4: IR-spectra of **[Ln(HL)_2_(NO_3_)_3_]** (Ln = La, Nd, Dy, Eu, Gd, Ho); Figure S5: IR-spectrum of **[La(HL)_2_(NO_3_)_3_]**; Figure S6: IR-spectrum of **[Nd(HL)_2_(NO_3_)_3_]**; Figure S7: IR-spectrum of **[Gd(HL)_2_(NO_3_)_3_]**; Figure S8: IR-spectrum of **[Dy(HL)_2_(NO_3_)_3_]**; Figure S9: IR-spectrum of **[Ho(HL)_2_(NO_3_)_3_]**; Figure S10: Thermogravimetric analysis of compound **[Nd(HL)_2_(NO_3_)_3_]** (**3**). Figure S11: Thermogravimetric analysis of compound **[Gd(HL)_2_(NO_3_)_3_]** (**5**); Figure S12: Thermogravimetric analysis of compound **[Dy(HL)_2_(NO_3_)_3_]** (**6**); Figure S13: Thermogravimetric analysis of compound **[Ho(HL)_2_(NO_3_)_3_]** (**7**); Figure S14: Emission spectra of three complexes (Gd, Ho, Dy) obtained under excitation at 315 nm; Figure S15: Excitation spectra of three complexes (Eu, Gd, Ho) obtained at 440 nm (2D chart); CIFreport_Ln1: ellipsoid plots of all **[Ln(HL)_2_(NO_3_)_3_]** (Ln = La, Nd, Dy, Eu, Gd, Ho); Table with bond distances (Å) and angles (°) for shI_4082_BeDa; Table with bond distances (Å) and angles (°) for MD_4164_BeDa; Table with bond distances (Å) and angles (°) for shI_4078_BeDa; Table with bond distances (Å) and angles (°) for MD_4167_BeDa; Table with bond distances (Å) and angles (°) for MD_4168_BeDa; Table with bond distances (Å) and angles (°) for shI_4108_BeDa; Table with bond distances (Å) and angles (°) for shI_4151_BeDa.

## Sample Availability

Samples of the compounds are not available from the authors.
