# Supplementary material for: Novel Lanthanide (III) Complexes Derived from an Imidazole–Biphenyl–Carboxylate Ligand: Synthesis, Structure and Luminescence Properties
Source: Molecules. 2021 Nov 17;26(22):6942. doi: 10.3390/molecules26226942 (PMC8625298; doi:10.3390/molecules26226942)

checkCIF/PLATON report

Structure factors have been supplied for datablock(s) shi_4082_beda

THIS REPORT IS FOR GUIDANCE ONLY. IF USED AS PART OF A REVIEW PROCEDURE FOR PUBLICATION, IT SHOULD NOT REPLACE THE EXPERTISE OF AN EXPERIENCED CRYSTALLOGRAPHIC REFEREE.

No syntax errors found. CIF dictionary Interpreting this report

Datablock: shi_4082_beda

```
Bond precision:   C-C = 0.0045 A          Wavelength=0.71073

Cell:            a=11.7434(7)     b=10.0978(5)       c=14.0667(9)
                 alpha=90         beta=108.771(7)    gamma=90
Temperature:     293 K

                Calculated                  Reported
Volume          1579.34(17)                 1579.36(17)
Space group     P 2/n                       P 1 2/n 1
Hall group      -P 2yac                     -P 2yac
Moiety formula  C32 H24 La N7 O13           C32 H24 La N7 O13
Sum formula     C32 H24 La N7 O13           C32 H24 La N7 O13
Mr              853.49                      853.49
Dx,g cm-3       1.795                       1.795
Z               2                           2
Mu (mm-1)       1.436                       1.436
F000            852.0                       852.0
F000'           851.84
h,k,lmax        13,12,16                    13,12,16
Nref            2790                        2786
Tmin,Tmax       0.917,0.972                 0.991,1.000
Tmin'           0.698

Correction method= # Reported T Limits: Tmin=0.991 Tmax=1.000
AbsCorr = MULTI-SCAN

Data completeness= 0.999           Theta(max)= 25.027

                                                  wR2(reflections)=
R(reflections)= 0.0326( 2372)                     0.0483( 2786)

S = 0.976                  Npar= 241
```

The following ALERTS were generated. Each ALERT has the format

test-name_ALERT_alert-type_alert-level.

Click on the hyperlinks for more details of the test.

Alert level C

```
PLAT230_ALERT_2_C Hirshfeld Test Diff for    O3        --N3       .         5.5 s.u.
PLAT230_ALERT_2_C Hirshfeld Test Diff for    O4        --N3       .         6.0 s.u.
PLAT906_ALERT_3_C Large K Value in the Analysis of Variance ......        2.073 Check
PLAT911_ALERT_3_C Missing FCF Refl Between Thmin & STh/L=    0.595          3 Report
```

Alert level G

```
PLAT007_ALERT_5_G Number of Unrefined Donor-H Atoms .............           1 Report
PLAT199_ALERT_1_G Reported _cell_measurement_temperature ..... (K)        293 Check
PLAT200_ALERT_1_G Reported   _diffrn_ambient_temperature ..... (K)        293 Check
PLAT232_ALERT_2_G Hirshfeld Test Diff (M-X)  La1       --O4       .         9.2 s.u.
PLAT232_ALERT_2_G Hirshfeld Test Diff (M-X)  La1       --O6       .         7.4 s.u.
PLAT432_ALERT_2_G Short Inter X...Y Contact  O5        ..C16               2.91 Ang.
                                                  1+x,1+y,z  =      1_665 Check
PLAT794_ALERT_5_G Tentative Bond Valency for La1        (III)     .        3.71 Info
PLAT909_ALERT_3_G Percentage of I>2sig(I) Data at Theta(Max) Still          80% Note
PLAT910_ALERT_3_G Missing # of FCF Reflection(s) Below Theta(Min).           1 Note
PLAT933_ALERT_2_G Number of OMIT Records in Embedded .res File ...           3 Note
PLAT941_ALERT_3_G Average HKL Measurement Multiplicity ..........         2.0 Low
PLAT978_ALERT_2_G Number C-C Bonds with Positive Residual Density.           1 Info
```

0 **ALERT level A** = Most likely a serious problem - resolve or explain
0 **ALERT level B** = A potentially serious problem, consider carefully
4 **ALERT level C** = Check. Ensure it is not caused by an omission or oversight
12 **ALERT level G** = General information/check it is not something unexpected

2 ALERT type 1 CIF construction/syntax error, inconsistent or missing data
7 ALERT type 2 Indicator that the structure model may be wrong or deficient
5 ALERT type 3 Indicator that the structure quality may be low
0 ALERT type 4 Improvement, methodology, query or suggestion
2 ALERT type 5 Informative message, check

It is advisable to attempt to resolve as many as possible of the alerts in all categories. Often the minor alerts point to easily fixed oversights, errors and omissions in your CIF or refinement strategy, so attention to these fine details can be worthwhile. In order to resolve some of the more serious problems it may be necessary to carry out additional measurements or structure refinements. However, the purpose of your study may justify the reported deviations and the more serious of these should normally be commented upon in the discussion or experimental section of a paper or in the "special_details" fields of the CIF. checkCIF was carefully designed to identify outliers and unusual parameters, but every test has its limitations and alerts that are not important in a particular case may appear. Conversely, the absence of alerts does not guarantee there are no aspects of the results needing attention. It is up to the individual to critically assess their own results and, if necessary, seek expert advice.

Publication of your CIF in IUCr journals

A basic structural check has been run on your CIF. These basic checks will be run on all CIFs submitted for publication in IUCr journals (*Acta Crystallographica*, *Journal of Applied Crystallography*, *Journal of Synchrotron Radiation*); however, if you intend to submit to *Acta Crystallographica Section C* or *E* or *IUCrData*, you should make sure that full publication checks are run on the final version of your CIF prior to submission.

Publication of your CIF in other journals

Please refer to the *Notes for Authors* of the relevant journal for any special instructions relating to CIF submission.

PLATON version of 13/07/2021; check.def file version of 13/07/2021

Datablock shi_4082_beda - ellipsoid plot

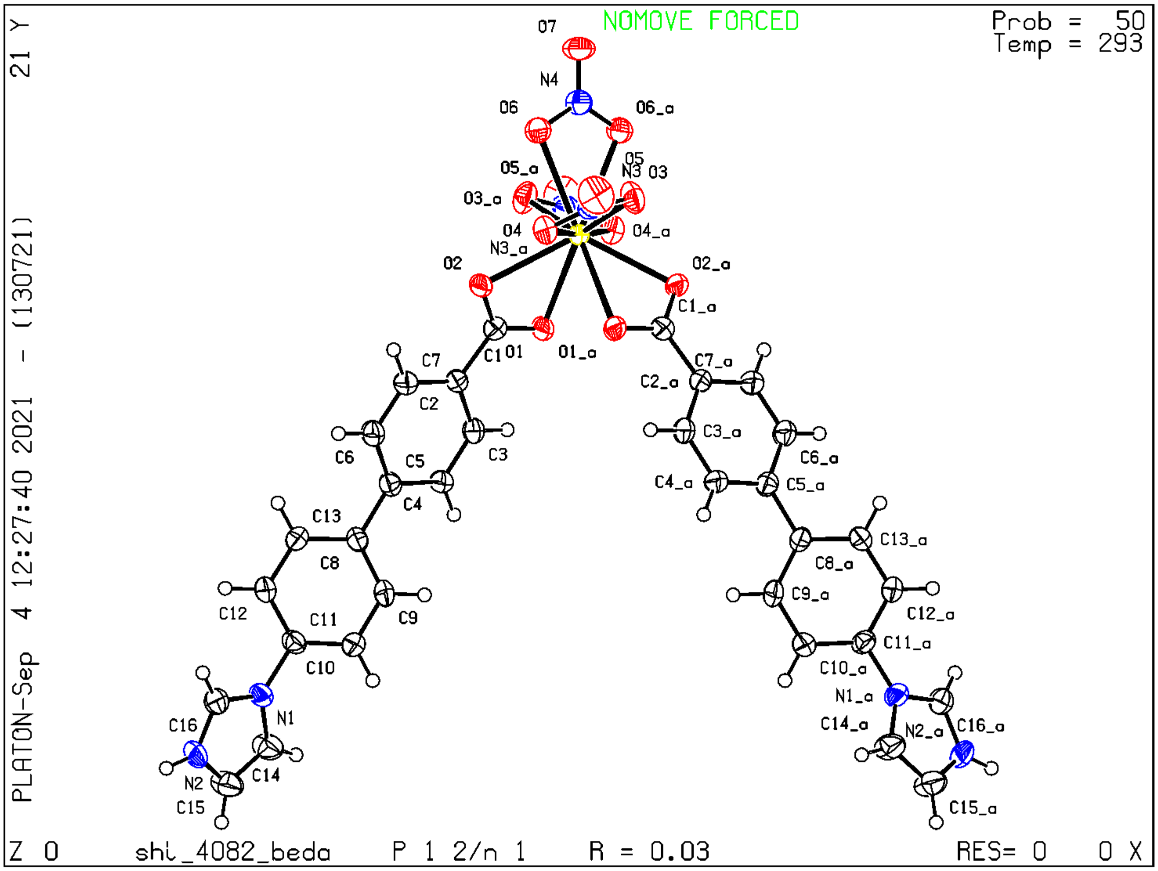

checkCIF/PLATON report

Structure factors have been supplied for datablock(s) md_4164_beda

THIS REPORT IS FOR GUIDANCE ONLY. IF USED AS PART OF A REVIEW PROCEDURE FOR PUBLICATION, IT SHOULD NOT REPLACE THE EXPERTISE OF AN EXPERIENCED CRYSTALLOGRAPHIC REFEREE.

No syntax errors found. CIF dictionary Interpreting this report

Datablock: md_4164_beda

Bond precision: C-C = 0.0041 A Wavelength=0.71073

Cell:	a=11.6491(5)	b=10.1346(4)	c=14.0436(7)
	alpha=90	beta=109.849(5)	gamma=90
Temperature:	180 K		

	Calculated	Reported
Volume	1559.47(13)	1559.48(13)
Space group	P 2/n	P 1 2/n 1
Hall group	-P 2yac	-P 2yac
Moiety formula	C32 H24 Ce N7 O13	C32 H24 Ce N7 O13
Sum formula	C32 H24 Ce N7 O13	C32 H24 Ce N7 O13
Mr	854.70	854.70
Dx,g cm-3	1.820	1.820
Z	2	2
Mu (mm-1)	1.544	1.544
F000	854.0	854.0
F000'	853.91	
h,k,lmax	16,13,19	15,13,18
Nref	4291	3657
Tmin,Tmax	0.831,0.926	0.759,1.000
Tmin'	0.734	

Correction method= # Reported T Limits: Tmin=0.759 Tmax=1.000
AbsCorr = MULTI-SCAN

Data completeness= 0.852 Theta(max)= 29.356

R(reflections)= 0.0341(3232) wR2(reflections)= 0.0668(3657)

S = 1.049 Npar= 241

The following ALERTS were generated. Each ALERT has the format
test-name_ALERT_alert-type_alert-level.
Click on the hyperlinks for more details of the test.

Alert level C

```
PLAT906_ALERT_3_C Large K Value in the Analysis of Variance ......      2.257 Check
```

Alert level G

```
PLAT007_ALERT_5_G Number of Unrefined Donor-H Atoms .............          1 Report
PLAT232_ALERT_2_G Hirshfeld Test Diff (M-X)  Ce1      --O3       .        5.5 s.u.
PLAT232_ALERT_2_G Hirshfeld Test Diff (M-X)  Ce1      --O4       .        5.8 s.u.
PLAT232_ALERT_2_G Hirshfeld Test Diff (M-X)  Ce1      --O6       .        6.6 s.u.
PLAT432_ALERT_2_G Short Inter X...Y Contact  O5       ..C16              2.90 Ang.
                                    3/2-x,-1+y,3/2-z  =      2_646 Check
PLAT779_ALERT_4_G Suspect or Irrelevant (Bond) Angle(s) in CIF ...       0.00 Deg.
              N4   -O6   -N4      2_556   1_555   1_555 ......     #   52 Check
PLAT779_ALERT_4_G Suspect or Irrelevant (Bond) Angle(s) in CIF ...       0.00 Deg.
              N4   -O7   -N4      2_556   1_555   1_555 ......     #   53 Check
PLAT779_ALERT_4_G Suspect or Irrelevant (Bond) Angle(s) in CIF ...       0.00 Deg.
              N4   -N4   -O6      2_556   1_555   2_556 ......     #   66 Check
PLAT779_ALERT_4_G Suspect or Irrelevant (Bond) Angle(s) in CIF ...       0.00 Deg.
              N4   -N4   -O6      2_556   1_555   1_555 ......     #   67 Check
PLAT779_ALERT_4_G Suspect or Irrelevant (Bond) Angle(s) in CIF ...       0.00 Deg.
              N4   -N4   -O7      2_556   1_555   1_555 ......     #   68 Check
PLAT794_ALERT_5_G Tentative Bond Valency for Ce1       (III)     .       3.22 Info
PLAT912_ALERT_4_G Missing # of FCF Reflections Above STh/L=  0.600        617 Note
PLAT941_ALERT_3_G Average HKL Measurement Multiplicity ..........        2.1 Low
PLAT978_ALERT_2_G Number C-C Bonds with Positive Residual Density.          6 Info
```

   0 **ALERT level A** = Most likely a serious problem - resolve or explain
   0 **ALERT level B** = A potentially serious problem, consider carefully
   1 **ALERT level C** = Check. Ensure it is not caused by an omission or oversight
  14 **ALERT level G** = General information/check it is not something unexpected

   0 ALERT type 1 CIF construction/syntax error, inconsistent or missing data
   5 ALERT type 2 Indicator that the structure model may be wrong or deficient
   2 ALERT type 3 Indicator that the structure quality may be low
   6 ALERT type 4 Improvement, methodology, query or suggestion
   2 ALERT type 5 Informative message, check

It is advisable to attempt to resolve as many as possible of the alerts in all categories. Often the minor alerts point to easily fixed oversights, errors and omissions in your CIF or refinement strategy, so attention to these fine details can be worthwhile. In order to resolve some of the more serious problems it may be necessary to carry out additional measurements or structure refinements. However, the purpose of your study may justify the reported deviations and the more serious of these should normally be commented upon in the discussion or experimental section of a paper or in the "special_details" fields of the CIF. checkCIF was carefully designed to identify outliers and unusual parameters, but every test has its limitations and alerts that are not important in a particular case may appear. Conversely, the absence of alerts does not guarantee there are no aspects of the results needing attention. It is up to the individual to critically assess their own results and, if necessary, seek expert advice.

Publication of your CIF in IUCr journals

A basic structural check has been run on your CIF. These basic checks will be run on all CIFs submitted for publication in IUCr journals (*Acta Crystallographica*, *Journal of Applied Crystallography*, *Journal of Synchrotron Radiation*); however, if you intend to submit to *Acta Crystallographica Section C* or *E* or *IUCrData*, you should make sure that full publication checks are run on the final version of your CIF prior to submission.

Publication of your CIF in other journals

Please refer to the *Notes for Authors* of the relevant journal for any special instructions relating to CIF submission.

PLATON version of 13/07/2021; check.def file version of 13/07/2021

Datablock md_4164_beda - ellipsoid plot

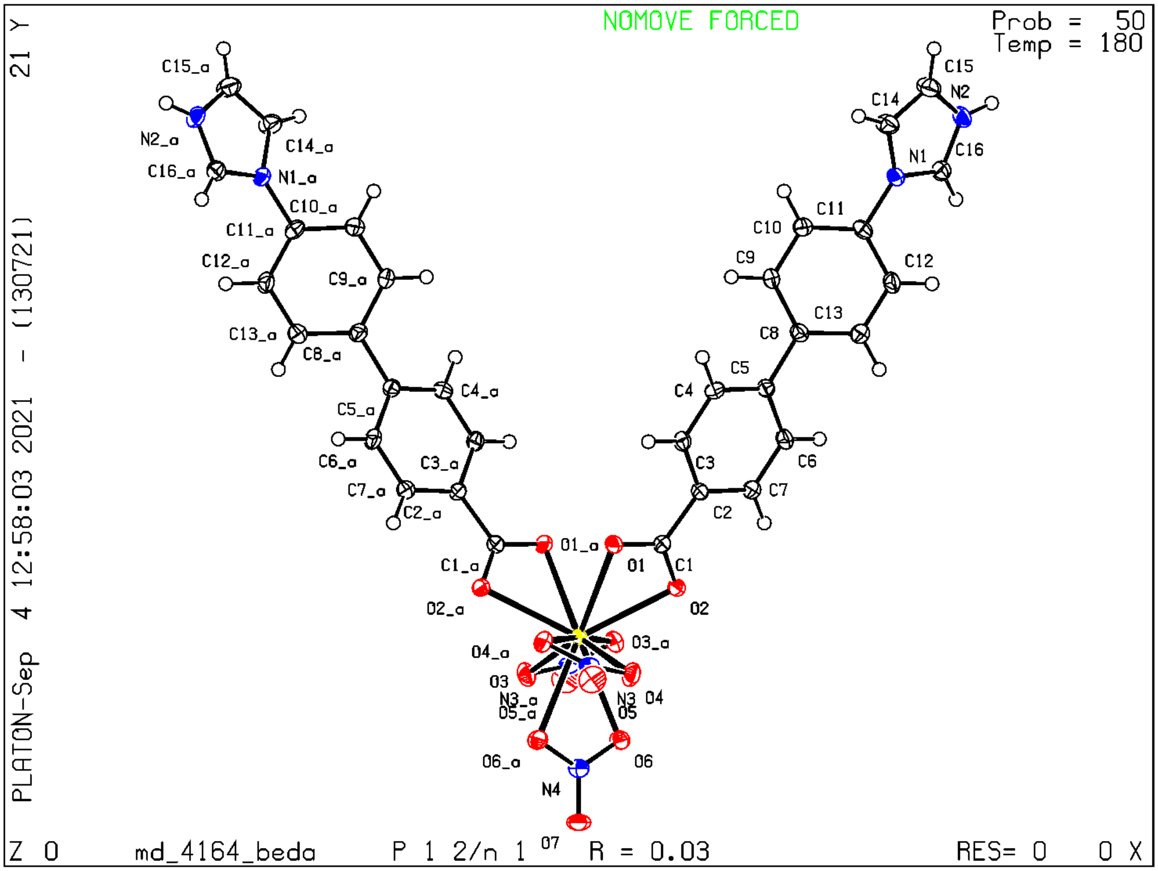

checkCIF/PLATON report

Structure factors have been supplied for datablock(s) shi_4078_beda

THIS REPORT IS FOR GUIDANCE ONLY. IF USED AS PART OF A REVIEW PROCEDURE FOR PUBLICATION, IT SHOULD NOT REPLACE THE EXPERTISE OF AN EXPERIENCED CRYSTALLOGRAPHIC REFEREE.

No syntax errors found. CIF dictionary Interpreting this report

Datablock: shi_4078_beda

Bond precision:	C-C = 0.0058 A		Wavelength=0.71073
Cell:	a=11.6264(8)	b=10.1166(6)	c=14.0056(8)
	alpha=90	beta=109.845(7)	gamma=90
Temperature:	180 K		

	Calculated	Reported
Volume	1549.51(18)	1549.51(17)
Space group	P 2/n	P 1 2/n 1
Hall group	-P 2yac	-P 2yac
Moiety formula	C32 H24 N7 Nd O13	C32 H24 N7 Nd O13
Sum formula	C32 H24 N7 Nd O13	C32 H24 N7 Nd O13
Mr	858.82	858.82
Dx,g cm-3	1.841	1.841
Z	2	2
Mu (mm-1)	1.761	1.761
F000	858.0	858.0
F000'	858.02	
h,k,lmax	13,12,16	13,12,16
Nref	2742	2743
Tmin,Tmax	0.810,0.916	0.969,1.000
Tmin'	0.644	

Correction method= # Reported T Limits: Tmin=0.969 Tmax=1.000
AbsCorr = MULTI-SCAN

Data completeness= 1.000 Theta(max)= 25.026

R(reflections)= 0.0365(2370) wR2(reflections)= 0.0609(2743)

S = 1.007 Npar= 241

The following ALERTS were generated. Each ALERT has the format
test-name_ALERT_alert-type_alert-level.
Click on the hyperlinks for more details of the test.

Alert level C

```
PLAT906_ALERT_3_C Large K Value in the Analysis of Variance ......      2.687 Check
```

Alert level G

```
PLAT007_ALERT_5_G Number of Unrefined Donor-H Atoms .............          1 Report
PLAT432_ALERT_2_G Short Inter X...Y Contact  O5       ..C16             2.90 Ang.
                                                 1+x,1+y,z  =      1_665 Check
PLAT794_ALERT_5_G Tentative Bond Valency for Nd1       (II)      .       2.06 Info
PLAT909_ALERT_3_G Percentage of I>2sig(I) Data at Theta(Max) Still        78% Note
PLAT941_ALERT_3_G Average HKL Measurement Multiplicity ..........         2.1 Low
PLAT978_ALERT_2_G Number C-C Bonds with Positive Residual Density.          2 Info
```

0 **ALERT level A** = Most likely a serious problem - resolve or explain
0 **ALERT level B** = A potentially serious problem, consider carefully
1 **ALERT level C** = Check. Ensure it is not caused by an omission or oversight
6 **ALERT level G** = General information/check it is not something unexpected

0 ALERT type 1 CIF construction/syntax error, inconsistent or missing data
2 ALERT type 2 Indicator that the structure model may be wrong or deficient
3 ALERT type 3 Indicator that the structure quality may be low
0 ALERT type 4 Improvement, methodology, query or suggestion
2 ALERT type 5 Informative message, check

It is advisable to attempt to resolve as many as possible of the alerts in all categories. Often the minor alerts point to easily fixed oversights, errors and omissions in your CIF or refinement strategy, so attention to these fine details can be worthwhile. In order to resolve some of the more serious problems it may be necessary to carry out additional measurements or structure refinements. However, the purpose of your study may justify the reported deviations and the more serious of these should normally be commented upon in the discussion or experimental section of a paper or in the "special_details" fields of the CIF. checkCIF was carefully designed to identify outliers and unusual parameters, but every test has its limitations and alerts that are not important in a particular case may appear. Conversely, the absence of alerts does not guarantee there are no aspects of the results needing attention. It is up to the individual to critically assess their own results and, if necessary, seek expert advice.

Publication of your CIF in IUCr journals

A basic structural check has been run on your CIF. These basic checks will be run on all CIFs submitted for publication in IUCr journals (*Acta Crystallographica*, *Journal of Applied Crystallography*, *Journal of Synchrotron Radiation*); however, if you intend to submit to *Acta Crystallographica Section C* or *E* or *IUCrData*, you should make sure that full publication checks are run on the final version of your CIF prior to submission.

Publication of your CIF in other journals

Please refer to the *Notes for Authors* of the relevant journal for any special instructions relating to CIF submission.

PLATON version of 13/07/2021; check.def file version of 13/07/2021

Datablock shi_4078_beda - ellipsoid plot

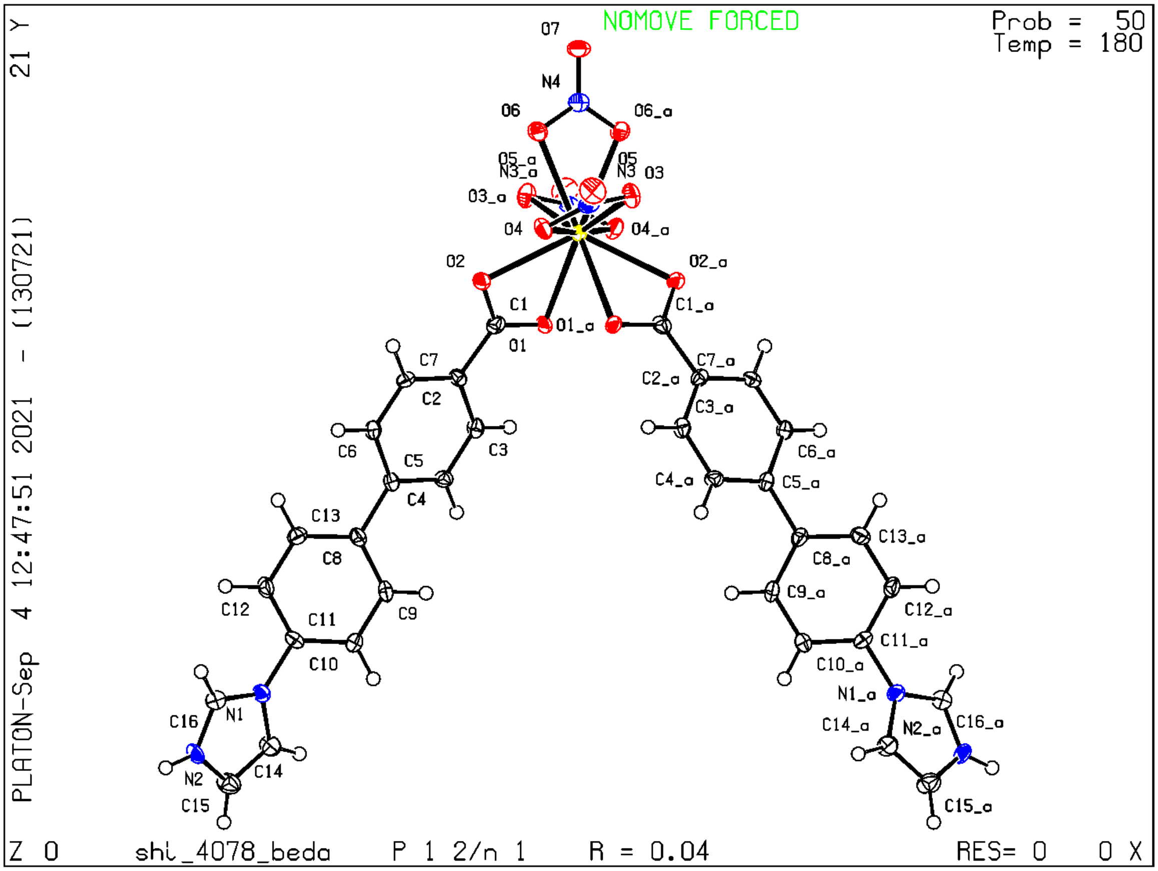

checkCIF/PLATON report

Structure factors have been supplied for datablock(s) md_4167_beda

THIS REPORT IS FOR GUIDANCE ONLY. IF USED AS PART OF A REVIEW PROCEDURE FOR PUBLICATION, IT SHOULD NOT REPLACE THE EXPERTISE OF AN EXPERIENCED CRYSTALLOGRAPHIC REFEREE.

No syntax errors found. CIF dictionary Interpreting this report

Datablock: md_4167_beda

```
Bond precision:   C-C = 0.0056 A          Wavelength=0.71073

Cell:             a=11.6229(7)     b=10.0972(5)        c=14.0020(8)
                  alpha=90         beta=109.798(7)     gamma=90
Temperature:      180 K

                 Calculated                   Reported
Volume           1546.13(16)                  1546.14(16)
Space group      P 2/n                        P 1 2/n 1
Hall group       -P 2yac                      -P 2yac
Moiety formula   C32 H24 Eu N7 O13            C32 H24 Eu N7 O13
Sum formula      C32 H24 Eu N7 O13            C32 H24 Eu N7 O13
Mr               866.55                       866.54
Dx,g cm-3        1.861                        1.861
Z                2                            2
Mu (mm-1)        2.114                        2.114
F000             864.0                        864.0
F000'            864.09
h,k,lmax         15,13,19                     15,13,19
Nref             4162                         3599
Tmin,Tmax        0.881,0.900                  0.981,1.000
Tmin'            0.530

Correction method= # Reported T Limits: Tmin=0.981 Tmax=1.000
AbsCorr = MULTI-SCAN

Data completeness= 0.865           Theta(max)= 29.131

                                                     wR2(reflections)=
R(reflections)= 0.0419( 2991)                        0.0700( 3599)

S = 1.042                    Npar= 241
```

The following ALERTS were generated. Each ALERT has the format
test-name_ALERT_alert-type_alert-level.
Click on the hyperlinks for more details of the test.

Alert level C

```
PLAT906_ALERT_3_C Large K Value in the Analysis of Variance ......      3.980 Check
PLAT973_ALERT_2_C Check Calcd Positive Resid. Density on       Eu1       1.12 eA-3
PLAT975_ALERT_2_C Check Calcd Resid. Dens.  0.87A   From O4              0.43 eA-3
```

Alert level G

```
PLAT007_ALERT_5_G Number of Unrefined Donor-H Atoms .............          1 Report
PLAT432_ALERT_2_G Short Inter X...Y Contact  O5       ..C16              2.89 Ang.
                                        1+x,1+y,z  =      1_665 Check
PLAT779_ALERT_4_G Suspect or Irrelevant (Bond) Angle(s) in CIF ...       0.00 Deg.
              N4   -O6   -N4      2_655   1_555   1_555 ......     #   52 Check
PLAT779_ALERT_4_G Suspect or Irrelevant (Bond) Angle(s) in CIF ...       0.00 Deg.
              N4   -O7   -N4      2_655   1_555   1_555 ......     #   53 Check
PLAT779_ALERT_4_G Suspect or Irrelevant (Bond) Angle(s) in CIF ...       0.00 Deg.
              N4   -N4   -O6      2_655   1_555   1_555 ......     #   66 Check
PLAT779_ALERT_4_G Suspect or Irrelevant (Bond) Angle(s) in CIF ...       0.00 Deg.
              N4   -N4   -O6      2_655   1_555   2_655 ......     #   67 Check
PLAT779_ALERT_4_G Suspect or Irrelevant (Bond) Angle(s) in CIF ...       0.00 Deg.
              N4   -N4   -O7      2_655   1_555   1_555 ......     #   68 Check
PLAT794_ALERT_5_G Tentative Bond Valency for Eu1       (III)     .       3.18 Info
PLAT912_ALERT_4_G Missing # of FCF Reflections Above STh/L=  0.600        562 Note
PLAT941_ALERT_3_G Average HKL Measurement Multiplicity ..........         2.1 Low
PLAT978_ALERT_2_G Number C-C Bonds with Positive Residual Density.          2 Info
```

0 **ALERT level A** = Most likely a serious problem - resolve or explain
0 **ALERT level B** = A potentially serious problem, consider carefully
3 **ALERT level C** = Check. Ensure it is not caused by an omission or oversight
11 **ALERT level G** = General information/check it is not something unexpected

0 ALERT type 1 CIF construction/syntax error, inconsistent or missing data
4 ALERT type 2 Indicator that the structure model may be wrong or deficient
2 ALERT type 3 Indicator that the structure quality may be low
6 ALERT type 4 Improvement, methodology, query or suggestion
2 ALERT type 5 Informative message, check

It is advisable to attempt to resolve as many as possible of the alerts in all categories. Often the minor alerts point to easily fixed oversights, errors and omissions in your CIF or refinement strategy, so attention to these fine details can be worthwhile. In order to resolve some of the more serious problems it may be necessary to carry out additional measurements or structure refinements. However, the purpose of your study may justify the reported deviations and the more serious of these should normally be commented upon in the discussion or experimental section of a paper or in the "special_details" fields of the CIF. checkCIF was carefully designed to identify outliers and unusual parameters, but every test has its limitations and alerts that are not important in a particular case may appear. Conversely, the absence of alerts does not guarantee there are no aspects of the results needing attention. It is up to the individual to critically assess their own results and, if necessary, seek expert advice.

Publication of your CIF in IUCr journals

A basic structural check has been run on your CIF. These basic checks will be run on all CIFs submitted for publication in IUCr journals (*Acta Crystallographica*, *Journal of Applied Crystallography*, *Journal of Synchrotron Radiation*); however, if you intend to submit to *Acta Crystallographica Section C* or *E* or *IUCrData*, you should make sure that full publication checks are run on the final version of your CIF prior to submission.

Publication of your CIF in other journals

Please refer to the *Notes for Authors* of the relevant journal for any special instructions relating to CIF submission.

PLATON version of 13/07/2021; check.def file version of 13/07/2021

Datablock md_4167_beda - ellipsoid plot

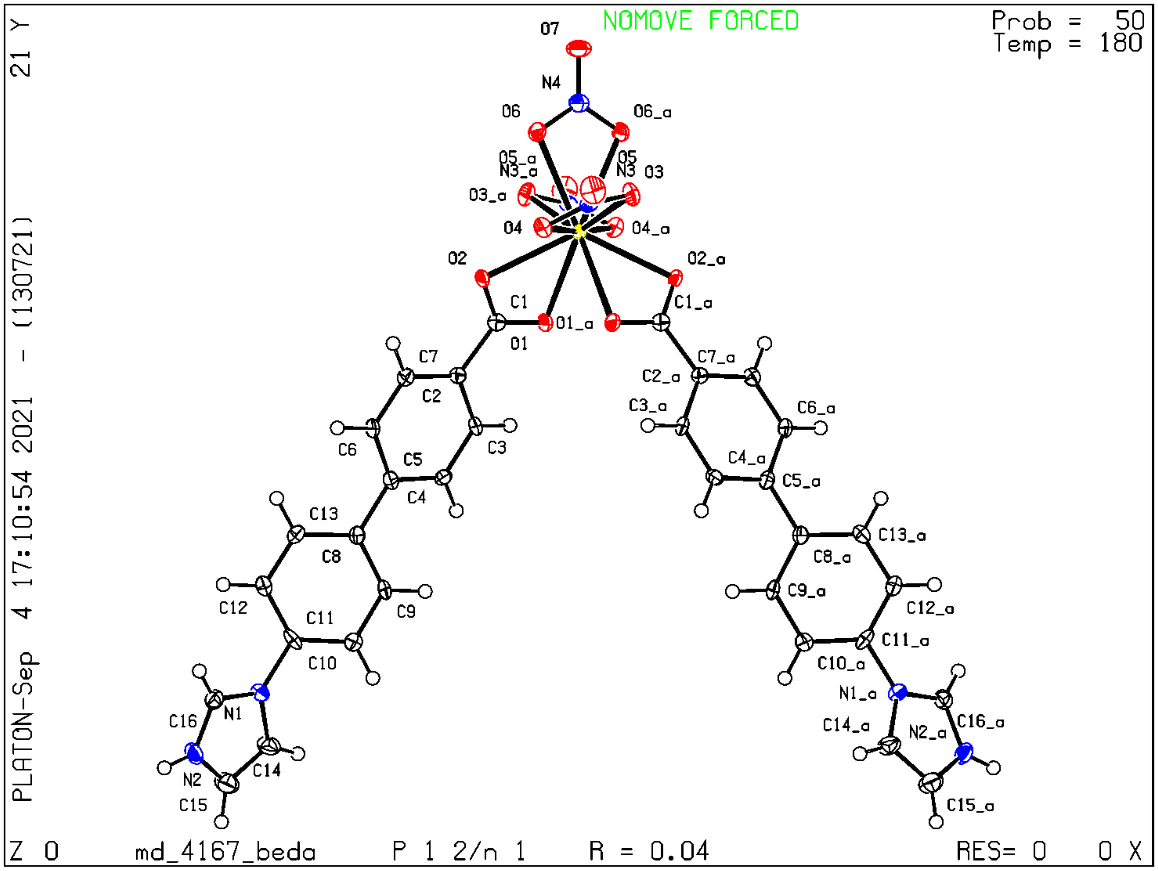

checkCIF/PLATON report

Structure factors have been supplied for datablock(s) md_4168_beda

THIS REPORT IS FOR GUIDANCE ONLY. IF USED AS PART OF A REVIEW PROCEDURE FOR PUBLICATION, IT SHOULD NOT REPLACE THE EXPERTISE OF AN EXPERIENCED CRYSTALLOGRAPHIC REFEREE.

No syntax errors found. CIF dictionary Interpreting this report

Datablock: md_4168_beda

```
Bond precision:   C-C = 0.0043 A          Wavelength=0.71073

Cell:             a=11.6154(7)     b=10.0797(5)        c=13.9905(7)
                  alpha=90         beta=109.817(7)     gamma=90
Temperature:      180 K

                 Calculated                    Reported
Volume           1541.00(16)                   1541.00(16)
Space group      P 2/n                         P 1 2/n 1
Hall group       -P 2yac                       -P 2yac
Moiety formula   C32 H24 Gd N7 O13             C32 H24 Gd N7 O13
Sum formula      C32 H24 Gd N7 O13             C32 H24 Gd N7 O13
Mr               871.83                        871.83
Dx,g cm-3        1.879                         1.879
Z                2                             2
Mu (mm-1)        2.238                         2.238
F000             866.0                         866.0
F000'            866.07
h,k,lmax         13,11,16                      13,11,16
Nref             2726                          2712
Tmin,Tmax        0.874,0.894                   0.900,1.000
Tmin'            0.511

Correction method= # Reported T Limits: Tmin=0.900 Tmax=1.000
AbsCorr = MULTI-SCAN

Data completeness= 0.995           Theta(max)= 25.024

                                                        wR2(reflections)=
R(reflections)= 0.0300( 2377)                           0.0476( 2712)

S = 1.057                    Npar= 241
```

The following ALERTS were generated. Each ALERT has the format

test-name_ALERT_alert-type_alert-level.

Click on the hyperlinks for more details of the test.

Alert level C

```
PLAT230_ALERT_2_C Hirshfeld Test Diff for    O1       --C1       .        5.5 s.u.
PLAT230_ALERT_2_C Hirshfeld Test Diff for    O3       --N3       .        5.5 s.u.
PLAT230_ALERT_2_C Hirshfeld Test Diff for    O6       --N4       .        5.5 s.u.
PLAT232_ALERT_2_C Hirshfeld Test Diff (M-X)  Gd1      --O4       .        6.4 s.u.
PLAT232_ALERT_2_C Hirshfeld Test Diff (M-X)  Gd1      --O6       .        6.5 s.u.
PLAT906_ALERT_3_C Large K Value in the Analysis of Variance ......      3.080 Check
PLAT911_ALERT_3_C Missing FCF Refl Between Thmin & STh/L=    0.595         15 Report
```

Alert level G

```
PLAT007_ALERT_5_G Number of Unrefined Donor-H Atoms .............           1 Report
PLAT432_ALERT_2_G Short Inter X...Y Contact  O5       ..C16              2.88 Ang.
                                         1+x,1+y,z  =      1_665 Check
PLAT794_ALERT_5_G Tentative Bond Valency for Gd1       (III)     .       3.21 Info
PLAT883_ALERT_1_G No Info/Value for _atom_sites_solution_primary .     Please Do !
PLAT909_ALERT_3_G Percentage of I>2sig(I) Data at Theta(Max) Still        81% Note
PLAT933_ALERT_2_G Number of OMIT Records in Embedded .res File ...          2 Note
PLAT941_ALERT_3_G Average HKL Measurement Multiplicity ..........         2.2 Low
PLAT978_ALERT_2_G Number C-C Bonds with Positive Residual Density.          1 Info
```

0 **ALERT level A** = Most likely a serious problem - resolve or explain
0 **ALERT level B** = A potentially serious problem, consider carefully
7 **ALERT level C** = Check. Ensure it is not caused by an omission or oversight
8 **ALERT level G** = General information/check it is not something unexpected

1 ALERT type 1 CIF construction/syntax error, inconsistent or missing data
8 ALERT type 2 Indicator that the structure model may be wrong or deficient
4 ALERT type 3 Indicator that the structure quality may be low
0 ALERT type 4 Improvement, methodology, query or suggestion
2 ALERT type 5 Informative message, check

It is advisable to attempt to resolve as many as possible of the alerts in all categories. Often the minor alerts point to easily fixed oversights, errors and omissions in your CIF or refinement strategy, so attention to these fine details can be worthwhile. In order to resolve some of the more serious problems it may be necessary to carry out additional measurements or structure refinements. However, the purpose of your study may justify the reported deviations and the more serious of these should normally be commented upon in the discussion or experimental section of a paper or in the "special_details" fields of the CIF. checkCIF was carefully designed to identify outliers and unusual parameters, but every test has its limitations and alerts that are not important in a particular case may appear. Conversely, the absence of alerts does not guarantee there are no aspects of the results needing attention. It is up to the individual to critically assess their own results and, if necessary, seek expert advice.

Publication of your CIF in IUCr journals

A basic structural check has been run on your CIF. These basic checks will be run on all CIFs submitted for publication in IUCr journals (*Acta Crystallographica*, *Journal of Applied Crystallography*, *Journal of Synchrotron Radiation*); however, if you intend to submit to *Acta Crystallographica Section C* or *E* or *IUCrData*, you should make sure that full publication checks are run on the final version of your CIF prior to submission.

Publication of your CIF in other journals

Please refer to the *Notes for Authors* of the relevant journal for any special instructions relating to CIF submission.

PLATON version of 13/07/2021; check.def file version of 13/07/2021

Datablock md_4168_beda - ellipsoid plot

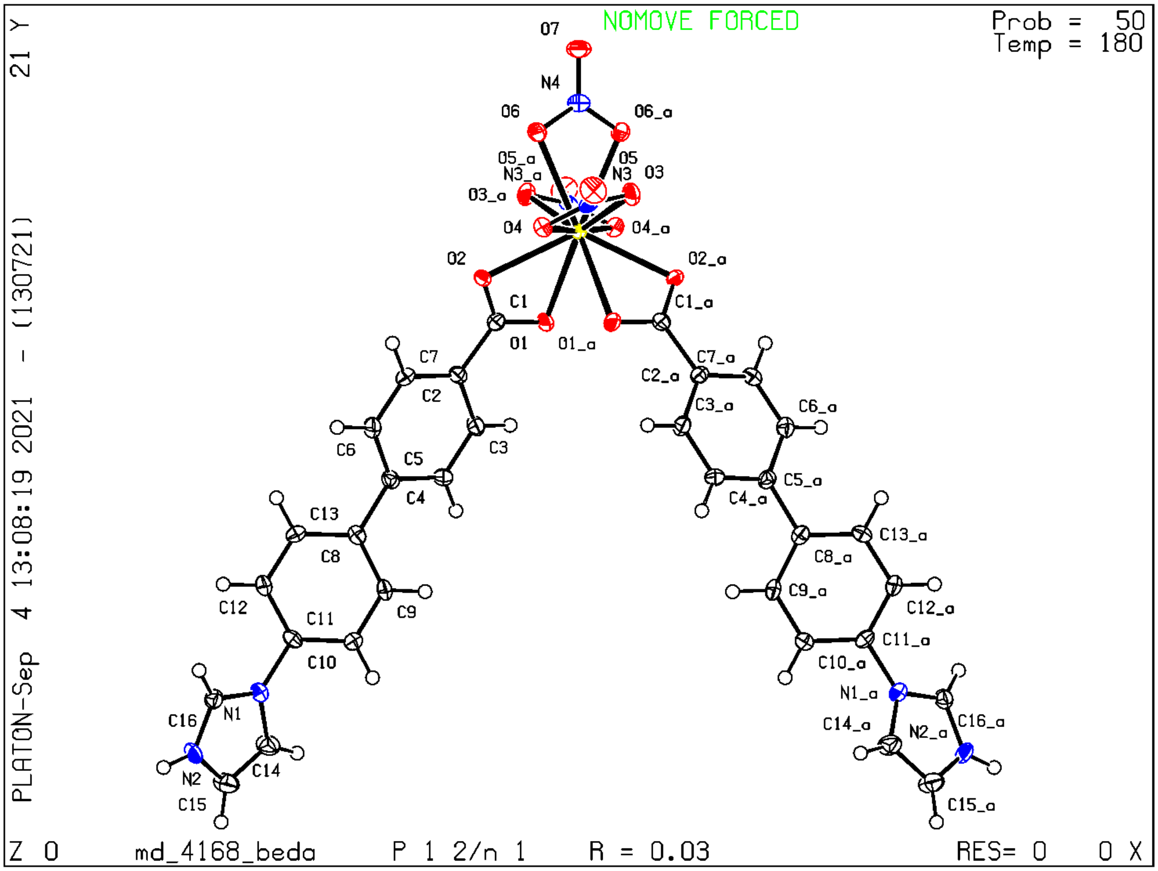

checkCIF/PLATON report

Structure factors have been supplied for datablock(s) shi_4108_beda

THIS REPORT IS FOR GUIDANCE ONLY. IF USED AS PART OF A REVIEW PROCEDURE FOR PUBLICATION, IT SHOULD NOT REPLACE THE EXPERTISE OF AN EXPERIENCED CRYSTALLOGRAPHIC REFEREE.

No syntax errors found. CIF dictionary Interpreting this report

Datablock: shi_4108_beda

```
Bond precision:   C-C = 0.0057 A          Wavelength=0.71073

Cell:             a=11.5987(4)     b=10.0803(3)        c=13.9615(5)
                  alpha=90         beta=109.871(4)     gamma=90
Temperature:      180 K

                 Calculated                    Reported
Volume           1535.17(10)                   1535.17(10)
Space group      P 2/n                         P 1 2/n 1
Hall group       -P 2yac                       -P 2yac
Moiety formula   C32 H24 Dy N7 O13             C32 H24 Dy N7 O13
Sum formula      C32 H24 Dy N7 O13             C32 H24 Dy N7 O13
Mr               877.08                        877.08
Dx,g cm-3        1.897                         1.897
Z                2                             2
Mu (mm-1)        2.520                         2.520
F000             870.0                         870.0
F000'            870.03
h,k,lmax         13,12,16                      13,12,16
Nref             2715                          2713
Tmin,Tmax        0.860,0.882                   0.710,1.000
Tmin'            0.777

Correction method= # Reported T Limits: Tmin=0.710 Tmax=1.000
AbsCorr = MULTI-SCAN

Data completeness= 0.999           Theta(max)= 25.027

R(reflections)= 0.0301( 2470)                         wR2(reflections)=
                                                      0.0622( 2713)

S = 1.070                   Npar= 241
```

The following ALERTS were generated. Each ALERT has the format
test-name_ALERT_alert-type_alert-level.
Click on the hyperlinks for more details of the test.

Alert level C

```
PLAT232_ALERT_2_C Hirshfeld Test Diff (M-X)  Dy1      --O6       .        5.2 s.u.
PLAT906_ALERT_3_C Large K Value in the Analysis of Variance ......      3.402 Check
PLAT911_ALERT_3_C Missing FCF Refl Between Thmin & STh/L=    0.595          2 Report
```

Alert level G

```
PLAT007_ALERT_5_G Number of Unrefined Donor-H Atoms .............          1 Report
PLAT432_ALERT_2_G Short Inter X...Y Contact  O5       ..C16             2.88 Ang.
                                           3/2-x,-1+y,3/2-z  =      2_646 Check
PLAT794_ALERT_5_G Tentative Bond Valency for Dy1       (III)     .       2.87 Info
PLAT909_ALERT_3_G Percentage of I>2sig(I) Data at Theta(Max) Still        86% Note
PLAT933_ALERT_2_G Number of OMIT Records in Embedded .res File ...          2 Note
PLAT941_ALERT_3_G Average HKL Measurement Multiplicity ..........         4.1 Low
PLAT978_ALERT_2_G Number C-C Bonds with Positive Residual Density.          3 Info
```

0 **ALERT level A** = Most likely a serious problem - resolve or explain
0 **ALERT level B** = A potentially serious problem, consider carefully
3 **ALERT level C** = Check. Ensure it is not caused by an omission or oversight
7 **ALERT level G** = General information/check it is not something unexpected

0 ALERT type 1 CIF construction/syntax error, inconsistent or missing data
4 ALERT type 2 Indicator that the structure model may be wrong or deficient
4 ALERT type 3 Indicator that the structure quality may be low
0 ALERT type 4 Improvement, methodology, query or suggestion
2 ALERT type 5 Informative message, check

It is advisable to attempt to resolve as many as possible of the alerts in all categories. Often the minor alerts point to easily fixed oversights, errors and omissions in your CIF or refinement strategy, so attention to these fine details can be worthwhile. In order to resolve some of the more serious problems it may be necessary to carry out additional measurements or structure refinements. However, the purpose of your study may justify the reported deviations and the more serious of these should normally be commented upon in the discussion or experimental section of a paper or in the "special_details" fields of the CIF. checkCIF was carefully designed to identify outliers and unusual parameters, but every test has its limitations and alerts that are not important in a particular case may appear. Conversely, the absence of alerts does not guarantee there are no aspects of the results needing attention. It is up to the individual to critically assess their own results and, if necessary, seek expert advice.

Publication of your CIF in IUCr journals

A basic structural check has been run on your CIF. These basic checks will be run on all CIFs submitted for publication in IUCr journals (*Acta Crystallographica*, *Journal of Applied Crystallography*, *Journal of Synchrotron Radiation*); however, if you intend to submit to *Acta Crystallographica Section C* or *E* or *IUCrData*, you should make sure that full publication checks are run on the final version of your CIF prior to submission.

Publication of your CIF in other journals

Please refer to the *Notes for Authors* of the relevant journal for any special instructions relating to CIF submission.

PLATON version of 13/07/2021; check.def file version of 13/07/2021

Datablock shi_4108_beda - ellipsoid plot

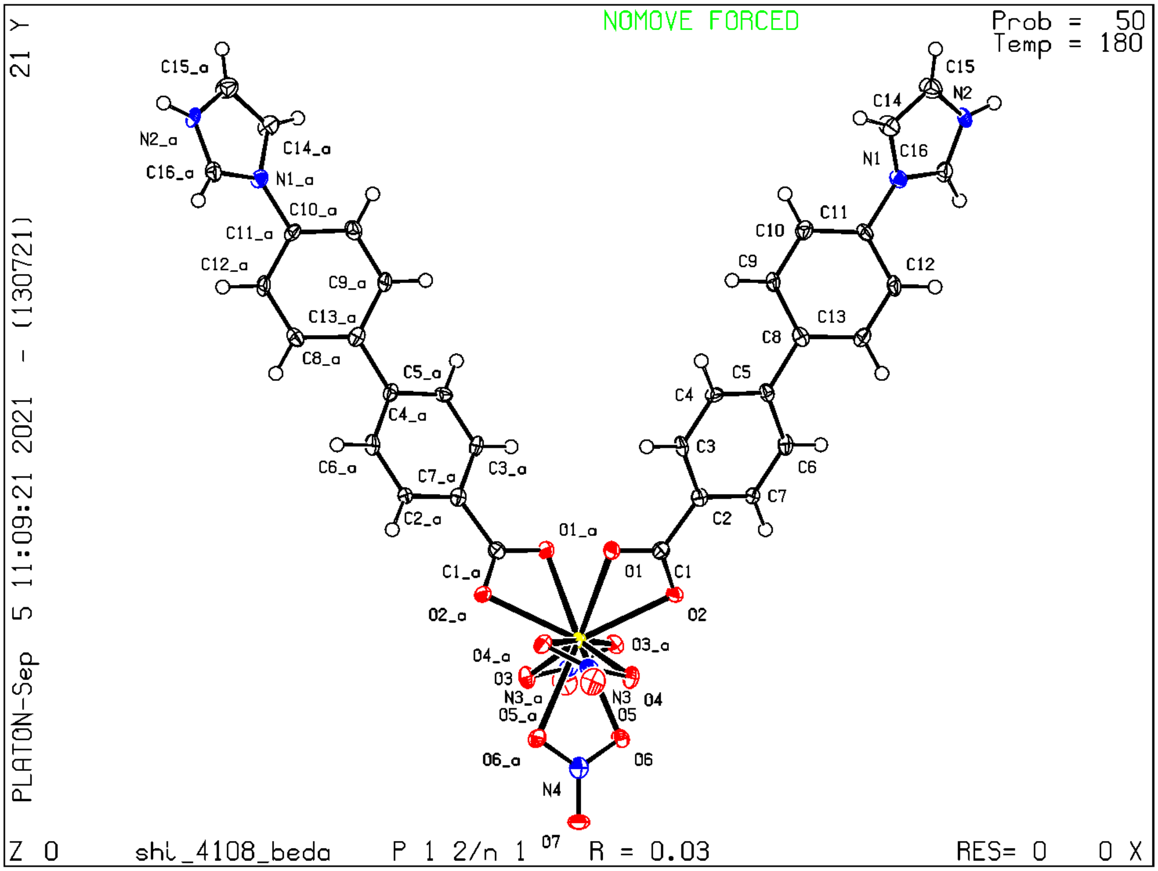

checkCIF/PLATON report

Structure factors have been supplied for datablock(s) shi_4151_beda

THIS REPORT IS FOR GUIDANCE ONLY. IF USED AS PART OF A REVIEW PROCEDURE FOR PUBLICATION, IT SHOULD NOT REPLACE THE EXPERTISE OF AN EXPERIENCED CRYSTALLOGRAPHIC REFEREE.

No syntax errors found. CIF dictionary Interpreting this report

Datablock: shi_4151_beda

```
Bond precision:   C-C = 0.0061 A          Wavelength=0.71073

Cell:            a=11.6138(3)     b=10.0845(2)       c=14.0087(4)
                 alpha=90         beta=109.726(3)    gamma=90
Temperature:     180 K

                Calculated                  Reported
Volume          1544.41(7)                  1544.41(7)
Space group     P 2/n                       P 1 2/n 1
Hall group      -P 2yac                     -P 2yac
Moiety formula  C32 H24 Ho N7 O13           C32 H24 Ho N7 O13
Sum formula     C32 H24 Ho N7 O13           C32 H24 Ho N7 O13
Mr              879.51                      879.51
Dx,g cm-3       1.891                       1.891
Z               2                           2
Mu (mm-1)       2.647                       2.647
F000            872.0                       872.0
F000'           871.97
h,k,lmax        13,12,16                    13,12,16
Nref            2732                        2727
Tmin,Tmax       0.853,0.876                 0.929,1.000
Tmin'           0.516

Correction method= # Reported T Limits: Tmin=0.929 Tmax=1.000
AbsCorr = MULTI-SCAN

Data completeness= 0.998          Theta(max)= 25.022

R(reflections)= 0.0304( 2421)                 wR2(reflections)=
                                              0.0764( 2727)
S = 1.040                  Npar= 241
```

The following ALERTS were generated. Each ALERT has the format
test-name_ALERT_alert-type_alert-level.
Click on the hyperlinks for more details of the test.

Alert level C

```
PLAT911_ALERT_3_C Missing FCF Refl Between Thmin & STh/L=    0.595          5 Report
PLAT975_ALERT_2_C Check Calcd Resid. Dens.  0.80A   From O2              0.46 eA-3
```

Alert level G

```
PLAT007_ALERT_5_G Number of Unrefined Donor-H Atoms .............           1 Report
PLAT432_ALERT_2_G Short Inter X...Y Contact  O5       ..C16              2.88 Ang.
                                                  1+x,1+y,z  =      1_665 Check
PLAT794_ALERT_5_G Tentative Bond Valency for Ho1       (III)     .       2.80 Info
PLAT909_ALERT_3_G Percentage of I>2sig(I) Data at Theta(Max) Still        80% Note
PLAT933_ALERT_2_G Number of OMIT Records in Embedded .res File ...          5 Note
PLAT978_ALERT_2_G Number C-C Bonds with Positive Residual Density.          5 Info
```

0 **ALERT level A** = Most likely a serious problem - resolve or explain
0 **ALERT level B** = A potentially serious problem, consider carefully
2 **ALERT level C** = Check. Ensure it is not caused by an omission or oversight
6 **ALERT level G** = General information/check it is not something unexpected

0 ALERT type 1 CIF construction/syntax error, inconsistent or missing data
4 ALERT type 2 Indicator that the structure model may be wrong or deficient
2 ALERT type 3 Indicator that the structure quality may be low
0 ALERT type 4 Improvement, methodology, query or suggestion
2 ALERT type 5 Informative message, check

It is advisable to attempt to resolve as many as possible of the alerts in all categories. Often the minor alerts point to easily fixed oversights, errors and omissions in your CIF or refinement strategy, so attention to these fine details can be worthwhile. In order to resolve some of the more serious problems it may be necessary to carry out additional measurements or structure refinements. However, the purpose of your study may justify the reported deviations and the more serious of these should normally be commented upon in the discussion or experimental section of a paper or in the "special_details" fields of the CIF. checkCIF was carefully designed to identify outliers and unusual parameters, but every test has its limitations and alerts that are not important in a particular case may appear. Conversely, the absence of alerts does not guarantee there are no aspects of the results needing attention. It is up to the individual to critically assess their own results and, if necessary, seek expert advice.

Publication of your CIF in IUCr journals

A basic structural check has been run on your CIF. These basic checks will be run on all CIFs submitted for publication in IUCr journals (*Acta Crystallographica*, *Journal of Applied Crystallography*, *Journal of Synchrotron Radiation*); however, if you intend to submit to *Acta Crystallographica Section C* or *E* or *IUCrData*, you should make sure that full publication checks are run on the final version of your CIF prior to submission.

Publication of your CIF in other journals

Please refer to the *Notes for Authors* of the relevant journal for any special instructions relating to CIF submission.

PLATON version of 13/07/2021; check.def file version of 13/07/2021

Datablock shi_4151_beda - ellipsoid plot

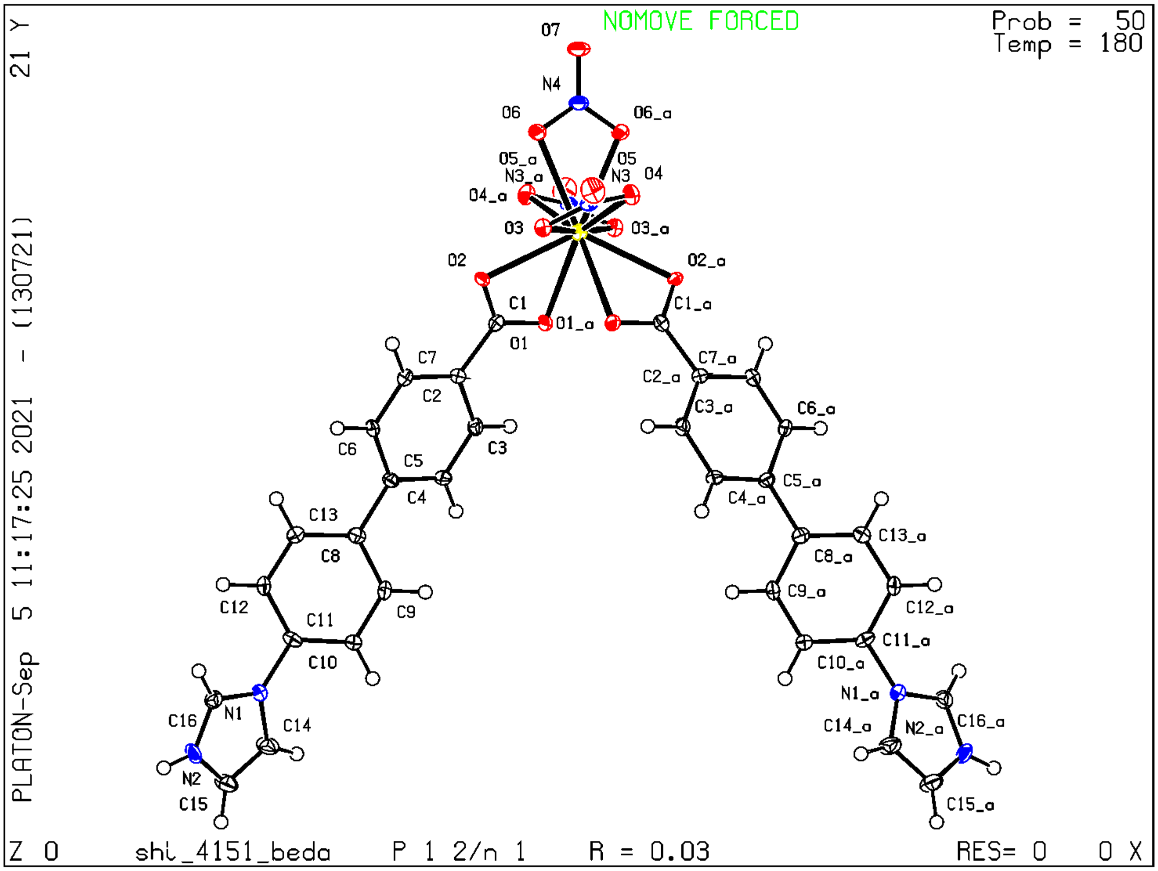

Supplement: Supplementary file 1 [file molecules-26-06942-s001.zip › CRystallografic data/CIFreport_Ln1.pdf]
