# Supplementary material for: Novel Lanthanide (III) Complexes Derived from an Imidazole–Biphenyl–Carboxylate Ligand: Synthesis, Structure and Luminescence Properties
Source: Molecules. 2021 Nov 17;26(22):6942. doi: 10.3390/molecules26226942 (PMC8625298; doi:10.3390/molecules26226942)

checkCIF/PLATON report

Structure factors have been supplied for datablock(s) md_4164_beda

THIS REPORT IS FOR GUIDANCE ONLY. IF USED AS PART OF A REVIEW PROCEDURE FOR PUBLICATION, IT SHOULD NOT REPLACE THE EXPERTISE OF AN EXPERIENCED CRYSTALLOGRAPHIC REFEREE.

No syntax errors found. CIF dictionary Interpreting this report

Datablock: md_4164_beda

Bond precision: C-C = 0.0041 A Wavelength=0.71073

Cell:	a=11.6491(5)	b=10.1346(4)	c=14.0436(7)
	alpha=90	beta=109.849(5)	gamma=90
Temperature:	180 K		

	Calculated	Reported
Volume	1559.47(13)	1559.48(13)
Space group	P 2/n	P 1 2/n 1
Hall group	-P 2yac	-P 2yac
Moiety formula	C32 H24 Ce N7 O13	C32 H24 Ce N7 O13
Sum formula	C32 H24 Ce N7 O13	C32 H24 Ce N7 O13
Mr	854.70	854.70
Dx,g cm-3	1.820	1.820
Z	2	2
Mu (mm-1)	1.544	1.544
F000	854.0	854.0
F000’	853.91	
h,k,lmax	16,13,19	15,13,18
Nref	4291	3657
Tmin,Tmax	0.831,0.926	0.759,1.000
Tmin’	0.734	

Correction method= # Reported T Limits: Tmin=0.759 Tmax=1.000
AbsCorr = MULTI-SCAN

Data completeness= 0.852 Theta(max)= 29.356

R(reflections)= 0.0341(3232) wR2(reflections)= 0.0668(3657)

S = 1.049 Npar= 241

The following ALERTS were generated. Each ALERT has the format
test-name_ALERT_alert-type_alert-level.
Click on the hyperlinks for more details of the test.

Alert level C

```
PLAT906_ALERT_3_C Large K Value in the Analysis of Variance ......      2.257 Check
```

Alert level G

```
PLAT007_ALERT_5_G Number of Unrefined Donor-H Atoms .............          1 Report
PLAT232_ALERT_2_G Hirshfeld Test Diff (M-X)  Ce1      --O3       .        5.5 s.u.
PLAT232_ALERT_2_G Hirshfeld Test Diff (M-X)  Ce1      --O4       .        5.8 s.u.
PLAT232_ALERT_2_G Hirshfeld Test Diff (M-X)  Ce1      --O6       .        6.6 s.u.
PLAT432_ALERT_2_G Short Inter X...Y Contact  O5       ..C16              2.90 Ang.
                                    3/2-x,-1+y,3/2-z  =      2_646 Check
PLAT779_ALERT_4_G Suspect or Irrelevant (Bond) Angle(s) in CIF ...       0.00 Deg.
              N4   -O6   -N4      2_556   1_555   1_555 ......     #   52 Check
PLAT779_ALERT_4_G Suspect or Irrelevant (Bond) Angle(s) in CIF ...       0.00 Deg.
              N4   -O7   -N4      2_556   1_555   1_555 ......     #   53 Check
PLAT779_ALERT_4_G Suspect or Irrelevant (Bond) Angle(s) in CIF ...       0.00 Deg.
              N4   -N4   -O6      2_556   1_555   2_556 ......     #   66 Check
PLAT779_ALERT_4_G Suspect or Irrelevant (Bond) Angle(s) in CIF ...       0.00 Deg.
              N4   -N4   -O6      2_556   1_555   1_555 ......     #   67 Check
PLAT779_ALERT_4_G Suspect or Irrelevant (Bond) Angle(s) in CIF ...       0.00 Deg.
              N4   -N4   -O7      2_556   1_555   1_555 ......     #   68 Check
PLAT794_ALERT_5_G Tentative Bond Valency for Ce1       (III)     .       3.22 Info
PLAT912_ALERT_4_G Missing # of FCF Reflections Above STh/L=  0.600        617 Note
PLAT941_ALERT_3_G Average HKL Measurement Multiplicity ..........        2.1 Low
PLAT978_ALERT_2_G Number C-C Bonds with Positive Residual Density.          6 Info
```

0 **ALERT level A** = Most likely a serious problem - resolve or explain
0 **ALERT level B** = A potentially serious problem, consider carefully
1 **ALERT level C** = Check. Ensure it is not caused by an omission or oversight
14 **ALERT level G** = General information/check it is not something unexpected

0 ALERT type 1 CIF construction/syntax error, inconsistent or missing data
5 ALERT type 2 Indicator that the structure model may be wrong or deficient
2 ALERT type 3 Indicator that the structure quality may be low
6 ALERT type 4 Improvement, methodology, query or suggestion
2 ALERT type 5 Informative message, check

It is advisable to attempt to resolve as many as possible of the alerts in all categories. Often the minor alerts point to easily fixed oversights, errors and omissions in your CIF or refinement strategy, so attention to these fine details can be worthwhile. In order to resolve some of the more serious problems it may be necessary to carry out additional measurements or structure refinements. However, the purpose of your study may justify the reported deviations and the more serious of these should normally be commented upon in the discussion or experimental section of a paper or in the "special_details" fields of the CIF. checkCIF was carefully designed to identify outliers and unusual parameters, but every test has its limitations and alerts that are not important in a particular case may appear. Conversely, the absence of alerts does not guarantee there are no aspects of the results needing attention. It is up to the individual to critically assess their own results and, if necessary, seek expert advice.

Publication of your CIF in IUCr journals

A basic structural check has been run on your CIF. These basic checks will be run on all CIFs submitted for publication in IUCr journals (*Acta Crystallographica*, *Journal of Applied Crystallography*, *Journal of Synchrotron Radiation*); however, if you intend to submit to *Acta Crystallographica Section C* or *E* or *IUCrData*, you should make sure that full publication checks are run on the final version of your CIF prior to submission.

Publication of your CIF in other journals

Please refer to the *Notes for Authors* of the relevant journal for any special instructions relating to CIF submission.

PLATON version of 13/07/2021; check.def file version of 13/07/2021

Datablock md_4164_beda - ellipsoid plot

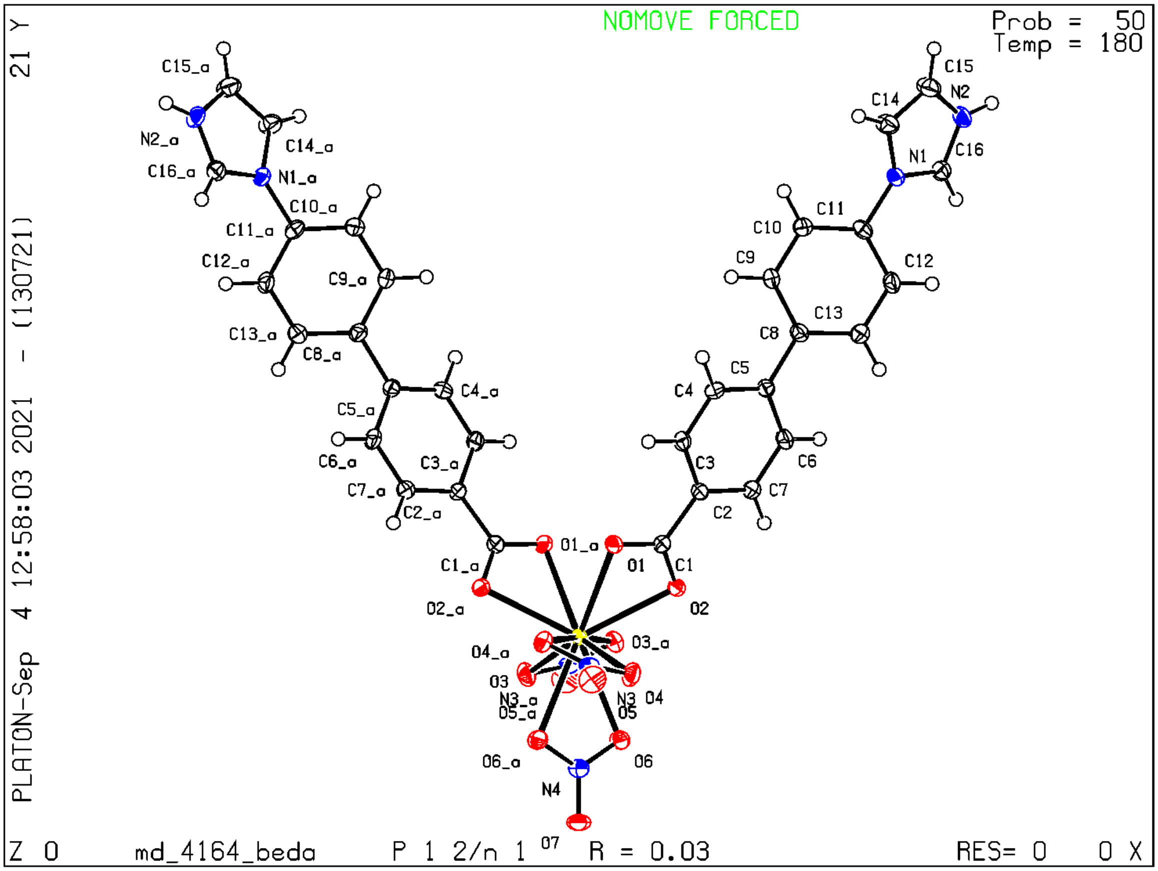

Supplement: Supplementary file 1 [file molecules-26-06942-s001.zip › CRystallografic data/MD_4164_BeDa_cifreport.pdf]
