# Supplementary material for: Novel Lanthanide (III) Complexes Derived from an Imidazole–Biphenyl–Carboxylate Ligand: Synthesis, Structure and Luminescence Properties
Source: Molecules. 2021 Nov 17;26(22):6942. doi: 10.3390/molecules26226942 (PMC8625298; doi:10.3390/molecules26226942)
Supplement: Supplementary file 1 [file molecules-26-06942-s001.zip › CRystallografic data/MD_4164_BeDa_tables.html]

MD\_4164\_BeDa


# MD\_4164\_BeDa

Table 1 Crystal data and structure refinement for MD\_4164\_BeDa.

| Identification code | MD\_4164\_BeDa |
| Empirical formula | C32H24CeN7O13 |
| Formula weight | 854.70 |
| Temperature/K | 180.00(14) |
| Crystal system | monoclinic |
| Space group | P2/n |
| a/Å | 11.6491(5) |
| b/Å | 10.1346(4) |
| c/Å | 14.0436(7) |
| α/° | 90 |
| β/° | 109.849(5) |
| γ/° | 90 |
| Volume/Å3 | 1559.48(13) |
| Z | 2 |
| ρcalcg/cm3 | 1.820 |
| μ/mm‑1 | 1.544 |
| F(000) | 854.0 |
| Crystal size/mm3 | 0.2 × 0.1 × 0.05 |
| Radiation | Mo Kα (λ = 0.71073) |
| 2Θ range for data collection/° | 3.942 to 58.712 |
| Index ranges | -13 ≤ h ≤ 15, -13 ≤ k ≤ 11, -18 ≤ l ≤ 18 |
| Reflections collected | 7773 |
| Independent reflections | 3657 [Rint = 0.0356, Rsigma = 0.0525] |
| Data/restraints/parameters | 3657/0/241 |
| Goodness-of-fit on F2 | 1.050 |
| Final R indexes [I>=2σ (I)] | R1 = 0.0341, wR2 = 0.0639 |
| Final R indexes [all data] | R1 = 0.0419, wR2 = 0.0668 |
| Largest diff. peak/hole / e Å-3 | 0.63/-0.50 |

Table 2 Fractional Atomic Coordinates (×104) and Equivalent Isotropic Displacement Parameters (Å2×103) for MD\_4164\_BeDa. Ueq is defined as 1/3 of of the trace of the orthogonalised UIJ tensor.

| Atom | *x* | *y* | *z* | U(eq) |
| --- | --- | --- | --- | --- |
| Ce1 | 2500 | 2403.9(2) | 7500 | 16.28(7) |
| O1 | 3791.8(18) | 4499.7(18) | 8148.5(15) | 22.1(4) |
| O2 | 3968.1(18) | 3522.2(18) | 6802.4(15) | 22.9(4) |
| O3 | 3413.4(18) | 2295.3(18) | 9453.7(15) | 23.5(4) |
| O4 | 4599.4(18) | 1555.5(19) | 8666.6(15) | 25.9(5) |
| O5 | 5179(2) | 1442(2) | 10312.1(16) | 32.9(5) |
| O6 | 2818.4(18) | 78.7(18) | 6857.1(14) | 23.9(5) |
| O7 | 2500 | -1785(3) | 7500 | 26.2(7) |
| N1 | 7436(2) | 12804(2) | 5446.0(17) | 18.0(5) |
| N2 | 8060(2) | 14154(2) | 4539.4(18) | 23.0(5) |
| N3 | 4420(2) | 1760(2) | 9500.5(18) | 21.4(5) |
| N4 | 2500 | -566(3) | 7500 | 20.0(7) |
| C1 | 4164(2) | 4501(3) | 7394(2) | 18.6(6) |
| C2 | 4776(2) | 5691(3) | 7176(2) | 17.0(6) |
| C3 | 4903(2) | 6829(3) | 7758(2) | 18.7(6) |
| C4 | 5364(2) | 7977(3) | 7496(2) | 19.4(6) |
| C5 | 5714(2) | 8027(3) | 6639(2) | 17.2(6) |
| C6 | 5621(3) | 6876(3) | 6069(2) | 20.9(6) |
| C7 | 5147(3) | 5731(3) | 6325(2) | 20.8(6) |
| C8 | 6164(2) | 9280(3) | 6339(2) | 18.7(6) |
| C9 | 5770(3) | 10505(3) | 6570(2) | 21.8(6) |
| C10 | 6186(3) | 11669(3) | 6279(2) | 22.5(6) |
| C11 | 7013(3) | 11609(3) | 5767(2) | 18.3(6) |
| C12 | 7427(3) | 10411(3) | 5536(2) | 21.7(6) |
| C13 | 6994(3) | 9256(3) | 5812(2) | 21.8(6) |
| C14 | 7701(3) | 13991(3) | 5966(2) | 25.7(7) |
| C15 | 8085(3) | 14827(3) | 5388(2) | 27.9(7) |
| C16 | 7657(3) | 12939(3) | 4578(2) | 21.6(6) |

Table 3 Anisotropic Displacement Parameters (Å2×103) for MD\_4164\_BeDa. The Anisotropic displacement factor exponent takes the form: -2π2[h2a\*2U11+2hka\*b\*U12+…].

| Atom | U11 | U22 | U33 | U23 | U13 | U12 |
| --- | --- | --- | --- | --- | --- | --- |
| Ce1 | 20.48(12) | 12.48(12) | 19.89(12) | 0 | 12.07(9) | 0 |
| O1 | 31.6(12) | 16.9(10) | 25.7(11) | -2.3(8) | 19.9(10) | -3.4(8) |
| O2 | 31.8(12) | 16.1(10) | 29.0(11) | -4.8(8) | 21.0(10) | -4.6(9) |
| O3 | 25.5(11) | 23.6(11) | 25.7(11) | -1.9(8) | 14.2(9) | 2.7(9) |
| O4 | 28.6(12) | 30.4(12) | 24.2(11) | -0.3(9) | 16.1(10) | 4.9(9) |
| O5 | 33.8(13) | 37.9(13) | 22.3(12) | -2.3(10) | 3.5(10) | 0.9(10) |
| O6 | 37.6(12) | 18.8(10) | 22.0(11) | 3.2(8) | 18.9(10) | 0.8(9) |
| O7 | 35.1(18) | 13.7(15) | 31.3(17) | 0 | 13.0(14) | 0 |
| N1 | 20.9(12) | 17.5(12) | 18.0(12) | 1.5(9) | 9.7(10) | -1.5(10) |
| N2 | 25.6(13) | 23.0(13) | 24.4(14) | 7.8(10) | 13.7(11) | 0.3(11) |
| N3 | 26.2(14) | 13.6(12) | 27.4(14) | -4.2(10) | 13.0(12) | -2.6(10) |
| N4 | 22.2(18) | 17.6(18) | 19.7(18) | 0 | 6.4(15) | 0 |
| C1 | 17.5(14) | 16.5(14) | 23.6(15) | 2.2(11) | 9.3(12) | 2.2(11) |
| C2 | 16.2(14) | 15.6(13) | 20.4(14) | 2.1(11) | 7.7(12) | -0.2(11) |
| C3 | 22.2(15) | 20.8(14) | 15.8(14) | 1.7(11) | 10.0(12) | 0.6(12) |
| C4 | 22.4(15) | 16.1(13) | 20.5(15) | -4.0(11) | 8.2(12) | -0.4(12) |
| C5 | 15.8(14) | 16.9(13) | 19.3(14) | 1.4(11) | 6.5(12) | -0.1(11) |
| C6 | 23.7(15) | 22.6(15) | 20.4(15) | 1.9(11) | 12.9(13) | -2.3(12) |
| C7 | 26.0(16) | 17.7(14) | 22.6(15) | -2.3(12) | 13.5(13) | -1.2(12) |
| C8 | 21.2(15) | 17.9(14) | 18.4(14) | 1.6(11) | 8.6(12) | -1.4(12) |
| C9 | 24.8(16) | 20.9(15) | 25.4(16) | 1.6(12) | 16.0(13) | -0.6(12) |
| C10 | 28.7(16) | 17.4(15) | 25.3(16) | -0.5(12) | 14.3(13) | -0.1(12) |
| C11 | 21.3(15) | 19.3(14) | 14.1(14) | 2.5(11) | 5.4(12) | -3.7(12) |
| C12 | 24.2(15) | 24.5(16) | 21.2(15) | -1.1(12) | 13.8(13) | -3.4(12) |
| C13 | 25.4(16) | 19.7(14) | 23.0(16) | -1.9(12) | 11.9(13) | 0.0(12) |
| C14 | 38.3(19) | 20.5(16) | 20.8(15) | -3.9(12) | 13.3(14) | -6.2(13) |
| C15 | 36.0(18) | 19.9(16) | 29.0(17) | -2.1(13) | 12.8(15) | -6.9(13) |
| C16 | 23.6(15) | 21.1(14) | 22.2(15) | 2.9(12) | 10.5(13) | 2.2(12) |

Table 4 Bond Lengths for MD\_4164\_BeDa.

| Atom | Atom | Length/Å |  | Atom | Atom | Length/Å |
| --- | --- | --- | --- | --- | --- | --- |
| Ce1 | O1 | 2.5836(19) |  | N1 | C14 | 1.386(3) |
| Ce1 | O11 | 2.5836(18) |  | N1 | C16 | 1.335(3) |
| Ce1 | O21 | 2.5114(18) |  | N2 | C15 | 1.364(4) |
| Ce1 | O2 | 2.5114(18) |  | N2 | C16 | 1.326(3) |
| Ce1 | O3 | 2.586(2) |  | N4 | N41 | 0.000(6) |
| Ce1 | O31 | 2.586(2) |  | C1 | C2 | 1.484(4) |
| Ce1 | O4 | 2.585(2) |  | C2 | C3 | 1.392(4) |
| Ce1 | O41 | 2.585(2) |  | C2 | C7 | 1.402(4) |
| Ce1 | O6 | 2.5948(19) |  | C3 | C4 | 1.382(4) |
| Ce1 | O61 | 2.5948(19) |  | C4 | C5 | 1.396(4) |
| O1 | C1 | 1.274(3) |  | C5 | C6 | 1.398(4) |
| O2 | C1 | 1.264(3) |  | C5 | C8 | 1.488(4) |
| O3 | N3 | 1.273(3) |  | C6 | C7 | 1.384(4) |
| O4 | N3 | 1.275(3) |  | C8 | C9 | 1.399(4) |
| O5 | N3 | 1.225(3) |  | C8 | C13 | 1.403(4) |
| O6 | N41 | 1.268(2) |  | C9 | C10 | 1.388(4) |
| O6 | N4 | 1.268(2) |  | C10 | C11 | 1.385(4) |
| O7 | N41 | 1.235(4) |  | C11 | C12 | 1.385(4) |
| O7 | N4 | 1.235(4) |  | C12 | C13 | 1.381(4) |
| N1 | C11 | 1.437(3) |  | C14 | C15 | 1.351(4) |

11/2-X,+Y,3/2-Z

Table 5 Bond Angles for MD\_4164\_BeDa.

| Atom | Atom | Atom | Angle/˚ |  | Atom | Atom | Atom | Angle/˚ |
| --- | --- | --- | --- | --- | --- | --- | --- | --- |
| O1 | Ce1 | O11 | 69.40(9) |  | C1 | O2 | Ce1 | 95.35(16) |
| O11 | Ce1 | O31 | 71.36(6) |  | N3 | O3 | Ce1 | 96.48(15) |
| O11 | Ce1 | O3 | 112.92(6) |  | N3 | O4 | Ce1 | 96.49(15) |
| O1 | Ce1 | O31 | 112.92(6) |  | N41 | O6 | Ce1 | 96.29(16) |
| O1 | Ce1 | O3 | 71.36(6) |  | N4 | O6 | Ce1 | 96.29(16) |
| O1 | Ce1 | O4 | 74.79(6) |  | N41 | O6 | N4 | 0.0(2) |
| O11 | Ce1 | O41 | 74.79(6) |  | N41 | O7 | N4 | 0.000(1) |
| O11 | Ce1 | O4 | 144.02(6) |  | C14 | N1 | C11 | 127.2(2) |
| O1 | Ce1 | O41 | 144.02(6) |  | C16 | N1 | C11 | 124.5(2) |
| O11 | Ce1 | O61 | 137.25(6) |  | C16 | N1 | C14 | 108.4(2) |
| O1 | Ce1 | O6 | 137.25(6) |  | C16 | N2 | C15 | 109.4(2) |
| O1 | Ce1 | O61 | 139.37(6) |  | O3 | N3 | O4 | 117.2(2) |
| O11 | Ce1 | O6 | 139.37(6) |  | O5 | N3 | O3 | 121.4(2) |
| O21 | Ce1 | O1 | 83.32(6) |  | O5 | N3 | O4 | 121.4(2) |
| O2 | Ce1 | O11 | 83.32(6) |  | O61 | N4 | O6 | 117.9(3) |
| O2 | Ce1 | O1 | 51.26(6) |  | O7 | N4 | O6 | 121.04(15) |
| O21 | Ce1 | O11 | 51.26(6) |  | O7 | N4 | O61 | 121.04(15) |
| O2 | Ce1 | O21 | 126.35(8) |  | N41 | N4 | O6 | 0(10) |
| O21 | Ce1 | O31 | 110.10(6) |  | N41 | N4 | O61 | 0(10) |
| O2 | Ce1 | O3 | 110.10(6) |  | N41 | N4 | O7 | 0(10) |
| O2 | Ce1 | O31 | 72.23(6) |  | O1 | C1 | C2 | 119.7(2) |
| O21 | Ce1 | O3 | 72.23(6) |  | O2 | C1 | O1 | 120.6(2) |
| O21 | Ce1 | O4 | 121.73(6) |  | O2 | C1 | C2 | 119.7(2) |
| O21 | Ce1 | O41 | 76.96(6) |  | C3 | C2 | C1 | 121.0(2) |
| O2 | Ce1 | O4 | 76.96(6) |  | C3 | C2 | C7 | 118.3(2) |
| O2 | Ce1 | O41 | 121.73(6) |  | C7 | C2 | C1 | 120.5(2) |
| O21 | Ce1 | O6 | 137.93(6) |  | C4 | C3 | C2 | 120.9(2) |
| O2 | Ce1 | O61 | 137.93(6) |  | C3 | C4 | C5 | 120.9(3) |
| O21 | Ce1 | O61 | 94.44(6) |  | C4 | C5 | C6 | 118.3(3) |
| O2 | Ce1 | O6 | 94.44(6) |  | C4 | C5 | C8 | 120.6(2) |
| O3 | Ce1 | O31 | 175.12(8) |  | C6 | C5 | C8 | 121.1(2) |
| O3 | Ce1 | O6 | 105.94(6) |  | C7 | C6 | C5 | 120.7(3) |
| O31 | Ce1 | O6 | 69.40(6) |  | C6 | C7 | C2 | 120.8(3) |
| O3 | Ce1 | O61 | 69.40(6) |  | C9 | C8 | C5 | 121.1(2) |
| O31 | Ce1 | O61 | 105.93(6) |  | C9 | C8 | C13 | 118.4(2) |
| O4 | Ce1 | O31 | 128.17(6) |  | C13 | C8 | C5 | 120.5(2) |
| O4 | Ce1 | O3 | 49.74(6) |  | C10 | C9 | C8 | 120.8(3) |
| O41 | Ce1 | O31 | 49.74(6) |  | C11 | C10 | C9 | 119.3(3) |
| O41 | Ce1 | O3 | 128.17(6) |  | C10 | C11 | N1 | 120.0(2) |
| O4 | Ce1 | O41 | 141.15(9) |  | C10 | C11 | C12 | 121.3(3) |
| O41 | Ce1 | O6 | 72.16(6) |  | C12 | C11 | N1 | 118.8(2) |
| O4 | Ce1 | O61 | 72.17(6) |  | C13 | C12 | C11 | 119.2(3) |
| O41 | Ce1 | O61 | 72.68(6) |  | C12 | C13 | C8 | 121.1(3) |
| O4 | Ce1 | O6 | 72.68(6) |  | C15 | C14 | N1 | 106.7(3) |
| O61 | Ce1 | O6 | 49.50(8) |  | C14 | C15 | N2 | 107.3(3) |
| C1 | O1 | Ce1 | 91.72(15) |  | N2 | C16 | N1 | 108.2(3) |

11/2-X,+Y,3/2-Z

Table 6 Torsion Angles for MD\_4164\_BeDa.

| A | B | C | D | Angle/˚ |  | A | B | C | D | Angle/˚ |
| --- | --- | --- | --- | --- | --- | --- | --- | --- | --- | --- |
| Ce1 | O1 | C1 | O2 | -10.5(3) |  | C4 | C5 | C6 | C7 | 2.6(4) |
| Ce1 | O1 | C1 | C2 | 166.2(2) |  | C4 | C5 | C8 | C9 | -28.9(4) |
| Ce1 | O2 | C1 | O1 | 10.9(3) |  | C4 | C5 | C8 | C13 | 151.3(3) |
| Ce1 | O2 | C1 | C2 | -165.8(2) |  | C5 | C6 | C7 | C2 | -1.5(4) |
| Ce1 | O3 | N3 | O4 | 2.6(2) |  | C5 | C8 | C9 | C10 | -179.4(3) |
| Ce1 | O3 | N3 | O5 | -176.5(2) |  | C5 | C8 | C13 | C12 | -179.5(3) |
| Ce1 | O4 | N3 | O3 | -2.6(2) |  | C6 | C5 | C8 | C9 | 150.6(3) |
| Ce1 | O4 | N3 | O5 | 176.5(2) |  | C6 | C5 | C8 | C13 | -29.2(4) |
| Ce1 | O6 | N4 | O61 | 0.002(1) |  | C7 | C2 | C3 | C4 | 1.1(4) |
| Ce1 | O6 | N4 | O7 | 180.000(1) |  | C8 | C5 | C6 | C7 | -176.9(3) |
| Ce1 | O6 | N4 | N41 | 0.0(3) |  | C8 | C9 | C10 | C11 | -0.9(4) |
| O1 | C1 | C2 | C3 | -2.7(4) |  | C9 | C8 | C13 | C12 | 0.7(4) |
| O1 | C1 | C2 | C7 | -177.4(3) |  | C9 | C10 | C11 | N1 | 178.9(3) |
| O2 | C1 | C2 | C3 | 174.0(3) |  | C9 | C10 | C11 | C12 | 0.3(4) |
| O2 | C1 | C2 | C7 | -0.6(4) |  | C10 | C11 | C12 | C13 | 0.8(4) |
| N1 | C11 | C12 | C13 | -177.9(3) |  | C11 | N1 | C14 | C15 | 179.0(3) |
| N1 | C14 | C15 | N2 | -0.4(3) |  | C11 | N1 | C16 | N2 | -178.5(2) |
| N41 | O6 | N4 | O61 | 0(100) |  | C11 | C12 | C13 | C8 | -1.3(4) |
| N41 | O6 | N4 | O7 | 0(100) |  | C13 | C8 | C9 | C10 | 0.4(4) |
| N41 | O7 | N4 | O6 | 0(100) |  | C14 | N1 | C11 | C10 | 38.0(4) |
| N41 | O7 | N4 | O61 | 0(100) |  | C14 | N1 | C11 | C12 | -143.3(3) |
| C1 | C2 | C3 | C4 | -173.6(3) |  | C14 | N1 | C16 | N2 | 0.7(3) |
| C1 | C2 | C7 | C6 | 174.4(3) |  | C15 | N2 | C16 | N1 | -0.9(3) |
| C2 | C3 | C4 | C5 | 0.1(4) |  | C16 | N1 | C11 | C10 | -142.9(3) |
| C3 | C2 | C7 | C6 | -0.4(4) |  | C16 | N1 | C11 | C12 | 35.7(4) |
| C3 | C4 | C5 | C6 | -1.9(4) |  | C16 | N1 | C14 | C15 | -0.2(3) |
| C3 | C4 | C5 | C8 | 177.6(2) |  | C16 | N2 | C15 | C14 | 0.8(3) |

11/2-X,+Y,3/2-Z

Table 7 Hydrogen Atom Coordinates (Å×104) and Isotropic Displacement Parameters (Å2×103) for MD\_4164\_BeDa.

| Atom | *x* | *y* | *z* | U(eq) |
| --- | --- | --- | --- | --- |
| H2 | 8272.52 | 14468.78 | 4055.31 | 28 |
| H3 | 4675.16 | 6817.38 | 8331.61 | 22 |
| H4 | 5442.87 | 8725.78 | 7895.72 | 23 |
| H6 | 5880.01 | 6879.4 | 5511.57 | 25 |
| H7 | 5073.77 | 4979.96 | 5928.97 | 25 |
| H9 | 5224.04 | 10539.75 | 6921.98 | 26 |
| H10 | 5912.37 | 12480.07 | 6426.57 | 27 |
| H12 | 7989.76 | 10384.73 | 5199.3 | 26 |
| H13 | 7257.37 | 8449.55 | 5646.43 | 26 |
| H14 | 7627.57 | 14175.11 | 6591.62 | 31 |
| H15 | 8323.4 | 15699.65 | 5539.87 | 33 |
| H16 | 7546.79 | 12291.41 | 4085.58 | 26 |

MD\_4164\_BeDa


# MD\_4164\_BeDa

Table 1 Crystal data and structure refinement for MD\_4164\_BeDa.

| Identification code | MD\_4164\_BeDa |
| Empirical formula | C32H24CeN7O13 |
| Formula weight | 854.70 |
| Temperature/K | 180.00(14) |
| Crystal system | monoclinic |
| Space group | P2/n |
| a/Å | 11.6491(5) |
| b/Å | 10.1346(4) |
| c/Å | 14.0436(7) |
| α/° | 90 |
| β/° | 109.849(5) |
| γ/° | 90 |
| Volume/Å3 | 1559.48(13) |
| Z | 2 |
| ρcalcg/cm3 | 1.820 |
| μ/mm‑1 | 1.544 |
| F(000) | 854.0 |
| Crystal size/mm3 | 0.2 × 0.1 × 0.05 |
| Radiation | Mo Kα (λ = 0.71073) |
| 2Θ range for data collection/° | 3.942 to 58.712 |
| Index ranges | -13 ≤ h ≤ 15, -13 ≤ k ≤ 11, -18 ≤ l ≤ 18 |
| Reflections collected | 7773 |
| Independent reflections | 3657 [Rint = 0.0356, Rsigma = 0.0525] |
| Data/restraints/parameters | 3657/0/241 |
| Goodness-of-fit on F2 | 1.050 |
| Final R indexes [I>=2σ (I)] | R1 = 0.0341, wR2 = 0.0639 |
| Final R indexes [all data] | R1 = 0.0419, wR2 = 0.0668 |
| Largest diff. peak/hole / e Å-3 | 0.63/-0.50 |
