# Supplementary material for: Novel Lanthanide (III) Complexes Derived from an Imidazole–Biphenyl–Carboxylate Ligand: Synthesis, Structure and Luminescence Properties
Source: Molecules. 2021 Nov 17;26(22):6942. doi: 10.3390/molecules26226942 (PMC8625298; doi:10.3390/molecules26226942)
Supplement: Supplementary file 1 [file molecules-26-06942-s001.zip › CRystallografic data/MD_4167_BeDa_tables.html]

MD\_4167\_BeDa


# MD\_4167\_BeDa

Table 1 Crystal data and structure refinement for MD\_4167\_BeDa.

| Identification code | MD\_4167\_BeDa |
| Empirical formula | C32H24EuN7O13 |
| Formula weight | 866.54 |
| Temperature/K | 180.00(14) |
| Crystal system | monoclinic |
| Space group | P2/n |
| a/Å | 11.6229(7) |
| b/Å | 10.0972(5) |
| c/Å | 14.0020(8) |
| α/° | 90 |
| β/° | 109.798(7) |
| γ/° | 90 |
| Volume/Å3 | 1546.14(16) |
| Z | 2 |
| ρcalcg/cm3 | 1.861 |
| μ/mm‑1 | 2.114 |
| F(000) | 864.0 |
| Crystal size/mm3 | 0.3 × 0.05 × 0.05 |
| Radiation | Mo Kα (λ = 0.71073) |
| 2Θ range for data collection/° | 3.954 to 58.262 |
| Index ranges | -15 ≤ h ≤ 15, -12 ≤ k ≤ 13, -19 ≤ l ≤ 18 |
| Reflections collected | 7681 |
| Independent reflections | 3599 [Rint = 0.0387, Rsigma = 0.0688] |
| Data/restraints/parameters | 3599/0/241 |
| Goodness-of-fit on F2 | 1.042 |
| Final R indexes [I>=2σ (I)] | R1 = 0.0419, wR2 = 0.0662 |
| Final R indexes [all data] | R1 = 0.0560, wR2 = 0.0700 |
| Largest diff. peak/hole / e Å-3 | 0.82/-0.71 |

Table 2 Fractional Atomic Coordinates (×104) and Equivalent Isotropic Displacement Parameters (Å2×103) for MD\_4167\_BeDa. Ueq is defined as 1/3 of of the trace of the orthogonalised UIJ tensor.

| Atom | *x* | *y* | *z* | U(eq) |
| --- | --- | --- | --- | --- |
| Eu1 | 7500 | 7576.3(3) | 2500 | 15.17(8) |
| O1 | 6242(2) | 5535(2) | 1853.3(19) | 20.4(6) |
| O2 | 6072(2) | 6525(2) | 3199.6(18) | 19.9(6) |
| O3 | 9545(2) | 8417(3) | 3613.9(19) | 23.1(6) |
| O4 | 8372(2) | 7672(3) | 4411.6(18) | 22.1(6) |
| O5 | 10150(2) | 8514(3) | 5271.1(19) | 31.2(7) |
| O6 | 7171(2) | 9822(3) | 3140.5(18) | 21.3(6) |
| O7 | 7500 | 11696(4) | 2500 | 26.7(9) |
| N1 | 2559(3) | -2790(3) | 4551(2) | 18.3(7) |
| N2 | 1938(3) | -4162(3) | 5454(2) | 23.0(8) |
| N3 | 9379(3) | 8207(3) | 4451(2) | 20.3(7) |
| N4 | 7500 | 10469(4) | 2500 | 19.8(10) |
| C1 | 5871(3) | 5541(4) | 2609(3) | 18.4(8) |
| C2 | 5243(3) | 4342(3) | 2832(3) | 15.0(8) |
| C3 | 5121(3) | 3206(4) | 2251(3) | 16.8(8) |
| C4 | 4662(3) | 2053(4) | 2509(3) | 16.6(8) |
| C5 | 4290(3) | 1990(4) | 3362(3) | 16.0(8) |
| C6 | 4384(3) | 3159(4) | 3925(3) | 17.8(8) |
| C7 | 4865(3) | 4308(4) | 3671(3) | 18.5(8) |
| C8 | 3837(3) | 747(4) | 3663(3) | 16.2(8) |
| C9 | 4230(3) | -480(4) | 3444(3) | 19.8(8) |
| C10 | 3819(3) | -1654(4) | 3730(3) | 21.2(9) |
| C11 | 2978(3) | -1594(4) | 4236(3) | 17.5(8) |
| C12 | 2558(3) | -390(4) | 4459(3) | 19.8(8) |
| C13 | 2996(3) | 770(4) | 4178(3) | 18.6(8) |
| C14 | 2295(3) | -3983(4) | 4028(3) | 24.6(9) |
| C15 | 1909(3) | -4824(4) | 4611(3) | 28.6(10) |
| C16 | 2340(3) | -2946(4) | 5424(3) | 20.2(8) |

Table 3 Anisotropic Displacement Parameters (Å2×103) for MD\_4167\_BeDa. The Anisotropic displacement factor exponent takes the form: -2π2[h2a\*2U11+2hka\*b\*U12+…].

| Atom | U11 | U22 | U33 | U23 | U13 | U12 |
| --- | --- | --- | --- | --- | --- | --- |
| Eu1 | 18.96(14) | 10.55(13) | 20.91(14) | 0 | 13.16(11) | 0 |
| O1 | 27.9(15) | 16.6(14) | 25.2(14) | -2.7(11) | 19.9(12) | -3.2(12) |
| O2 | 27.0(14) | 15.9(13) | 25.8(14) | -4.3(11) | 20.8(12) | -5.8(12) |
| O3 | 23.6(14) | 28.4(16) | 21.7(14) | 2.0(12) | 13.6(12) | -4.0(13) |
| O4 | 23.1(13) | 21.1(15) | 26.2(14) | 0.9(12) | 13.5(11) | -2.6(13) |
| O5 | 27.1(16) | 40.2(18) | 22.4(15) | 4.2(14) | 3.2(13) | 0.5(15) |
| O6 | 31.3(15) | 18.7(14) | 21.1(14) | 2.6(11) | 18.3(12) | 3.5(13) |
| O7 | 34(2) | 14(2) | 33(2) | 0 | 11.7(19) | 0 |
| N1 | 18.6(15) | 15.5(17) | 22.1(16) | 1.6(13) | 8.6(14) | -2.2(13) |
| N2 | 23.8(17) | 23.5(18) | 25.6(18) | 7.8(15) | 13.4(15) | -0.7(16) |
| N3 | 20.7(17) | 14.9(17) | 27.2(18) | 2.4(14) | 10.7(16) | 4.7(15) |
| N4 | 20(2) | 17(2) | 21(2) | 0 | 6(2) | 0 |
| C1 | 19.1(19) | 16.1(19) | 22(2) | 1.3(16) | 9.1(17) | 0.8(17) |
| C2 | 15.0(18) | 13.4(18) | 17.5(18) | 1.1(15) | 6.4(16) | 0.5(16) |
| C3 | 16.9(18) | 18.9(19) | 19.0(19) | 1.8(16) | 11.9(16) | -2.4(17) |
| C4 | 15.3(18) | 12.9(18) | 23(2) | -2.9(15) | 7.7(16) | 0.8(15) |
| C5 | 13.9(18) | 18.3(18) | 17.3(18) | 1.8(15) | 7.1(16) | -1.1(16) |
| C6 | 20.8(19) | 19.0(19) | 19.1(19) | 0.0(16) | 13.7(17) | -0.8(17) |
| C7 | 19.4(19) | 17.1(19) | 22(2) | -3.2(16) | 10.6(17) | -1.1(17) |
| C8 | 12.4(18) | 17.2(19) | 19.8(19) | 0.0(16) | 6.5(16) | -0.1(16) |
| C9 | 21(2) | 20(2) | 25(2) | 3.1(16) | 17.1(17) | -1.1(17) |
| C10 | 26(2) | 17(2) | 26(2) | -0.6(17) | 15.5(18) | 0.2(18) |
| C11 | 13.9(19) | 21(2) | 15.9(18) | 6.7(16) | 3.3(16) | -6.4(17) |
| C12 | 20(2) | 21(2) | 23(2) | 0.6(16) | 12.4(17) | -2.6(17) |
| C13 | 19.3(19) | 16.9(19) | 22(2) | -2.8(16) | 9.9(17) | 2.6(17) |
| C14 | 32(2) | 18(2) | 25(2) | -4.5(17) | 10.7(19) | -7.4(19) |
| C15 | 33(2) | 22(2) | 32(2) | -0.5(19) | 12(2) | -6(2) |
| C16 | 20.3(19) | 20(2) | 23(2) | 3.0(16) | 9.8(17) | 3.7(17) |

Table 4 Bond Lengths for MD\_4167\_BeDa.

| Atom | Atom | Length/Å |  | Atom | Atom | Length/Å |
| --- | --- | --- | --- | --- | --- | --- |
| Eu1 | O1 | 2.512(2) |  | N1 | C14 | 1.389(5) |
| Eu1 | O11 | 2.512(2) |  | N1 | C16 | 1.339(5) |
| Eu1 | O2 | 2.437(2) |  | N2 | C15 | 1.347(5) |
| Eu1 | O21 | 2.437(2) |  | N2 | C16 | 1.320(5) |
| Eu1 | O3 | 2.508(2) |  | N4 | N41 | 0.000(9) |
| Eu1 | O31 | 2.508(2) |  | C1 | C2 | 1.499(5) |
| Eu1 | O4 | 2.523(2) |  | C2 | C3 | 1.386(5) |
| Eu1 | O41 | 2.523(2) |  | C2 | C7 | 1.387(5) |
| Eu1 | O61 | 2.515(3) |  | C3 | C4 | 1.379(5) |
| Eu1 | O6 | 2.515(3) |  | C4 | C5 | 1.403(5) |
| O1 | C1 | 1.271(4) |  | C5 | C6 | 1.402(5) |
| O2 | C1 | 1.263(4) |  | C5 | C8 | 1.476(5) |
| O3 | N3 | 1.268(4) |  | C6 | C7 | 1.386(5) |
| O4 | N3 | 1.273(4) |  | C8 | C9 | 1.390(5) |
| O5 | N3 | 1.234(4) |  | C8 | C13 | 1.398(5) |
| O6 | N41 | 1.269(3) |  | C9 | C10 | 1.387(5) |
| O6 | N4 | 1.269(3) |  | C10 | C11 | 1.390(5) |
| O7 | N41 | 1.239(5) |  | C11 | C12 | 1.385(5) |
| O7 | N4 | 1.239(5) |  | C12 | C13 | 1.385(5) |
| N1 | C11 | 1.428(4) |  | C14 | C15 | 1.356(5) |

13/2-X,+Y,1/2-Z

Table 5 Bond Angles for MD\_4167\_BeDa.

| Atom | Atom | Atom | Angle/˚ |  | Atom | Atom | Atom | Angle/˚ |
| --- | --- | --- | --- | --- | --- | --- | --- | --- |
| O1 | Eu1 | O11 | 69.71(11) |  | C1 | O2 | Eu1 | 94.6(2) |
| O1 | Eu1 | O41 | 70.92(8) |  | N3 | O3 | Eu1 | 96.3(2) |
| O11 | Eu1 | O4 | 70.92(8) |  | N3 | O4 | Eu1 | 95.4(2) |
| O1 | Eu1 | O4 | 112.92(8) |  | N41 | O6 | Eu1 | 95.4(2) |
| O11 | Eu1 | O41 | 112.92(8) |  | N4 | O6 | Eu1 | 95.4(2) |
| O11 | Eu1 | O6 | 138.73(8) |  | N41 | O6 | N4 | 0.0(3) |
| O1 | Eu1 | O6 | 136.73(8) |  | N41 | O7 | N4 | 0.000(1) |
| O11 | Eu1 | O61 | 136.73(8) |  | C14 | N1 | C11 | 127.4(3) |
| O1 | Eu1 | O61 | 138.73(8) |  | C16 | N1 | C11 | 124.9(3) |
| O21 | Eu1 | O11 | 52.79(8) |  | C16 | N1 | C14 | 107.7(3) |
| O21 | Eu1 | O1 | 83.70(8) |  | C16 | N2 | C15 | 109.8(3) |
| O2 | Eu1 | O1 | 52.79(8) |  | O3 | N3 | O4 | 117.2(3) |
| O2 | Eu1 | O11 | 83.70(8) |  | O5 | N3 | O3 | 121.9(3) |
| O21 | Eu1 | O2 | 128.38(12) |  | O5 | N3 | O4 | 120.9(3) |
| O21 | Eu1 | O3 | 76.50(9) |  | O61 | N4 | O6 | 118.0(4) |
| O2 | Eu1 | O3 | 121.88(8) |  | O7 | N4 | O6 | 121.0(2) |
| O2 | Eu1 | O31 | 76.50(9) |  | O7 | N4 | O61 | 121.0(2) |
| O21 | Eu1 | O31 | 121.88(8) |  | N41 | N4 | O6 | 0(10) |
| O2 | Eu1 | O41 | 111.07(8) |  | N41 | N4 | O61 | 0(10) |
| O2 | Eu1 | O4 | 70.95(8) |  | N41 | N4 | O7 | 0(10) |
| O21 | Eu1 | O41 | 70.95(8) |  | O1 | C1 | C2 | 119.7(3) |
| O21 | Eu1 | O4 | 111.07(8) |  | O2 | C1 | O1 | 120.5(3) |
| O2 | Eu1 | O6 | 92.45(8) |  | O2 | C1 | C2 | 119.6(3) |
| O2 | Eu1 | O61 | 137.94(8) |  | C3 | C2 | C1 | 120.4(3) |
| O21 | Eu1 | O61 | 92.45(8) |  | C3 | C2 | C7 | 118.6(3) |
| O21 | Eu1 | O6 | 137.94(8) |  | C7 | C2 | C1 | 120.8(3) |
| O3 | Eu1 | O11 | 74.95(8) |  | C4 | C3 | C2 | 121.1(3) |
| O3 | Eu1 | O1 | 144.60(9) |  | C3 | C4 | C5 | 121.3(3) |
| O31 | Eu1 | O11 | 144.61(9) |  | C4 | C5 | C8 | 121.6(3) |
| O31 | Eu1 | O1 | 74.95(8) |  | C6 | C5 | C4 | 116.9(3) |
| O3 | Eu1 | O31 | 140.43(13) |  | C6 | C5 | C8 | 121.5(3) |
| O3 | Eu1 | O4 | 51.07(8) |  | C7 | C6 | C5 | 121.5(3) |
| O31 | Eu1 | O41 | 51.07(8) |  | C6 | C7 | C2 | 120.6(4) |
| O3 | Eu1 | O41 | 127.05(8) |  | C9 | C8 | C5 | 121.3(3) |
| O31 | Eu1 | O4 | 127.05(8) |  | C9 | C8 | C13 | 117.9(3) |
| O3 | Eu1 | O61 | 71.94(8) |  | C13 | C8 | C5 | 120.8(3) |
| O31 | Eu1 | O6 | 71.94(8) |  | C10 | C9 | C8 | 121.8(3) |
| O31 | Eu1 | O61 | 72.52(8) |  | C9 | C10 | C11 | 118.8(4) |
| O3 | Eu1 | O6 | 72.52(8) |  | C10 | C11 | N1 | 119.6(4) |
| O4 | Eu1 | O41 | 175.63(12) |  | C12 | C11 | N1 | 119.4(3) |
| O6 | Eu1 | O41 | 106.80(8) |  | C12 | C11 | C10 | 121.0(4) |
| O6 | Eu1 | O4 | 69.04(8) |  | C11 | C12 | C13 | 119.1(3) |
| O61 | Eu1 | O41 | 69.04(8) |  | C12 | C13 | C8 | 121.4(4) |
| O61 | Eu1 | O4 | 106.80(8) |  | C15 | C14 | N1 | 106.5(4) |
| O61 | Eu1 | O6 | 51.25(11) |  | N2 | C15 | C14 | 107.5(4) |
| C1 | O1 | Eu1 | 91.0(2) |  | N2 | C16 | N1 | 108.4(3) |

13/2-X,+Y,1/2-Z

Table 6 Hydrogen Bonds for MD\_4167\_BeDa.

| D | H | A | d(D-H)/Å | d(H-A)/Å | d(D-A)/Å | D-H-A/° |
| --- | --- | --- | --- | --- | --- | --- |
| N2 | H2 | O11 | 0.86 | 1.89 | 2.737(4) | 167.8 |
| C15 | H15 | O42 | 0.93 | 2.56 | 3.248(5) | 131.6 |
| C16 | H16 | O73 | 0.93 | 2.27 | 3.116(4) | 150.5 |

1-1/2+X,-Y,1/2+Z; 21-X,-Y,1-Z; 31-X,1-Y,1-Z

Table 7 Torsion Angles for MD\_4167\_BeDa.

| A | B | C | D | Angle/˚ |  | A | B | C | D | Angle/˚ |
| --- | --- | --- | --- | --- | --- | --- | --- | --- | --- | --- |
| Eu1 | O1 | C1 | O2 | 10.5(3) |  | C4 | C5 | C6 | C7 | -2.3(5) |
| Eu1 | O1 | C1 | C2 | -166.4(3) |  | C4 | C5 | C8 | C9 | 28.6(5) |
| Eu1 | O2 | C1 | O1 | -10.9(4) |  | C4 | C5 | C8 | C13 | -151.3(3) |
| Eu1 | O2 | C1 | C2 | 166.0(3) |  | C5 | C6 | C7 | C2 | 1.5(5) |
| Eu1 | O3 | N3 | O4 | 2.0(3) |  | C5 | C8 | C9 | C10 | 179.5(3) |
| Eu1 | O3 | N3 | O5 | -177.4(3) |  | C5 | C8 | C13 | C12 | 179.6(3) |
| Eu1 | O4 | N3 | O3 | -2.0(3) |  | C6 | C5 | C8 | C9 | -150.4(4) |
| Eu1 | O4 | N3 | O5 | 177.4(3) |  | C6 | C5 | C8 | C13 | 29.7(5) |
| Eu1 | O6 | N4 | O61 | -0.003(2) |  | C7 | C2 | C3 | C4 | -1.6(5) |
| Eu1 | O6 | N4 | O7 | 180.000(1) |  | C8 | C5 | C6 | C7 | 176.8(3) |
| Eu1 | O6 | N4 | N41 | 0.0(4) |  | C8 | C9 | C10 | C11 | 0.9(6) |
| O1 | C1 | C2 | C3 | 3.3(5) |  | C9 | C8 | C13 | C12 | -0.4(5) |
| O1 | C1 | C2 | C7 | 177.9(3) |  | C9 | C10 | C11 | N1 | -179.0(3) |
| O2 | C1 | C2 | C3 | -173.7(3) |  | C9 | C10 | C11 | C12 | -0.3(5) |
| O2 | C1 | C2 | C7 | 1.0(5) |  | C10 | C11 | C12 | C13 | -0.6(5) |
| N1 | C11 | C12 | C13 | 178.1(3) |  | C11 | N1 | C14 | C15 | -178.9(3) |
| N1 | C14 | C15 | N2 | 0.0(4) |  | C11 | N1 | C16 | N2 | 178.5(3) |
| N41 | O6 | N4 | O61 | 0(100) |  | C11 | C12 | C13 | C8 | 0.9(5) |
| N41 | O6 | N4 | O7 | 0(100) |  | C13 | C8 | C9 | C10 | -0.6(5) |
| N41 | O7 | N4 | O6 | 0(81) |  | C14 | N1 | C11 | C10 | -38.6(5) |
| N41 | O7 | N4 | O61 | 0(100) |  | C14 | N1 | C11 | C12 | 142.7(4) |
| C1 | C2 | C3 | C4 | 173.2(3) |  | C14 | N1 | C16 | N2 | -1.0(4) |
| C1 | C2 | C7 | C6 | -174.3(3) |  | C15 | N2 | C16 | N1 | 1.0(4) |
| C2 | C3 | C4 | C5 | 0.7(5) |  | C16 | N1 | C11 | C10 | 142.0(4) |
| C3 | C2 | C7 | C6 | 0.5(5) |  | C16 | N1 | C11 | C12 | -36.7(5) |
| C3 | C4 | C5 | C6 | 1.2(5) |  | C16 | N1 | C14 | C15 | 0.6(4) |
| C3 | C4 | C5 | C8 | -177.9(3) |  | C16 | N2 | C15 | C14 | -0.6(4) |

13/2-X,+Y,1/2-Z

Table 8 Hydrogen Atom Coordinates (Å×104) and Isotropic Displacement Parameters (Å2×103) for MD\_4167\_BeDa.

| Atom | *x* | *y* | *z* | U(eq) |
| --- | --- | --- | --- | --- |
| H2 | 1727.58 | -4484.23 | 5939.01 | 28 |
| H3 | 5352.6 | 3221.09 | 1676.67 | 20 |
| H4 | 4598.47 | 1301.51 | 2110.22 | 20 |
| H6 | 4117.45 | 3162.65 | 4480.69 | 21 |
| H7 | 4934.03 | 5063.9 | 4066.05 | 22 |
| H9 | 4783.9 | -514.45 | 3095.84 | 24 |
| H10 | 4100.15 | -2465.56 | 3585.16 | 25 |
| H12 | 1989.27 | -359.06 | 4792.15 | 24 |
| H13 | 2724.57 | 1580.03 | 4335.2 | 22 |
| H14 | 2369.66 | -4167.72 | 3400.66 | 30 |
| H15 | 1666.68 | -5699.08 | 4456.61 | 34 |
| H16 | 2451.49 | -2304.67 | 5924.35 | 24 |

MD\_4167\_BeDa


# MD\_4167\_BeDa

Table 1 Crystal data and structure refinement for MD\_4167\_BeDa.

| Identification code | MD\_4167\_BeDa |
| Empirical formula | C32H24EuN7O13 |
| Formula weight | 866.54 |
| Temperature/K | 180.00(14) |
| Crystal system | monoclinic |
| Space group | P2/n |
| a/Å | 11.6229(7) |
| b/Å | 10.0972(5) |
| c/Å | 14.0020(8) |
| α/° | 90 |
| β/° | 109.798(7) |
| γ/° | 90 |
| Volume/Å3 | 1546.14(16) |
| Z | 2 |
| ρcalcg/cm3 | 1.861 |
| μ/mm‑1 | 2.114 |
| F(000) | 864.0 |
| Crystal size/mm3 | 0.3 × 0.05 × 0.05 |
| Radiation | Mo Kα (λ = 0.71073) |
| 2Θ range for data collection/° | 3.954 to 58.262 |
| Index ranges | -15 ≤ h ≤ 15, -12 ≤ k ≤ 13, -19 ≤ l ≤ 18 |
| Reflections collected | 7681 |
| Independent reflections | 3599 [Rint = 0.0387, Rsigma = 0.0688] |
| Data/restraints/parameters | 3599/0/241 |
| Goodness-of-fit on F2 | 1.042 |
| Final R indexes [I>=2σ (I)] | R1 = 0.0419, wR2 = 0.0662 |
| Final R indexes [all data] | R1 = 0.0560, wR2 = 0.0700 |
| Largest diff. peak/hole / e Å-3 | 0.82/-0.71 |
