# Supplementary material for: Novel Lanthanide (III) Complexes Derived from an Imidazole–Biphenyl–Carboxylate Ligand: Synthesis, Structure and Luminescence Properties
Source: Molecules. 2021 Nov 17;26(22):6942. doi: 10.3390/molecules26226942 (PMC8625298; doi:10.3390/molecules26226942)
Supplement: Supplementary file 1 [file molecules-26-06942-s001.zip › CRystallografic data/MD_4168_BeDa_tables.html]

MD\_4168\_BeDa


# MD\_4168\_BeDa

Table 1 Crystal data and structure refinement for MD\_4168\_BeDa.

| Identification code | MD\_4168\_BeDa |
| Empirical formula | C32H24GdN7O13 |
| Formula weight | 871.83 |
| Temperature/K | 180.05(10) |
| Crystal system | monoclinic |
| Space group | P2/n |
| a/Å | 11.6154(7) |
| b/Å | 10.0797(5) |
| c/Å | 13.9905(7) |
| α/° | 90 |
| β/° | 109.817(7) |
| γ/° | 90 |
| Volume/Å3 | 1541.00(16) |
| Z | 2 |
| ρcalcg/cm3 | 1.879 |
| μ/mm‑1 | 2.238 |
| F(000) | 866.0 |
| Crystal size/mm3 | 0.3 × 0.05 × 0.05 |
| Radiation | Mo Kα (λ = 0.71073) |
| 2Θ range for data collection/° | 3.956 to 50.048 |
| Index ranges | -13 ≤ h ≤ 13, -11 ≤ k ≤ 11, -16 ≤ l ≤ 16 |
| Reflections collected | 5840 |
| Independent reflections | 2712 [Rint = 0.0267, Rsigma = 0.0498] |
| Data/restraints/parameters | 2712/0/241 |
| Goodness-of-fit on F2 | 1.057 |
| Final R indexes [I>=2σ (I)] | R1 = 0.0300, wR2 = 0.0453 |
| Final R indexes [all data] | R1 = 0.0386, wR2 = 0.0476 |
| Largest diff. peak/hole / e Å-3 | 0.49/-0.42 |

Table 2 Fractional Atomic Coordinates (×104) and Equivalent Isotropic Displacement Parameters (Å2×103) for MD\_4168\_BeDa. Ueq is defined as 1/3 of of the trace of the orthogonalised UIJ tensor.

| Atom | *x* | *y* | *z* | U(eq) |
| --- | --- | --- | --- | --- |
| Gd1 | 7500 | 7575.4(3) | 2500 | 15.98(8) |
| O1 | 6252(2) | 5540(2) | 1856.1(15) | 21.5(6) |
| O2 | 6084.0(19) | 6529(2) | 3205.0(15) | 20.1(5) |
| O3 | 9536(2) | 8415(2) | 3604.0(15) | 23.9(6) |
| O4 | 8368.6(19) | 7672(2) | 4406.5(15) | 22.5(5) |
| O5 | 10149(2) | 8506(3) | 5262.5(16) | 31.3(7) |
| O6 | 7170(2) | 9812(2) | 3140.1(14) | 22.2(6) |
| O7 | 7500 | 11683(3) | 2500 | 26.7(8) |
| N1 | 2554(2) | -2792(3) | 4549.8(18) | 19.6(7) |
| N2 | 1938(2) | -4158(3) | 5452(2) | 23.5(7) |
| N3 | 9373(3) | 8207(3) | 4446(2) | 22.6(7) |
| N4 | 7500 | 10466(4) | 2500 | 21.1(10) |
| C1 | 5869(3) | 5547(3) | 2614(2) | 19.3(8) |
| C2 | 5251(3) | 4352(3) | 2830(2) | 16.7(8) |
| C3 | 5126(3) | 3206(3) | 2247(2) | 19.7(8) |
| C4 | 4663(3) | 2050(3) | 2508(2) | 18.8(8) |
| C5 | 4292(3) | 2000(3) | 3363(2) | 17.6(8) |
| C6 | 4388(3) | 3166(3) | 3928(2) | 21.4(8) |
| C7 | 4862(3) | 4307(3) | 3669(2) | 19.9(8) |
| C8 | 3841(3) | 748(3) | 3660(2) | 18.1(8) |
| C9 | 4231(3) | -486(3) | 3441(2) | 21.5(8) |
| C10 | 3818(3) | -1645(3) | 3725(2) | 21.7(8) |
| C11 | 2980(3) | -1590(3) | 4231(2) | 16.9(8) |
| C12 | 2569(3) | -386(3) | 4460(2) | 20.1(8) |
| C13 | 3002(3) | 773(3) | 4180(2) | 19.7(8) |
| C14 | 2289(3) | -3977(4) | 4025(2) | 27.1(9) |
| C15 | 1906(3) | -4830(4) | 4603(2) | 28.5(9) |
| C16 | 2342(3) | -2940(3) | 5424(2) | 20.1(8) |

Table 3 Anisotropic Displacement Parameters (Å2×103) for MD\_4168\_BeDa. The Anisotropic displacement factor exponent takes the form: -2π2[h2a\*2U11+2hka\*b\*U12+…].

| Atom | U11 | U22 | U33 | U23 | U13 | U12 |
| --- | --- | --- | --- | --- | --- | --- |
| Gd1 | 20.25(13) | 11.14(13) | 21.23(12) | 0 | 13.15(9) | 0 |
| O1 | 30.6(14) | 17.4(14) | 24.8(11) | -2.7(10) | 20.0(11) | -4.5(12) |
| O2 | 27.5(13) | 13.4(13) | 26.0(12) | -4.3(11) | 18.0(10) | -5.6(12) |
| O3 | 27.4(13) | 26.7(15) | 23.3(12) | 0.3(12) | 16.0(11) | -2.7(13) |
| O4 | 21.9(12) | 19.5(14) | 29.7(12) | 0.9(12) | 13.5(10) | -1.7(13) |
| O5 | 28.4(15) | 36.0(17) | 24.3(12) | 4.1(13) | 2.3(11) | -0.8(14) |
| O6 | 32.1(14) | 18.6(14) | 22.3(12) | 3.1(11) | 17.6(11) | 0.5(12) |
| O7 | 36(2) | 15(2) | 31.1(18) | 0 | 13.2(16) | 0 |
| N1 | 21.7(15) | 18.6(17) | 21.3(14) | 2.1(13) | 11.1(12) | -1.8(14) |
| N2 | 25.4(17) | 21.6(18) | 28.9(15) | 9.2(14) | 16.2(13) | -0.5(15) |
| N3 | 22.7(17) | 17.5(17) | 29.0(16) | 2.6(14) | 10.7(14) | 8.8(15) |
| N4 | 24(2) | 16(2) | 22(2) | 0 | 5.7(19) | 0 |
| C1 | 16.2(18) | 16(2) | 26.4(18) | 2.5(17) | 8.4(15) | 3.0(17) |
| C2 | 14.2(18) | 15.6(19) | 19.4(16) | 2.8(15) | 4.7(14) | -0.6(16) |
| C3 | 20.3(19) | 21(2) | 19.7(17) | -0.2(16) | 9.8(15) | -3.7(17) |
| C4 | 20.5(18) | 15.0(19) | 21.0(16) | -3.1(15) | 7.2(14) | -2.8(16) |
| C5 | 12.9(17) | 18(2) | 20.6(16) | 1.5(15) | 4.7(14) | -1.3(16) |
| C6 | 23(2) | 23(2) | 22.8(17) | -1.2(17) | 12.9(16) | -1.3(18) |
| C7 | 23(2) | 16(2) | 22.6(17) | -2.8(16) | 10.4(15) | 2.0(17) |
| C8 | 17.6(19) | 19(2) | 18.0(16) | 0.1(16) | 6.7(14) | -3.3(17) |
| C9 | 23.1(19) | 23(2) | 24.8(18) | 0.4(17) | 16.4(15) | -2.8(18) |
| C10 | 26(2) | 16(2) | 28.5(18) | 1.4(17) | 15.6(16) | 3.0(18) |
| C11 | 18.8(19) | 16.8(19) | 14.5(16) | 3.4(15) | 4.8(14) | -2.7(17) |
| C12 | 21.3(19) | 22(2) | 21.0(17) | 1.6(16) | 12.5(15) | -1.9(18) |
| C13 | 23.5(19) | 14.6(19) | 22.3(17) | -3.9(16) | 9.6(15) | 1.3(18) |
| C14 | 36(2) | 21(2) | 25.8(18) | -6.6(18) | 12.8(17) | -8(2) |
| C15 | 39(2) | 20(2) | 27.3(19) | -1.5(18) | 11.9(17) | -6(2) |
| C16 | 22.3(18) | 20(2) | 20.4(16) | 1.5(16) | 10.5(15) | 2.6(17) |

Table 4 Bond Lengths for MD\_4168\_BeDa.

| Atom | Atom | Length/Å |  | Atom | Atom | Length/Å |
| --- | --- | --- | --- | --- | --- | --- |
| Gd1 | O1 | 2.497(2) |  | N1 | C14 | 1.381(4) |
| Gd1 | O11 | 2.497(2) |  | N1 | C16 | 1.335(4) |
| Gd1 | O2 | 2.427(2) |  | N2 | C15 | 1.357(4) |
| Gd1 | O21 | 2.427(2) |  | N2 | C16 | 1.320(4) |
| Gd1 | O31 | 2.494(2) |  | C1 | C2 | 1.485(4) |
| Gd1 | O3 | 2.494(2) |  | C2 | C3 | 1.392(4) |
| Gd1 | O4 | 2.5135(19) |  | C2 | C7 | 1.394(4) |
| Gd1 | O41 | 2.5135(19) |  | C3 | C4 | 1.384(4) |
| Gd1 | O6 | 2.503(2) |  | C4 | C5 | 1.402(4) |
| Gd1 | O61 | 2.503(2) |  | C5 | C6 | 1.400(4) |
| Gd1 | C11 | 2.828(3) |  | C5 | C8 | 1.480(4) |
| Gd1 | C1 | 2.828(3) |  | C6 | C7 | 1.375(4) |
| O1 | C1 | 1.282(4) |  | C8 | C9 | 1.393(4) |
| O2 | C1 | 1.259(4) |  | C8 | C13 | 1.399(4) |
| O3 | N3 | 1.272(3) |  | C9 | C10 | 1.372(4) |
| O4 | N3 | 1.269(3) |  | C10 | C11 | 1.385(4) |
| O5 | N3 | 1.229(3) |  | C11 | C12 | 1.381(4) |
| O6 | N4 | 1.271(3) |  | C12 | C13 | 1.380(4) |
| O7 | N4 | 1.227(5) |  | C14 | C15 | 1.356(5) |
| N1 | C11 | 1.436(4) |  |  |  |  |

13/2-X,+Y,1/2-Z

Table 5 Bond Angles for MD\_4168\_BeDa.

| Atom | Atom | Atom | Angle/˚ |  | Atom | Atom | Atom | Angle/˚ |
| --- | --- | --- | --- | --- | --- | --- | --- | --- |
| O1 | Gd1 | O11 | 69.52(10) |  | O6 | Gd1 | O41 | 106.83(7) |
| O1 | Gd1 | O4 | 112.95(7) |  | O61 | Gd1 | O4 | 106.84(7) |
| O11 | Gd1 | O4 | 70.97(7) |  | O61 | Gd1 | O6 | 51.47(10) |
| O11 | Gd1 | O41 | 112.95(7) |  | O6 | Gd1 | C11 | 149.10(8) |
| O1 | Gd1 | O41 | 70.98(7) |  | O61 | Gd1 | C11 | 116.38(9) |
| O11 | Gd1 | O6 | 138.72(7) |  | O61 | Gd1 | C1 | 149.10(8) |
| O1 | Gd1 | O61 | 138.72(7) |  | O6 | Gd1 | C1 | 116.38(9) |
| O11 | Gd1 | O61 | 136.77(7) |  | C11 | Gd1 | C1 | 87.41(14) |
| O1 | Gd1 | O6 | 136.77(7) |  | C1 | O1 | Gd1 | 91.06(19) |
| O1 | Gd1 | C1 | 26.95(8) |  | C1 | O2 | Gd1 | 94.9(2) |
| O11 | Gd1 | C11 | 26.95(8) |  | N3 | O3 | Gd1 | 96.31(17) |
| O1 | Gd1 | C11 | 72.76(8) |  | N3 | O4 | Gd1 | 95.43(16) |
| O11 | Gd1 | C1 | 72.76(8) |  | N4 | O6 | Gd1 | 95.5(2) |
| O2 | Gd1 | O1 | 53.07(7) |  | C14 | N1 | C11 | 127.1(3) |
| O21 | Gd1 | O1 | 83.49(7) |  | C16 | N1 | C11 | 124.7(3) |
| O2 | Gd1 | O11 | 83.49(7) |  | C16 | N1 | C14 | 108.3(3) |
| O21 | Gd1 | O11 | 53.07(7) |  | C16 | N2 | C15 | 110.0(3) |
| O2 | Gd1 | O21 | 128.47(10) |  | O3 | N3 | Gd1 | 58.05(14) |
| O21 | Gd1 | O31 | 121.79(7) |  | O4 | N3 | Gd1 | 58.94(14) |
| O2 | Gd1 | O31 | 76.59(7) |  | O4 | N3 | O3 | 117.0(2) |
| O2 | Gd1 | O3 | 121.79(7) |  | O5 | N3 | Gd1 | 178.4(2) |
| O21 | Gd1 | O3 | 76.59(7) |  | O5 | N3 | O3 | 121.9(3) |
| O2 | Gd1 | O4 | 70.68(7) |  | O5 | N3 | O4 | 121.2(3) |
| O2 | Gd1 | O41 | 111.39(7) |  | O61 | N4 | Gd1 | 58.77(19) |
| O21 | Gd1 | O41 | 70.68(7) |  | O6 | N4 | Gd1 | 58.77(19) |
| O21 | Gd1 | O4 | 111.39(7) |  | O6 | N4 | O61 | 117.5(4) |
| O21 | Gd1 | O61 | 92.24(7) |  | O7 | N4 | Gd1 | 180.0 |
| O2 | Gd1 | O6 | 92.25(7) |  | O7 | N4 | O6 | 121.23(19) |
| O21 | Gd1 | O6 | 138.07(7) |  | O7 | N4 | O61 | 121.23(19) |
| O2 | Gd1 | O61 | 138.07(7) |  | O1 | C1 | Gd1 | 61.99(17) |
| O2 | Gd1 | C11 | 105.54(9) |  | O1 | C1 | C2 | 119.4(3) |
| O21 | Gd1 | C1 | 105.54(9) |  | O2 | C1 | Gd1 | 58.78(17) |
| O2 | Gd1 | C1 | 26.32(8) |  | O2 | C1 | O1 | 120.1(3) |
| O21 | Gd1 | C11 | 26.32(8) |  | O2 | C1 | C2 | 120.4(3) |
| O31 | Gd1 | O1 | 75.09(7) |  | C2 | C1 | Gd1 | 167.8(2) |
| O3 | Gd1 | O11 | 75.09(7) |  | C3 | C2 | C1 | 121.0(3) |
| O3 | Gd1 | O1 | 144.56(8) |  | C3 | C2 | C7 | 117.8(3) |
| O31 | Gd1 | O11 | 144.56(8) |  | C7 | C2 | C1 | 120.9(3) |
| O31 | Gd1 | O3 | 140.34(11) |  | C4 | C3 | C2 | 121.1(3) |
| O31 | Gd1 | O4 | 126.82(7) |  | C3 | C4 | C5 | 121.0(3) |
| O3 | Gd1 | O41 | 126.82(7) |  | C4 | C5 | C8 | 120.7(3) |
| O31 | Gd1 | O41 | 51.27(7) |  | C6 | C5 | C4 | 117.5(3) |
| O3 | Gd1 | O4 | 51.27(7) |  | C6 | C5 | C8 | 121.7(3) |
| O3 | Gd1 | O6 | 72.59(7) |  | C7 | C6 | C5 | 121.0(3) |
| O31 | Gd1 | O61 | 72.59(7) |  | C6 | C7 | C2 | 121.5(3) |
| O31 | Gd1 | O6 | 71.82(7) |  | C9 | C8 | C5 | 121.8(3) |
| O3 | Gd1 | O61 | 71.82(7) |  | C9 | C8 | C13 | 117.8(3) |
| O3 | Gd1 | C1 | 136.30(8) |  | C13 | C8 | C5 | 120.4(3) |
| O31 | Gd1 | C11 | 136.30(8) |  | C10 | C9 | C8 | 121.7(3) |
| O31 | Gd1 | C1 | 76.55(8) |  | C9 | C10 | C11 | 119.2(3) |
| O3 | Gd1 | C11 | 76.56(8) |  | C10 | C11 | N1 | 120.0(3) |
| O4 | Gd1 | O41 | 175.54(11) |  | C12 | C11 | N1 | 119.2(3) |
| O4 | Gd1 | C11 | 93.01(8) |  | C12 | C11 | C10 | 120.8(3) |
| O4 | Gd1 | C1 | 90.21(8) |  | C13 | C12 | C11 | 119.4(3) |
| O41 | Gd1 | C11 | 90.21(8) |  | C12 | C13 | C8 | 121.1(3) |
| O41 | Gd1 | C1 | 93.01(8) |  | C15 | C14 | N1 | 106.8(3) |
| O6 | Gd1 | O4 | 68.92(7) |  | C14 | C15 | N2 | 106.8(3) |
| O61 | Gd1 | O41 | 68.92(7) |  | N2 | C16 | N1 | 108.0(3) |

13/2-X,+Y,1/2-Z

Table 6 Hydrogen Bonds for MD\_4168\_BeDa.

| D | H | A | d(D-H)/Å | d(H-A)/Å | d(D-A)/Å | D-H-A/° |
| --- | --- | --- | --- | --- | --- | --- |
| N2 | H2 | O11 | 0.86 | 1.89 | 2.737(3) | 167.4 |
| C15 | H15 | O42 | 0.93 | 2.56 | 3.244(4) | 131.1 |
| C16 | H16 | O73 | 0.93 | 2.27 | 3.117(3) | 150.5 |

1-1/2+X,-Y,1/2+Z; 21-X,-Y,1-Z; 31-X,1-Y,1-Z

Table 7 Torsion Angles for MD\_4168\_BeDa.

| A | B | C | D | Angle/˚ |  | A | B | C | D | Angle/˚ |
| --- | --- | --- | --- | --- | --- | --- | --- | --- | --- | --- |
| Gd1 | O1 | C1 | O2 | 9.5(3) |  | C4 | C5 | C8 | C13 | -151.7(3) |
| Gd1 | O1 | C1 | C2 | -166.1(3) |  | C5 | C6 | C7 | C2 | 0.8(5) |
| Gd1 | O2 | C1 | O1 | -9.8(3) |  | C5 | C8 | C9 | C10 | 179.5(3) |
| Gd1 | O2 | C1 | C2 | 165.8(3) |  | C5 | C8 | C13 | C12 | 179.8(3) |
| Gd1 | O3 | N3 | O4 | 2.1(3) |  | C6 | C5 | C8 | C9 | -150.2(3) |
| Gd1 | O3 | N3 | O5 | -178.1(3) |  | C6 | C5 | C8 | C13 | 29.5(5) |
| Gd1 | O4 | N3 | O3 | -2.1(3) |  | C7 | C2 | C3 | C4 | -1.7(5) |
| Gd1 | O4 | N3 | O5 | 178.1(3) |  | C8 | C5 | C6 | C7 | 177.0(3) |
| Gd1 | O6 | N4 | O61 | 0.002(1) |  | C8 | C9 | C10 | C11 | 0.8(5) |
| Gd1 | O6 | N4 | O7 | 180.000(1) |  | C9 | C8 | C13 | C12 | -0.5(5) |
| Gd1 | C1 | C2 | C3 | -90.4(11) |  | C9 | C10 | C11 | N1 | -178.9(3) |
| Gd1 | C1 | C2 | C7 | 84.6(12) |  | C9 | C10 | C11 | C12 | -0.7(5) |
| O1 | C1 | C2 | C3 | 2.7(5) |  | C10 | C11 | C12 | C13 | -0.1(5) |
| O1 | C1 | C2 | C7 | 177.7(3) |  | C11 | N1 | C14 | C15 | -179.3(3) |
| O2 | C1 | C2 | C3 | -173.0(3) |  | C11 | N1 | C16 | N2 | 178.7(3) |
| O2 | C1 | C2 | C7 | 2.0(5) |  | C11 | C12 | C13 | C8 | 0.6(5) |
| N1 | C11 | C12 | C13 | 178.2(3) |  | C13 | C8 | C9 | C10 | -0.2(5) |
| N1 | C14 | C15 | N2 | 0.3(4) |  | C14 | N1 | C11 | C10 | -38.6(5) |
| C1 | C2 | C3 | C4 | 173.4(3) |  | C14 | N1 | C11 | C12 | 143.1(3) |
| C1 | C2 | C7 | C6 | -174.1(3) |  | C14 | N1 | C16 | N2 | -1.1(4) |
| C2 | C3 | C4 | C5 | 0.7(5) |  | C15 | N2 | C16 | N1 | 1.3(4) |
| C3 | C2 | C7 | C6 | 1.0(5) |  | C16 | N1 | C11 | C10 | 141.7(3) |
| C3 | C4 | C5 | C6 | 1.1(5) |  | C16 | N1 | C11 | C12 | -36.6(4) |
| C3 | C4 | C5 | C8 | -177.7(3) |  | C16 | N1 | C14 | C15 | 0.5(4) |
| C4 | C5 | C6 | C7 | -1.8(5) |  | C16 | N2 | C15 | C14 | -1.0(4) |
| C4 | C5 | C8 | C9 | 28.6(5) |  |  |  |  |  |  |

13/2-X,+Y,1/2-Z

Table 8 Hydrogen Atom Coordinates (Å×104) and Isotropic Displacement Parameters (Å2×103) for MD\_4168\_BeDa.

| Atom | *x* | *y* | *z* | U(eq) |
| --- | --- | --- | --- | --- |
| H2 | 1725.82 | -4478.64 | 5937.86 | 28 |
| H3 | 5358.25 | 3218.7 | 1673 | 24 |
| H4 | 4596.1 | 1295.14 | 2110.75 | 23 |
| H6 | 4127.72 | 3169.97 | 4487.08 | 26 |
| H7 | 4925.04 | 5064.64 | 4062.08 | 24 |
| H9 | 4785.96 | -524.29 | 3094.09 | 26 |
| H10 | 4097.96 | -2459.19 | 3579.51 | 26 |
| H12 | 2005.98 | -356.17 | 4798.94 | 24 |
| H13 | 2732.02 | 1584.82 | 4339.25 | 24 |
| H14 | 2360.78 | -4156.13 | 3395.03 | 33 |
| H15 | 1666.34 | -5707.3 | 4448.3 | 34 |
| H16 | 2459.39 | -2297.74 | 5925 | 24 |

MD\_4168\_BeDa


# MD\_4168\_BeDa

Table 1 Crystal data and structure refinement for MD\_4168\_BeDa.

| Identification code | MD\_4168\_BeDa |
| Empirical formula | C32H24GdN7O13 |
| Formula weight | 871.83 |
| Temperature/K | 180.05(10) |
| Crystal system | monoclinic |
| Space group | P2/n |
| a/Å | 11.6154(7) |
| b/Å | 10.0797(5) |
| c/Å | 13.9905(7) |
| α/° | 90 |
| β/° | 109.817(7) |
| γ/° | 90 |
| Volume/Å3 | 1541.00(16) |
| Z | 2 |
| ρcalcg/cm3 | 1.879 |
| μ/mm‑1 | 2.238 |
| F(000) | 866.0 |
| Crystal size/mm3 | 0.3 × 0.05 × 0.05 |
| Radiation | Mo Kα (λ = 0.71073) |
| 2Θ range for data collection/° | 3.956 to 50.048 |
| Index ranges | -13 ≤ h ≤ 13, -11 ≤ k ≤ 11, -16 ≤ l ≤ 16 |
| Reflections collected | 5840 |
| Independent reflections | 2712 [Rint = 0.0267, Rsigma = 0.0498] |
| Data/restraints/parameters | 2712/0/241 |
| Goodness-of-fit on F2 | 1.057 |
| Final R indexes [I>=2σ (I)] | R1 = 0.0300, wR2 = 0.0453 |
| Final R indexes [all data] | R1 = 0.0386, wR2 = 0.0476 |
| Largest diff. peak/hole / e Å-3 | 0.49/-0.42 |
