# Supplementary material for: Novel Lanthanide (III) Complexes Derived from an Imidazole–Biphenyl–Carboxylate Ligand: Synthesis, Structure and Luminescence Properties
Source: Molecules. 2021 Nov 17;26(22):6942. doi: 10.3390/molecules26226942 (PMC8625298; doi:10.3390/molecules26226942)
Supplement: Supplementary file 1 [file molecules-26-06942-s001.zip › CRystallografic data/shI_4078_BeDa_tables.html]

shI\_4078\_BeDa


# shI\_4078\_BeDa

Table 1 Crystal data and structure refinement for shI\_4078\_BeDa.

| Identification code | shI\_4078\_BeDa |
| Empirical formula | C32H24N7NdO13 |
| Formula weight | 858.82 |
| Temperature/K | 179.95(10) |
| Crystal system | monoclinic |
| Space group | P2/n |
| a/Å | 11.6264(8) |
| b/Å | 10.1166(6) |
| c/Å | 14.0056(8) |
| α/° | 90 |
| β/° | 109.845(7) |
| γ/° | 90 |
| Volume/Å3 | 1549.51(17) |
| Z | 2 |
| ρcalcg/cm3 | 1.841 |
| μ/mm‑1 | 1.761 |
| F(000) | 858.0 |
| Crystal size/mm3 | 0.25 × 0.1 × 0.05 |
| Radiation | Mo Kα (λ = 0.71073) |
| 2Θ range for data collection/° | 3.952 to 50.052 |
| Index ranges | -13 ≤ h ≤ 13, -12 ≤ k ≤ 10, -10 ≤ l ≤ 16 |
| Reflections collected | 5738 |
| Independent reflections | 2743 [Rint = 0.0401, Rsigma = 0.0678] |
| Data/restraints/parameters | 2743/0/241 |
| Goodness-of-fit on F2 | 1.007 |
| Final R indexes [I>=2σ (I)] | R1 = 0.0365, wR2 = 0.0578 |
| Final R indexes [all data] | R1 = 0.0447, wR2 = 0.0609 |
| Largest diff. peak/hole / e Å-3 | 0.48/-0.76 |

Table 2 Fractional Atomic Coordinates (×104) and Equivalent Isotropic Displacement Parameters (Å2×103) for shI\_4078\_BeDa. Ueq is defined as 1/3 of of the trace of the orthogonalised UIJ tensor.

| Atom | *x* | *y* | *z* | U(eq) |
| --- | --- | --- | --- | --- |
| Nd1 | 7500 | 7586.3(3) | 2500 | 16.05(10) |
| O1 | 6218(2) | 5522(3) | 1851.6(18) | 19.5(7) |
| O2 | 6053(2) | 6501(3) | 3201.8(19) | 20.9(7) |
| O3 | 9567(2) | 8431(3) | 3638(2) | 25.4(7) |
| O4 | 8394(2) | 7688(3) | 4436.6(19) | 22.9(7) |
| O5 | 10167(3) | 8542(3) | 5296(2) | 30.3(8) |
| O6 | 7176(3) | 9881(3) | 3140.0(19) | 23.3(7) |
| O7 | 7500 | 11746(4) | 2500 | 25.3(10) |
| N1 | 2560(3) | -2798(3) | 4548(2) | 17.3(8) |
| N2 | 1935(3) | -4160(3) | 5453(2) | 20.4(8) |
| N3 | 9392(3) | 8231(3) | 4477(3) | 22.0(8) |
| N4 | 7500 | 10532(5) | 2500 | 20.0(12) |
| C1 | 5846(4) | 5515(4) | 2608(3) | 17.8(9) |
| C2 | 5233(3) | 4322(4) | 2826(3) | 15.2(9) |
| C3 | 5107(3) | 3191(4) | 2241(3) | 17.3(9) |
| C4 | 4654(3) | 2038(4) | 2502(3) | 17.0(9) |
| C5 | 4285(3) | 1979(4) | 3359(3) | 15.1(9) |
| C6 | 4378(3) | 3140(4) | 3925(3) | 16.9(9) |
| C7 | 4849(3) | 4282(4) | 3668(3) | 18.3(9) |
| C8 | 3835(3) | 726(4) | 3655(3) | 16.9(9) |
| C9 | 4228(4) | -498(4) | 3434(3) | 21.1(10) |
| C10 | 3812(3) | -1656(4) | 3722(3) | 19.1(10) |
| C11 | 2984(3) | -1591(4) | 4230(3) | 16.5(9) |
| C12 | 2570(4) | -405(4) | 4456(3) | 19.8(10) |
| C13 | 3000(4) | 754(4) | 4180(3) | 19.7(10) |
| C14 | 2304(4) | -3983(4) | 4031(3) | 23.7(10) |
| C15 | 1915(4) | -4833(4) | 4600(3) | 26.2(11) |
| C16 | 2337(3) | -2951(4) | 5417(3) | 18.8(10) |

Table 3 Anisotropic Displacement Parameters (Å2×103) for shI\_4078\_BeDa. The Anisotropic displacement factor exponent takes the form: -2π2[h2a\*2U11+2hka\*b\*U12+…].

| Atom | U11 | U22 | U33 | U23 | U13 | U12 |
| --- | --- | --- | --- | --- | --- | --- |
| Nd1 | 19.67(18) | 12.70(19) | 19.60(17) | 0 | 11.65(13) | 0 |
| O1 | 28.7(18) | 15.5(17) | 21.7(15) | -2.9(12) | 18.3(13) | -4.6(13) |
| O2 | 27.6(18) | 15.5(16) | 25.5(16) | -4.5(13) | 16.9(13) | -4.8(13) |
| O3 | 26.3(18) | 32.1(19) | 22.4(16) | 3.0(14) | 14.1(13) | -1.4(14) |
| O4 | 21.3(17) | 23.5(18) | 27.4(15) | 3.3(13) | 12.9(13) | -3.5(14) |
| O5 | 30.3(19) | 35(2) | 21.0(16) | 5.2(14) | 2.3(14) | 0.9(15) |
| O6 | 35.9(19) | 19.3(17) | 21.6(15) | 2.9(13) | 18.7(14) | 0.0(14) |
| O7 | 35(3) | 14(2) | 29(2) | 0 | 14(2) | 0 |
| N1 | 20(2) | 16(2) | 16.9(17) | 0.6(14) | 8.3(14) | -1.5(15) |
| N2 | 24(2) | 20(2) | 21.7(19) | 8.1(16) | 13.1(15) | -0.8(16) |
| N3 | 27(2) | 15(2) | 25(2) | 4.9(16) | 11.0(18) | 6.3(17) |
| N4 | 20(3) | 18(3) | 21(3) | 0 | 5(2) | 0 |
| C1 | 17(2) | 17(2) | 18(2) | 3.3(18) | 4.8(18) | 5.3(18) |
| C2 | 13(2) | 16(2) | 16(2) | 2.4(17) | 3.5(17) | -1.3(17) |
| C3 | 19(2) | 22(2) | 12(2) | 2.2(17) | 6.9(17) | 1.3(19) |
| C4 | 16(2) | 14(2) | 20(2) | -2.8(17) | 4.6(17) | -2.1(18) |
| C5 | 12(2) | 19(2) | 15(2) | 0.7(17) | 4.6(17) | -1.6(17) |
| C6 | 22(2) | 19(2) | 13(2) | 0.9(17) | 10.1(18) | 0.3(18) |
| C7 | 23(3) | 13(2) | 21(2) | -2.4(18) | 10.9(18) | 1.4(19) |
| C8 | 17(2) | 18(2) | 17(2) | 3.5(18) | 7.6(17) | -1.3(18) |
| C9 | 19(2) | 24(3) | 26(2) | 2.5(19) | 14.8(19) | -1.2(19) |
| C10 | 21(2) | 19(2) | 20(2) | -2.3(19) | 11.2(18) | 1.0(19) |
| C11 | 16(2) | 15(2) | 17(2) | 3.7(18) | 3.9(17) | -3.2(18) |
| C12 | 22(2) | 23(3) | 18(2) | -0.5(18) | 12.1(18) | -3.6(19) |
| C13 | 24(3) | 16(2) | 21(2) | -1.8(18) | 9.5(19) | 1.7(19) |
| C14 | 33(3) | 22(3) | 19(2) | -6.3(19) | 13(2) | -7(2) |
| C15 | 32(3) | 23(3) | 24(2) | -4(2) | 9(2) | -7(2) |
| C16 | 19(2) | 19(3) | 17(2) | -0.2(17) | 3.9(18) | 0.8(18) |

Table 4 Bond Lengths for shI\_4078\_BeDa.

| Atom | Atom | Length/Å |  | Atom | Atom | Length/Å |
| --- | --- | --- | --- | --- | --- | --- |
| Nd1 | O1 | 2.548(3) |  | N1 | C14 | 1.380(5) |
| Nd1 | O11 | 2.548(3) |  | N1 | C16 | 1.337(5) |
| Nd1 | O2 | 2.475(3) |  | N2 | C15 | 1.368(5) |
| Nd1 | O21 | 2.475(3) |  | N2 | C16 | 1.316(5) |
| Nd1 | O31 | 2.539(3) |  | C1 | C2 | 1.485(5) |
| Nd1 | O3 | 2.539(3) |  | C2 | C3 | 1.385(5) |
| Nd1 | O4 | 2.556(2) |  | C2 | C7 | 1.396(5) |
| Nd1 | O41 | 2.556(2) |  | C3 | C4 | 1.379(5) |
| Nd1 | O61 | 2.562(3) |  | C4 | C5 | 1.406(5) |
| Nd1 | O6 | 2.562(3) |  | C5 | C6 | 1.401(5) |
| Nd1 | C11 | 2.883(4) |  | C5 | C8 | 1.483(5) |
| Nd1 | C1 | 2.883(4) |  | C6 | C7 | 1.377(5) |
| O1 | C1 | 1.273(5) |  | C8 | C9 | 1.391(5) |
| O2 | C1 | 1.269(4) |  | C8 | C13 | 1.404(5) |
| O3 | N3 | 1.274(4) |  | C9 | C10 | 1.379(5) |
| O4 | N3 | 1.268(4) |  | C10 | C11 | 1.379(5) |
| O5 | N3 | 1.235(4) |  | C11 | C12 | 1.369(5) |
| O6 | N4 | 1.268(3) |  | C12 | C13 | 1.380(5) |
| O7 | N4 | 1.228(5) |  | C14 | C15 | 1.351(5) |
| N1 | C11 | 1.442(5) |  |  |  |  |

13/2-X,+Y,1/2-Z

Table 5 Bond Angles for shI\_4078\_BeDa.

| Atom | Atom | Atom | Angle/˚ |  | Atom | Atom | Atom | Angle/˚ |
| --- | --- | --- | --- | --- | --- | --- | --- | --- |
| O1 | Nd1 | O11 | 69.87(12) |  | O41 | Nd1 | C1 | 92.68(10) |
| O1 | Nd1 | O4 | 112.90(8) |  | O4 | Nd1 | C1 | 90.67(10) |
| O11 | Nd1 | O4 | 71.14(8) |  | O6 | Nd1 | O61 | 50.05(12) |
| O1 | Nd1 | O41 | 71.14(8) |  | O6 | Nd1 | C1 | 117.42(10) |
| O11 | Nd1 | O41 | 112.90(8) |  | O61 | Nd1 | C1 | 148.99(10) |
| O1 | Nd1 | O61 | 139.11(8) |  | O6 | Nd1 | C11 | 148.99(10) |
| O11 | Nd1 | O61 | 136.87(9) |  | O61 | Nd1 | C11 | 117.42(10) |
| O11 | Nd1 | O6 | 139.11(8) |  | C11 | Nd1 | C1 | 86.72(16) |
| O1 | Nd1 | O6 | 136.87(9) |  | C1 | O1 | Nd1 | 91.8(2) |
| O11 | Nd1 | C1 | 72.86(9) |  | C1 | O2 | Nd1 | 95.2(2) |
| O11 | Nd1 | C11 | 26.19(9) |  | N3 | O3 | Nd1 | 96.5(2) |
| O1 | Nd1 | C11 | 72.86(9) |  | N3 | O4 | Nd1 | 95.8(2) |
| O1 | Nd1 | C1 | 26.19(9) |  | N4 | O6 | Nd1 | 96.2(2) |
| O2 | Nd1 | O11 | 83.65(9) |  | C14 | N1 | C11 | 127.5(3) |
| O21 | Nd1 | O1 | 83.65(9) |  | C16 | N1 | C11 | 124.9(3) |
| O21 | Nd1 | O11 | 51.94(8) |  | C16 | N1 | C14 | 107.7(4) |
| O2 | Nd1 | O1 | 51.94(8) |  | C16 | N2 | C15 | 109.3(3) |
| O2 | Nd1 | O21 | 127.35(12) |  | O3 | N3 | Nd1 | 58.26(18) |
| O21 | Nd1 | O3 | 76.84(9) |  | O4 | N3 | Nd1 | 59.02(18) |
| O21 | Nd1 | O31 | 121.74(8) |  | O4 | N3 | O3 | 117.2(3) |
| O2 | Nd1 | O3 | 121.74(8) |  | O5 | N3 | Nd1 | 177.9(3) |
| O2 | Nd1 | O31 | 76.84(9) |  | O5 | N3 | O3 | 121.5(4) |
| O2 | Nd1 | O41 | 110.67(8) |  | O5 | N3 | O4 | 121.3(3) |
| O21 | Nd1 | O4 | 110.67(8) |  | O61 | N4 | Nd1 | 58.7(2) |
| O2 | Nd1 | O4 | 71.50(8) |  | O6 | N4 | Nd1 | 58.7(2) |
| O21 | Nd1 | O41 | 71.50(8) |  | O61 | N4 | O6 | 117.5(4) |
| O21 | Nd1 | O61 | 93.57(9) |  | O7 | N4 | Nd1 | 180.0 |
| O2 | Nd1 | O61 | 137.85(9) |  | O7 | N4 | O61 | 121.3(2) |
| O2 | Nd1 | O6 | 93.57(9) |  | O7 | N4 | O6 | 121.3(2) |
| O21 | Nd1 | O6 | 137.85(9) |  | O1 | C1 | Nd1 | 62.1(2) |
| O2 | Nd1 | C1 | 26.00(9) |  | O1 | C1 | C2 | 119.9(4) |
| O2 | Nd1 | C11 | 104.71(11) |  | O2 | C1 | Nd1 | 58.8(2) |
| O21 | Nd1 | C11 | 26.00(9) |  | O2 | C1 | O1 | 119.9(4) |
| O21 | Nd1 | C1 | 104.71(11) |  | O2 | C1 | C2 | 120.1(4) |
| O3 | Nd1 | O11 | 74.77(9) |  | C2 | C1 | Nd1 | 167.6(3) |
| O31 | Nd1 | O1 | 74.77(9) |  | C3 | C2 | C1 | 120.8(4) |
| O31 | Nd1 | O11 | 144.52(9) |  | C3 | C2 | C7 | 118.4(4) |
| O3 | Nd1 | O1 | 144.52(9) |  | C7 | C2 | C1 | 120.7(4) |
| O3 | Nd1 | O31 | 140.68(13) |  | C4 | C3 | C2 | 121.0(4) |
| O31 | Nd1 | O4 | 127.59(9) |  | C3 | C4 | C5 | 121.1(4) |
| O31 | Nd1 | O41 | 50.42(9) |  | C4 | C5 | C8 | 120.9(4) |
| O3 | Nd1 | O4 | 50.43(9) |  | C6 | C5 | C4 | 117.4(4) |
| O3 | Nd1 | O41 | 127.59(9) |  | C6 | C5 | C8 | 121.7(3) |
| O3 | Nd1 | O61 | 72.21(9) |  | C7 | C6 | C5 | 121.1(4) |
| O31 | Nd1 | O61 | 72.29(9) |  | C6 | C7 | C2 | 121.0(4) |
| O3 | Nd1 | O6 | 72.29(9) |  | C9 | C8 | C5 | 121.7(3) |
| O31 | Nd1 | O6 | 72.21(9) |  | C9 | C8 | C13 | 118.2(4) |
| O3 | Nd1 | C11 | 76.79(10) |  | C13 | C8 | C5 | 120.2(4) |
| O3 | Nd1 | C1 | 135.86(9) |  | C10 | C9 | C8 | 121.2(4) |
| O31 | Nd1 | C1 | 76.79(10) |  | C9 | C10 | C11 | 119.0(4) |
| O31 | Nd1 | C11 | 135.86(9) |  | C10 | C11 | N1 | 119.4(4) |
| O41 | Nd1 | O4 | 175.39(13) |  | C12 | C11 | N1 | 119.1(3) |
| O4 | Nd1 | O6 | 69.36(8) |  | C12 | C11 | C10 | 121.5(4) |
| O41 | Nd1 | O6 | 106.24(8) |  | C11 | C12 | C13 | 119.4(4) |
| O41 | Nd1 | O61 | 69.36(8) |  | C12 | C13 | C8 | 120.7(4) |
| O4 | Nd1 | O61 | 106.24(8) |  | C15 | C14 | N1 | 107.4(4) |
| O41 | Nd1 | C11 | 90.67(10) |  | C14 | C15 | N2 | 106.8(4) |
| O4 | Nd1 | C11 | 92.68(10) |  | N2 | C16 | N1 | 108.9(4) |

13/2-X,+Y,1/2-Z

Table 6 Torsion Angles for shI\_4078\_BeDa.

| A | B | C | D | Angle/˚ |  | A | B | C | D | Angle/˚ |
| --- | --- | --- | --- | --- | --- | --- | --- | --- | --- | --- |
| Nd1 | O1 | C1 | O2 | 10.6(4) |  | C4 | C5 | C8 | C13 | -151.9(4) |
| Nd1 | O1 | C1 | C2 | -166.0(3) |  | C5 | C6 | C7 | C2 | 0.9(6) |
| Nd1 | O2 | C1 | O1 | -10.9(4) |  | C5 | C8 | C9 | C10 | 179.4(3) |
| Nd1 | O2 | C1 | C2 | 165.7(3) |  | C5 | C8 | C13 | C12 | 179.7(3) |
| Nd1 | O3 | N3 | O4 | 3.1(3) |  | C6 | C5 | C8 | C9 | -150.3(4) |
| Nd1 | O3 | N3 | O5 | -177.6(3) |  | C6 | C5 | C8 | C13 | 29.2(5) |
| Nd1 | O4 | N3 | O3 | -3.1(3) |  | C7 | C2 | C3 | C4 | -2.4(5) |
| Nd1 | O4 | N3 | O5 | 177.6(3) |  | C8 | C5 | C6 | C7 | 177.0(3) |
| Nd1 | O6 | N4 | O61 | 0.002(2) |  | C8 | C9 | C10 | C11 | 0.6(6) |
| Nd1 | O6 | N4 | O7 | 180.000(1) |  | C9 | C8 | C13 | C12 | -0.9(6) |
| Nd1 | C1 | C2 | C3 | -92.6(13) |  | C9 | C10 | C11 | N1 | -179.0(3) |
| Nd1 | C1 | C2 | C7 | 82.8(14) |  | C9 | C10 | C11 | C12 | -0.1(6) |
| O1 | C1 | C2 | C3 | 2.8(6) |  | C10 | C11 | C12 | C13 | -0.8(6) |
| O1 | C1 | C2 | C7 | 178.3(4) |  | C11 | N1 | C14 | C15 | -179.0(3) |
| O2 | C1 | C2 | C3 | -173.8(3) |  | C11 | N1 | C16 | N2 | 178.5(3) |
| O2 | C1 | C2 | C7 | 1.7(6) |  | C11 | C12 | C13 | C8 | 1.3(6) |
| N1 | C11 | C12 | C13 | 178.1(3) |  | C13 | C8 | C9 | C10 | -0.1(6) |
| N1 | C14 | C15 | N2 | 0.1(5) |  | C14 | N1 | C11 | C10 | -37.7(6) |
| C1 | C2 | C3 | C4 | 173.2(3) |  | C14 | N1 | C11 | C12 | 143.4(4) |
| C1 | C2 | C7 | C6 | -174.3(3) |  | C14 | N1 | C16 | N2 | -1.2(4) |
| C2 | C3 | C4 | C5 | 1.3(6) |  | C15 | N2 | C16 | N1 | 1.3(4) |
| C3 | C2 | C7 | C6 | 1.3(6) |  | C16 | N1 | C11 | C10 | 142.7(4) |
| C3 | C4 | C5 | C6 | 0.9(5) |  | C16 | N1 | C11 | C12 | -36.2(5) |
| C3 | C4 | C5 | C8 | -178.1(3) |  | C16 | N1 | C14 | C15 | 0.7(5) |
| C4 | C5 | C6 | C7 | -1.9(5) |  | C16 | N2 | C15 | C14 | -0.8(5) |
| C4 | C5 | C8 | C9 | 28.6(5) |  |  |  |  |  |  |

13/2-X,+Y,1/2-Z

Table 7 Hydrogen Atom Coordinates (Å×104) and Isotropic Displacement Parameters (Å2×103) for shI\_4078\_BeDa.

| Atom | *x* | *y* | *z* | U(eq) |
| --- | --- | --- | --- | --- |
| H2 | 1719.61 | -4477.45 | 5936.5 | 24 |
| H3 | 5329.9 | 3210.22 | 1663.5 | 21 |
| H4 | 4592.83 | 1286.65 | 2105.45 | 20 |
| H6 | 4117.99 | 3139.87 | 4483.77 | 20 |
| H7 | 4912.18 | 5036.8 | 4061.23 | 22 |
| H9 | 4780.25 | -536.28 | 3085.72 | 25 |
| H10 | 4086.22 | -2469.74 | 3575.8 | 23 |
| H12 | 2003.98 | -381.12 | 4791.18 | 24 |
| H13 | 2733.13 | 1561.31 | 4344.71 | 24 |
| H14 | 2385.28 | -4162.91 | 3405.53 | 28 |
| H15 | 1678.87 | -5706.67 | 4442.35 | 31 |
| H16 | 2447.98 | -2308.57 | 5915.48 | 23 |

shI\_4078\_BeDa


# shI\_4078\_BeDa

Table 1 Crystal data and structure refinement for shI\_4078\_BeDa.

| Identification code | shI\_4078\_BeDa |
| Empirical formula | C32H24N7NdO13 |
| Formula weight | 858.82 |
| Temperature/K | 179.95(10) |
| Crystal system | monoclinic |
| Space group | P2/n |
| a/Å | 11.6264(8) |
| b/Å | 10.1166(6) |
| c/Å | 14.0056(8) |
| α/° | 90 |
| β/° | 109.845(7) |
| γ/° | 90 |
| Volume/Å3 | 1549.51(17) |
| Z | 2 |
| ρcalcg/cm3 | 1.841 |
| μ/mm‑1 | 1.761 |
| F(000) | 858.0 |
| Crystal size/mm3 | 0.25 × 0.1 × 0.05 |
| Radiation | Mo Kα (λ = 0.71073) |
| 2Θ range for data collection/° | 3.952 to 50.052 |
| Index ranges | -13 ≤ h ≤ 13, -12 ≤ k ≤ 10, -10 ≤ l ≤ 16 |
| Reflections collected | 5738 |
| Independent reflections | 2743 [Rint = 0.0401, Rsigma = 0.0678] |
| Data/restraints/parameters | 2743/0/241 |
| Goodness-of-fit on F2 | 1.007 |
| Final R indexes [I>=2σ (I)] | R1 = 0.0365, wR2 = 0.0578 |
| Final R indexes [all data] | R1 = 0.0447, wR2 = 0.0609 |
| Largest diff. peak/hole / e Å-3 | 0.48/-0.76 |
