# Supplementary material for: Novel Lanthanide (III) Complexes Derived from an Imidazole–Biphenyl–Carboxylate Ligand: Synthesis, Structure and Luminescence Properties
Source: Molecules. 2021 Nov 17;26(22):6942. doi: 10.3390/molecules26226942 (PMC8625298; doi:10.3390/molecules26226942)
Supplement: Supplementary file 1 [file molecules-26-06942-s001.zip › CRystallografic data/shI_4082_BeDa_tables.html]

shI\_4082\_BeDa


# shI\_4082\_BeDa

Table 1 Crystal data and structure refinement for shI\_4082\_BeDa.

| Identification code | shI\_4082\_BeDa |
| Empirical formula | C32H24LaN7O13 |
| Formula weight | 853.49 |
| Temperature/K | 293(2) |
| Crystal system | monoclinic |
| Space group | P2/n |
| a/Å | 11.7434(7) |
| b/Å | 10.0978(5) |
| c/Å | 14.0667(9) |
| α/° | 90 |
| β/° | 108.771(7) |
| γ/° | 90 |
| Volume/Å3 | 1579.36(17) |
| Z | 2 |
| ρcalcg/cm3 | 1.795 |
| μ/mm‑1 | 1.436 |
| F(000) | 852.0 |
| Crystal size/mm3 | 0.25 × 0.05 × 0.02 |
| Radiation | Mo Kα (λ = 0.71073) |
| 2Θ range for data collection/° | 4.034 to 50.054 |
| Index ranges | -13 ≤ h ≤ 12, -12 ≤ k ≤ 11, -16 ≤ l ≤ 12 |
| Reflections collected | 5552 |
| Independent reflections | 2786 [Rint = 0.0333, Rsigma = 0.0569] |
| Data/restraints/parameters | 2786/0/241 |
| Goodness-of-fit on F2 | 0.976 |
| Final R indexes [I>=2σ (I)] | R1 = 0.0326, wR2 = 0.0460 |
| Final R indexes [all data] | R1 = 0.0433, wR2 = 0.0483 |
| Largest diff. peak/hole / e Å-3 | 0.51/-0.37 |

Table 2 Fractional Atomic Coordinates (×104) and Equivalent Isotropic Displacement Parameters (Å2×103) for shI\_4082\_BeDa. Ueq is defined as 1/3 of of the trace of the orthogonalised UIJ tensor.

| Atom | *x* | *y* | *z* | U(eq) |
| --- | --- | --- | --- | --- |
| La1 | 7500 | 7612.5(3) | 2500 | 28.37(9) |
| O1 | 6220.3(18) | 5493(2) | 1865.6(16) | 37.5(6) |
| O2 | 6037.8(18) | 6466(2) | 3205.3(16) | 37.8(6) |
| O4 | 8413.0(19) | 7717(2) | 4448.7(16) | 42.3(6) |
| O3 | 9579.8(19) | 8452(2) | 3653.0(18) | 45.1(6) |
| O5 | 10167(2) | 8511(3) | 5282.3(19) | 60.3(8) |
| O6 | 7180.4(19) | 9975(2) | 3143.4(16) | 41.4(6) |
| O7 | 7500 | 11825(3) | 2500 | 46.9(9) |
| N1 | 2560(2) | -2810(3) | 4549.1(19) | 32.2(6) |
| N2 | 1942(2) | -4151(3) | 5454(2) | 40.3(7) |
| N3 | 9411(3) | 8238(3) | 4480(2) | 38.9(7) |
| N4 | 7500 | 10607(4) | 2500 | 34.0(10) |
| C1 | 5846(3) | 5493(3) | 2615(3) | 31.6(8) |
| C2 | 5230(3) | 4302(3) | 2833(2) | 27.9(8) |
| C3 | 5094(3) | 3176(3) | 2250(2) | 31.9(8) |
| C4 | 4635(3) | 2024(3) | 2506(2) | 32.4(8) |
| C5 | 4275(2) | 1967(3) | 3359(2) | 30.0(8) |
| C6 | 4384(3) | 3116(3) | 3930(2) | 35.4(8) |
| C7 | 4862(3) | 4258(3) | 3678(2) | 34.8(8) |
| C8 | 3830(3) | 713(3) | 3657(2) | 30.7(8) |
| C9 | 4211(3) | -506(3) | 3435(3) | 39.9(9) |
| C10 | 3804(3) | -1668(3) | 3718(2) | 38.4(9) |
| C11 | 2983(3) | -1610(3) | 4228(2) | 31.5(8) |
| C12 | 2574(3) | -420(3) | 4457(3) | 35.7(9) |
| C13 | 3004(3) | 731(3) | 4176(2) | 36.0(8) |
| C14 | 2284(3) | -3980(4) | 4045(3) | 46.0(10) |
| C15 | 1901(3) | -4819(4) | 4610(3) | 48.2(10) |
| C16 | 2348(3) | -2949(3) | 5414(2) | 36.6(8) |

Table 3 Anisotropic Displacement Parameters (Å2×103) for shI\_4082\_BeDa. The Anisotropic displacement factor exponent takes the form: -2π2[h2a\*2U11+2hka\*b\*U12+…].

| Atom | U11 | U22 | U33 | U23 | U13 | U12 |
| --- | --- | --- | --- | --- | --- | --- |
| La1 | 34.84(16) | 21.96(15) | 34.87(16) | 0 | 20.36(11) | 0 |
| O1 | 50.8(15) | 31.4(14) | 42.2(16) | -8.1(11) | 31.5(12) | -8.0(11) |
| O2 | 52.0(14) | 27.7(14) | 45.7(16) | -8.1(12) | 32.4(12) | -9.1(11) |
| O4 | 44.2(13) | 42.5(16) | 48.2(15) | 3.2(13) | 26.1(11) | -1.6(12) |
| O3 | 47.7(14) | 55.3(17) | 40.3(16) | -2.5(13) | 25.4(12) | -12.2(13) |
| O5 | 54.1(17) | 76(2) | 41.0(17) | 6.6(15) | 1.9(13) | -2.4(15) |
| O6 | 61.5(16) | 34.3(14) | 39.1(15) | 2.8(12) | 31.0(12) | 3.1(12) |
| O7 | 65(2) | 27(2) | 51(2) | 0 | 21.7(18) | 0 |
| N1 | 39.6(15) | 28.9(17) | 31.8(16) | 2.9(14) | 16.7(12) | -2.9(13) |
| N2 | 45.2(18) | 38.7(19) | 45(2) | 12.9(16) | 25.8(15) | -1.1(15) |
| N3 | 45.3(19) | 30.8(17) | 42(2) | 4.0(15) | 16.4(16) | 8.9(15) |
| N4 | 42(3) | 32(3) | 28(3) | 0 | 11.8(19) | 0 |
| C1 | 35(2) | 29(2) | 34(2) | -1.5(16) | 14.8(16) | 0.5(15) |
| C2 | 28.2(18) | 27.5(19) | 30(2) | -1.0(16) | 11.6(14) | -2.2(15) |
| C3 | 37.4(19) | 35(2) | 29(2) | 0.9(16) | 18.1(15) | -0.5(16) |
| C4 | 37.8(19) | 26.6(19) | 37(2) | -2.7(16) | 17.2(16) | -3.2(15) |
| C5 | 26.3(17) | 32(2) | 32(2) | 3.9(16) | 10.1(15) | -0.3(14) |
| C6 | 42(2) | 36(2) | 35(2) | -0.5(17) | 20.5(16) | -2.7(17) |
| C7 | 38(2) | 30(2) | 39(2) | -3.8(17) | 17.2(16) | -3.4(16) |
| C8 | 27.8(19) | 32(2) | 35(2) | 2.8(17) | 14.4(15) | -2.4(15) |
| C9 | 43(2) | 39(2) | 51(3) | 7.0(18) | 32.8(18) | 1.8(17) |
| C10 | 47(2) | 30(2) | 48(2) | -0.8(18) | 28.4(18) | 1.3(17) |
| C11 | 36.7(19) | 29(2) | 31(2) | 2.7(16) | 14.1(16) | -3.8(16) |
| C12 | 39(2) | 35(2) | 41(2) | 0.8(17) | 24.2(16) | -2.0(17) |
| C13 | 42(2) | 30(2) | 44(2) | 1.0(17) | 24.8(17) | 5.7(17) |
| C14 | 69(3) | 36(2) | 37(2) | -10.1(19) | 23.6(19) | -13(2) |
| C15 | 66(3) | 34(2) | 46(3) | -3(2) | 20(2) | -11(2) |
| C16 | 43(2) | 37(2) | 34(2) | 3.9(17) | 18.4(16) | 1.3(17) |

Table 4 Bond Lengths for shI\_4082\_BeDa.

| Atom | Atom | Length/Å |  | Atom | Atom | Length/Å |
| --- | --- | --- | --- | --- | --- | --- |
| La1 | O11 | 2.602(2) |  | N1 | C14 | 1.363(4) |
| La1 | O1 | 2.602(2) |  | N1 | C16 | 1.325(4) |
| La1 | O2 | 2.524(2) |  | N2 | C15 | 1.352(4) |
| La1 | O21 | 2.524(2) |  | N2 | C16 | 1.312(4) |
| La1 | O41 | 2.605(2) |  | C1 | C2 | 1.485(4) |
| La1 | O4 | 2.605(2) |  | C2 | C3 | 1.381(4) |
| La1 | O3 | 2.601(2) |  | C2 | C7 | 1.390(4) |
| La1 | O31 | 2.601(2) |  | C3 | C4 | 1.378(4) |
| La1 | O6 | 2.621(2) |  | C4 | C5 | 1.396(4) |
| La1 | O61 | 2.621(2) |  | C5 | C6 | 1.393(4) |
| La1 | C11 | 2.930(3) |  | C5 | C8 | 1.480(4) |
| La1 | C1 | 2.930(3) |  | C6 | C7 | 1.378(4) |
| O1 | C1 | 1.266(4) |  | C8 | C9 | 1.379(4) |
| O2 | C1 | 1.259(4) |  | C8 | C13 | 1.390(4) |
| O4 | N3 | 1.272(3) |  | C9 | C10 | 1.374(4) |
| O3 | N3 | 1.260(3) |  | C10 | C11 | 1.375(4) |
| O5 | N3 | 1.221(3) |  | C11 | C12 | 1.371(4) |
| O6 | N4 | 1.260(3) |  | C12 | C13 | 1.375(4) |
| O7 | N4 | 1.229(5) |  | C14 | C15 | 1.335(5) |
| N1 | C11 | 1.436(4) |  |  |  |  |

13/2-X,+Y,1/2-Z

Table 5 Bond Angles for shI\_4082\_BeDa.

| Atom | Atom | Atom | Angle/˚ |  | Atom | Atom | Atom | Angle/˚ |
| --- | --- | --- | --- | --- | --- | --- | --- | --- |
| O11 | La1 | O1 | 69.35(9) |  | O3 | La1 | C11 | 76.49(8) |
| O11 | La1 | O41 | 113.03(7) |  | O31 | La1 | C11 | 135.22(8) |
| O1 | La1 | O41 | 71.08(7) |  | O6 | La1 | O61 | 48.95(10) |
| O1 | La1 | O4 | 113.03(7) |  | O6 | La1 | C1 | 118.34(8) |
| O11 | La1 | O4 | 71.08(7) |  | O61 | La1 | C11 | 118.34(8) |
| O1 | La1 | O61 | 139.57(7) |  | O61 | La1 | C1 | 148.79(8) |
| O1 | La1 | O6 | 137.38(6) |  | O6 | La1 | C11 | 148.79(8) |
| O11 | La1 | O61 | 137.38(6) |  | C11 | La1 | C1 | 86.13(13) |
| O11 | La1 | O6 | 139.57(7) |  | C1 | O1 | La1 | 91.82(18) |
| O11 | La1 | C1 | 72.56(7) |  | C1 | O2 | La1 | 95.67(19) |
| O1 | La1 | C1 | 25.59(7) |  | N3 | O4 | La1 | 96.67(18) |
| O11 | La1 | C11 | 25.59(7) |  | N3 | O3 | La1 | 97.17(18) |
| O1 | La1 | C11 | 72.56(7) |  | N4 | O6 | La1 | 96.0(2) |
| O2 | La1 | O1 | 50.70(7) |  | C14 | N1 | C11 | 128.0(3) |
| O2 | La1 | O11 | 83.04(7) |  | C16 | N1 | C11 | 124.4(3) |
| O21 | La1 | O1 | 83.04(7) |  | C16 | N1 | C14 | 107.6(3) |
| O21 | La1 | O11 | 50.70(7) |  | C16 | N2 | C15 | 109.5(3) |
| O21 | La1 | O2 | 125.37(10) |  | O4 | N3 | La1 | 58.67(15) |
| O21 | La1 | O4 | 109.31(7) |  | O3 | N3 | La1 | 58.44(15) |
| O21 | La1 | O41 | 72.95(7) |  | O3 | N3 | O4 | 117.1(3) |
| O2 | La1 | O41 | 109.31(7) |  | O5 | N3 | La1 | 178.6(2) |
| O2 | La1 | O4 | 72.94(7) |  | O5 | N3 | O4 | 120.8(3) |
| O2 | La1 | O3 | 121.70(7) |  | O5 | N3 | O3 | 122.1(3) |
| O21 | La1 | O31 | 121.70(7) |  | O61 | N4 | La1 | 59.5(2) |
| O21 | La1 | O3 | 76.91(7) |  | O6 | N4 | La1 | 59.5(2) |
| O2 | La1 | O31 | 76.91(7) |  | O61 | N4 | O6 | 119.1(4) |
| O21 | La1 | O6 | 138.12(7) |  | O7 | N4 | La1 | 180.0 |
| O2 | La1 | O6 | 95.20(7) |  | O7 | N4 | O61 | 120.5(2) |
| O21 | La1 | O61 | 95.21(7) |  | O7 | N4 | O6 | 120.5(2) |
| O2 | La1 | O61 | 138.12(7) |  | O1 | C1 | La1 | 62.59(16) |
| O2 | La1 | C11 | 103.51(8) |  | O1 | C1 | C2 | 119.7(3) |
| O21 | La1 | C11 | 25.31(7) |  | O2 | C1 | La1 | 59.02(16) |
| O21 | La1 | C1 | 103.51(8) |  | O2 | C1 | O1 | 120.8(3) |
| O2 | La1 | C1 | 25.31(7) |  | O2 | C1 | C2 | 119.4(3) |
| O4 | La1 | O41 | 175.34(11) |  | C2 | C1 | La1 | 168.1(2) |
| O41 | La1 | O61 | 69.86(7) |  | C3 | C2 | C1 | 121.0(3) |
| O41 | La1 | O6 | 105.68(7) |  | C3 | C2 | C7 | 118.3(3) |
| O4 | La1 | O61 | 105.68(7) |  | C7 | C2 | C1 | 120.6(3) |
| O4 | La1 | O6 | 69.86(7) |  | C4 | C3 | C2 | 121.3(3) |
| O41 | La1 | C11 | 91.56(8) |  | C3 | C4 | C5 | 120.8(3) |
| O4 | La1 | C1 | 91.56(8) |  | C4 | C5 | C8 | 120.7(3) |
| O41 | La1 | C1 | 91.85(8) |  | C6 | C5 | C4 | 117.6(3) |
| O4 | La1 | C11 | 91.85(8) |  | C6 | C5 | C8 | 121.7(3) |
| O3 | La1 | O1 | 143.58(7) |  | C7 | C6 | C5 | 121.3(3) |
| O3 | La1 | O11 | 74.42(7) |  | C6 | C7 | C2 | 120.7(3) |
| O31 | La1 | O11 | 143.58(7) |  | C9 | C8 | C5 | 122.0(3) |
| O31 | La1 | O1 | 74.42(7) |  | C9 | C8 | C13 | 117.6(3) |
| O31 | La1 | O41 | 49.04(7) |  | C13 | C8 | C5 | 120.4(3) |
| O3 | La1 | O41 | 128.98(7) |  | C10 | C9 | C8 | 121.9(3) |
| O3 | La1 | O4 | 49.04(7) |  | C9 | C10 | C11 | 118.9(3) |
| O31 | La1 | O4 | 128.98(7) |  | C10 | C11 | N1 | 120.0(3) |
| O31 | La1 | O3 | 141.95(11) |  | C12 | C11 | N1 | 118.8(3) |
| O3 | La1 | O6 | 72.43(7) |  | C12 | C11 | C10 | 121.1(3) |
| O3 | La1 | O61 | 73.06(7) |  | C11 | C12 | C13 | 119.0(3) |
| O31 | La1 | O6 | 73.06(7) |  | C12 | C13 | C8 | 121.5(3) |
| O31 | La1 | O61 | 72.43(7) |  | C15 | C14 | N1 | 108.0(3) |
| O31 | La1 | C1 | 76.49(8) |  | C14 | C15 | N2 | 106.4(3) |
| O3 | La1 | C1 | 135.23(8) |  | N2 | C16 | N1 | 108.4(3) |

13/2-X,+Y,1/2-Z

Table 6 Hydrogen Bonds for shI\_4082\_BeDa.

| D | H | A | d(D-H)/Å | d(H-A)/Å | d(D-A)/Å | D-H-A/° |
| --- | --- | --- | --- | --- | --- | --- |
| N2 | H2 | O11 | 0.86 | 1.91 | 2.754(3) | 168.2 |
| C15 | H15 | O42 | 0.93 | 2.58 | 3.282(4) | 132.6 |
| C16 | H16 | O73 | 0.93 | 2.28 | 3.098(4) | 146.6 |

1-1/2+X,-Y,1/2+Z; 21-X,-Y,1-Z; 31-X,1-Y,1-Z

Table 7 Torsion Angles for shI\_4082\_BeDa.

| A | B | C | D | Angle/˚ |  | A | B | C | D | Angle/˚ |
| --- | --- | --- | --- | --- | --- | --- | --- | --- | --- | --- |
| La1 | O1 | C1 | O2 | 10.0(3) |  | C4 | C5 | C8 | C13 | -151.0(3) |
| La1 | O1 | C1 | C2 | -166.7(3) |  | C5 | C6 | C7 | C2 | 1.2(5) |
| La1 | O2 | C1 | O1 | -10.4(3) |  | C5 | C8 | C9 | C10 | 179.6(3) |
| La1 | O2 | C1 | C2 | 166.4(2) |  | C5 | C8 | C13 | C12 | 179.7(3) |
| La1 | O4 | N3 | O3 | -1.6(3) |  | C6 | C5 | C8 | C9 | -149.0(3) |
| La1 | O4 | N3 | O5 | 178.5(3) |  | C6 | C5 | C8 | C13 | 30.9(5) |
| La1 | O3 | N3 | O4 | 1.6(3) |  | C7 | C2 | C3 | C4 | -1.6(5) |
| La1 | O3 | N3 | O5 | -178.5(3) |  | C8 | C5 | C6 | C7 | 176.3(3) |
| La1 | O6 | N4 | O61 | 0.000(2) |  | C8 | C9 | C10 | C11 | 0.6(5) |
| La1 | O6 | N4 | O7 | 180.000(1) |  | C9 | C8 | C13 | C12 | -0.3(5) |
| La1 | C1 | C2 | C3 | -95.5(11) |  | C9 | C10 | C11 | N1 | -178.8(3) |
| La1 | C1 | C2 | C7 | 79.3(12) |  | C9 | C10 | C11 | C12 | -0.1(5) |
| O1 | C1 | C2 | C3 | 2.2(5) |  | C10 | C11 | C12 | C13 | -0.6(5) |
| O1 | C1 | C2 | C7 | 177.0(3) |  | C11 | N1 | C14 | C15 | -178.7(3) |
| O2 | C1 | C2 | C3 | -174.6(3) |  | C11 | N1 | C16 | N2 | 178.3(3) |
| O2 | C1 | C2 | C7 | 0.2(5) |  | C11 | C12 | C13 | C8 | 0.8(5) |
| N1 | C11 | C12 | C13 | 178.1(3) |  | C13 | C8 | C9 | C10 | -0.4(5) |
| N1 | C14 | C15 | N2 | 0.2(4) |  | C14 | N1 | C11 | C10 | -39.4(5) |
| C1 | C2 | C3 | C4 | 173.3(3) |  | C14 | N1 | C11 | C12 | 141.9(3) |
| C1 | C2 | C7 | C6 | -174.4(3) |  | C14 | N1 | C16 | N2 | -0.7(4) |
| C2 | C3 | C4 | C5 | 1.0(5) |  | C15 | N2 | C16 | N1 | 0.8(4) |
| C3 | C2 | C7 | C6 | 0.5(5) |  | C16 | N1 | C11 | C10 | 141.8(3) |
| C3 | C4 | C5 | C6 | 0.7(4) |  | C16 | N1 | C11 | C12 | -36.9(5) |
| C3 | C4 | C5 | C8 | -177.4(3) |  | C16 | N1 | C14 | C15 | 0.3(4) |
| C4 | C5 | C6 | C7 | -1.8(5) |  | C16 | N2 | C15 | C14 | -0.7(4) |
| C4 | C5 | C8 | C9 | 29.0(5) |  |  |  |  |  |  |

13/2-X,+Y,1/2-Z

Table 8 Hydrogen Atom Coordinates (Å×104) and Isotropic Displacement Parameters (Å2×103) for shI\_4082\_BeDa.

| Atom | *x* | *y* | *z* | U(eq) |
| --- | --- | --- | --- | --- |
| H2 | 1733.94 | -4465.8 | 5941.35 | 48 |
| H3 | 5317.1 | 3195.95 | 1672.86 | 38 |
| H4 | 4564.08 | 1276.11 | 2105.7 | 39 |
| H6 | 4129.98 | 3112.75 | 4491.31 | 42 |
| H7 | 4939.26 | 5008.06 | 4077.87 | 42 |
| H9 | 4759.92 | -541.59 | 3084.15 | 48 |
| H10 | 4077.74 | -2479.78 | 3566.45 | 46 |
| H12 | 2013.58 | -391.64 | 4796.72 | 43 |
| H13 | 2735.67 | 1540.7 | 4337.68 | 43 |
| H14 | 2349.95 | -4161.62 | 3416.4 | 55 |
| H15 | 1654.39 | -5689.78 | 4454.17 | 58 |
| H16 | 2467.91 | -2303.95 | 5908.43 | 44 |

shI\_4082\_BeDa


# shI\_4082\_BeDa

Table 1 Crystal data and structure refinement for shI\_4082\_BeDa.

| Identification code | shI\_4082\_BeDa |
| Empirical formula | C32H24LaN7O13 |
| Formula weight | 853.49 |
| Temperature/K | 293(2) |
| Crystal system | monoclinic |
| Space group | P2/n |
| a/Å | 11.7434(7) |
| b/Å | 10.0978(5) |
| c/Å | 14.0667(9) |
| α/° | 90 |
| β/° | 108.771(7) |
| γ/° | 90 |
| Volume/Å3 | 1579.36(17) |
| Z | 2 |
| ρcalcg/cm3 | 1.795 |
| μ/mm‑1 | 1.436 |
| F(000) | 852.0 |
| Crystal size/mm3 | 0.25 × 0.05 × 0.02 |
| Radiation | Mo Kα (λ = 0.71073) |
| 2Θ range for data collection/° | 4.034 to 50.054 |
| Index ranges | -13 ≤ h ≤ 12, -12 ≤ k ≤ 11, -16 ≤ l ≤ 12 |
| Reflections collected | 5552 |
| Independent reflections | 2786 [Rint = 0.0333, Rsigma = 0.0569] |
| Data/restraints/parameters | 2786/0/241 |
| Goodness-of-fit on F2 | 0.976 |
| Final R indexes [I>=2σ (I)] | R1 = 0.0326, wR2 = 0.0460 |
| Final R indexes [all data] | R1 = 0.0433, wR2 = 0.0483 |
| Largest diff. peak/hole / e Å-3 | 0.51/-0.37 |
