# Supplementary material for: Novel Lanthanide (III) Complexes Derived from an Imidazole–Biphenyl–Carboxylate Ligand: Synthesis, Structure and Luminescence Properties
Source: Molecules. 2021 Nov 17;26(22):6942. doi: 10.3390/molecules26226942 (PMC8625298; doi:10.3390/molecules26226942)
Supplement: Supplementary file 1 [file molecules-26-06942-s001.zip › CRystallografic data/shI_4108_BeDa_tables.html]

shI\_4108\_BeDa


# shI\_4108\_BeDa

Table 1 Crystal data and structure refinement for shI\_4108\_BeDa.

| Identification code | shI\_4108\_BeDa |
| Empirical formula | C32H24DyN7O13 |
| Formula weight | 877.08 |
| Temperature/K | 180.05(10) |
| Crystal system | monoclinic |
| Space group | P2/n |
| a/Å | 11.5987(4) |
| b/Å | 10.0803(3) |
| c/Å | 13.9615(5) |
| α/° | 90 |
| β/° | 109.871(4) |
| γ/° | 90 |
| Volume/Å3 | 1535.17(10) |
| Z | 2 |
| ρcalcg/cm3 | 1.897 |
| μ/mm‑1 | 2.520 |
| F(000) | 870.0 |
| Crystal size/mm3 | 0.1 × 0.05 × 0.05 |
| Radiation | Mo Kα (λ = 0.71073) |
| 2Θ range for data collection/° | 3.962 to 50.054 |
| Index ranges | -13 ≤ h ≤ 13, -11 ≤ k ≤ 12, -16 ≤ l ≤ 16 |
| Reflections collected | 11123 |
| Independent reflections | 2713 [Rint = 0.0431, Rsigma = 0.0421] |
| Data/restraints/parameters | 2713/0/241 |
| Goodness-of-fit on F2 | 1.070 |
| Final R indexes [I>=2σ (I)] | R1 = 0.0301, wR2 = 0.0602 |
| Final R indexes [all data] | R1 = 0.0356, wR2 = 0.0622 |
| Largest diff. peak/hole / e Å-3 | 0.55/-0.60 |

Table 2 Fractional Atomic Coordinates (×104) and Equivalent Isotropic Displacement Parameters (Å2×103) for shI\_4108\_BeDa. Ueq is defined as 1/3 of of the trace of the orthogonalised UIJ tensor.

| Atom | *x* | *y* | *z* | U(eq) |
| --- | --- | --- | --- | --- |
| Dy1 | 2500 | 2436.9(2) | 7500 | 13.94(9) |
| O1 | 3742(2) | 4450(2) | 8152.8(19) | 19.2(6) |
| O2 | 3911(2) | 3463(2) | 6797.6(19) | 19.1(6) |
| O3 | 3354(2) | 2343(2) | 9393(2) | 20.0(6) |
| O4 | 4517(2) | 1597(3) | 8591.4(19) | 21.2(6) |
| O5 | 5134(2) | 1498(3) | 10255(2) | 28.5(7) |
| O6 | 2828(2) | 229(3) | 6860.0(19) | 19.8(6) |
| O7 | 2500 | -1649(4) | 7500 | 21.0(9) |
| N1 | 7446(3) | 12791(3) | 5453(2) | 15.4(7) |
| N2 | 8059(3) | 14166(3) | 4546(2) | 17.7(7) |
| N3 | 4364(3) | 1810(3) | 9438(2) | 18.6(7) |
| N4 | 2500 | -433(5) | 7500 | 18.4(10) |
| C1 | 4119(3) | 4444(4) | 7385(3) | 16.2(8) |
| C2 | 4752(3) | 5643(4) | 7175(3) | 13.9(8) |
| C3 | 4863(3) | 6787(4) | 7756(3) | 15.2(8) |
| C4 | 5331(3) | 7945(4) | 7496(3) | 15.3(8) |
| C5 | 5708(3) | 7990(4) | 6645(3) | 14.0(8) |
| C6 | 5613(3) | 6820(4) | 6075(3) | 17.5(8) |
| C7 | 5141(3) | 5674(4) | 6333(3) | 14.9(8) |
| C8 | 6161(3) | 9243(4) | 6340(3) | 15.2(8) |
| C9 | 5761(3) | 10475(4) | 6560(3) | 18.5(9) |
| C10 | 6180(3) | 11637(4) | 6279(3) | 18.5(9) |
| C11 | 7020(3) | 11585(4) | 5771(3) | 14.5(8) |
| C12 | 7437(3) | 10388(4) | 5544(3) | 16.7(8) |
| C13 | 7002(3) | 9231(4) | 5817(3) | 17.3(8) |
| C14 | 7703(4) | 13977(4) | 5980(3) | 21.6(9) |
| C15 | 8084(4) | 14828(4) | 5404(3) | 24.4(9) |
| C16 | 7663(3) | 12946(4) | 4575(3) | 17.5(9) |

Table 3 Anisotropic Displacement Parameters (Å2×103) for shI\_4108\_BeDa. The Anisotropic displacement factor exponent takes the form: -2π2[h2a\*2U11+2hka\*b\*U12+…].

| Atom | U11 | U22 | U33 | U23 | U13 | U12 |
| --- | --- | --- | --- | --- | --- | --- |
| Dy1 | 18.01(14) | 11.55(15) | 16.71(15) | 0 | 11.7(1) | 0 |
| O1 | 26.0(14) | 16.4(15) | 21.7(15) | -4.0(12) | 16.5(12) | -3.2(12) |
| O2 | 26.8(14) | 12.9(15) | 24.2(15) | -4.1(12) | 17.1(12) | -4.6(12) |
| O3 | 21.8(14) | 18.2(15) | 23.6(15) | 0.1(12) | 12.2(12) | 4.0(12) |
| O4 | 24.8(14) | 25.1(16) | 19.2(15) | -2.2(12) | 14.7(12) | 0.9(13) |
| O5 | 26.6(15) | 36.9(19) | 17.9(16) | -3.4(13) | 2.2(13) | 0.4(14) |
| O6 | 30.2(15) | 17.4(15) | 17.9(14) | 3.3(12) | 15.9(12) | -0.4(12) |
| O7 | 30(2) | 11(2) | 25(2) | 0 | 11.8(18) | 0 |
| N1 | 18.2(16) | 16.6(18) | 12.6(16) | 2.1(13) | 6.6(13) | -1.0(13) |
| N2 | 22.3(17) | 19.2(18) | 17.1(17) | 5.7(14) | 14.0(14) | -0.7(15) |
| N3 | 22.1(18) | 15.0(18) | 20.6(19) | -2.9(15) | 9.7(16) | -4.2(15) |
| N4 | 15(2) | 23(3) | 16(2) | 0 | 3(2) | 0 |
| C1 | 13.1(18) | 16(2) | 20(2) | 3.2(17) | 5.4(16) | 3.2(16) |
| C2 | 10.4(17) | 17(2) | 12.5(19) | 1.0(16) | 1.8(15) | 0.4(16) |
| C3 | 13.7(18) | 21(2) | 12.4(19) | 2.9(17) | 7.0(15) | -1.5(17) |
| C4 | 17.0(19) | 11.1(19) | 18(2) | -3.8(16) | 6.3(16) | -1.0(16) |
| C5 | 10.4(18) | 17(2) | 15.2(19) | 2.8(16) | 4.7(15) | -0.5(15) |
| C6 | 17.7(19) | 23(2) | 15(2) | -0.5(17) | 9.6(16) | -0.4(18) |
| C7 | 17.5(19) | 13(2) | 18(2) | -1.6(16) | 10.8(16) | -0.8(16) |
| C8 | 12.3(18) | 19(2) | 12.7(19) | 1.7(16) | 2.8(15) | -2.1(16) |
| C9 | 22(2) | 19(2) | 21(2) | 3.2(17) | 15.7(17) | 1.5(17) |
| C10 | 22(2) | 19(2) | 17(2) | -1.0(17) | 10.4(17) | 1.8(17) |
| C11 | 16.9(19) | 15(2) | 12.6(19) | 1.2(16) | 6.7(16) | -4.0(16) |
| C12 | 17.7(19) | 22(2) | 15.0(19) | -1.1(17) | 11.0(16) | -2.4(17) |
| C13 | 18.6(19) | 18(2) | 17(2) | -2.0(17) | 8.5(16) | 3.1(17) |
| C14 | 33(2) | 22(2) | 13(2) | -5.7(17) | 11.3(18) | -8.5(19) |
| C15 | 32(2) | 22(2) | 21(2) | -1.3(18) | 10.7(19) | -4.7(19) |
| C16 | 17.5(19) | 21(2) | 17(2) | 2.1(17) | 8.7(17) | 2.6(17) |

Table 4 Bond Lengths for shI\_4108\_BeDa.

| Atom | Atom | Length/Å |  | Atom | Atom | Length/Å |
| --- | --- | --- | --- | --- | --- | --- |
| Dy1 | O11 | 2.476(2) |  | N1 | C14 | 1.381(5) |
| Dy1 | O1 | 2.476(2) |  | N1 | C16 | 1.342(5) |
| Dy1 | O21 | 2.409(2) |  | N2 | C15 | 1.362(5) |
| Dy1 | O2 | 2.409(2) |  | N2 | C16 | 1.318(5) |
| Dy1 | O3 | 2.490(3) |  | C1 | C2 | 1.494(5) |
| Dy1 | O31 | 2.490(3) |  | C2 | C3 | 1.391(5) |
| Dy1 | O4 | 2.468(3) |  | C2 | C7 | 1.396(5) |
| Dy1 | O41 | 2.468(3) |  | C3 | C4 | 1.387(5) |
| Dy1 | O6 | 2.476(3) |  | C4 | C5 | 1.399(5) |
| Dy1 | O61 | 2.476(3) |  | C5 | C6 | 1.406(5) |
| Dy1 | C11 | 2.802(4) |  | C5 | C8 | 1.485(5) |
| Dy1 | C1 | 2.802(4) |  | C6 | C7 | 1.378(5) |
| O1 | C1 | 1.288(4) |  | C8 | C9 | 1.397(5) |
| O2 | C1 | 1.255(4) |  | C8 | C13 | 1.402(5) |
| O3 | N3 | 1.271(4) |  | C9 | C10 | 1.376(5) |
| O4 | N3 | 1.271(4) |  | C10 | C11 | 1.387(5) |
| O5 | N3 | 1.227(4) |  | C11 | C12 | 1.376(5) |
| O6 | N4 | 1.273(3) |  | C12 | C13 | 1.376(5) |
| O7 | N4 | 1.225(5) |  | C14 | C15 | 1.348(5) |
| N1 | C11 | 1.438(5) |  |  |  |  |

11/2-X,+Y,3/2-Z

Table 5 Bond Angles for shI\_4108\_BeDa.

| Atom | Atom | Atom | Angle/˚ |  | Atom | Atom | Atom | Angle/˚ |
| --- | --- | --- | --- | --- | --- | --- | --- | --- |
| O11 | Dy1 | O1 | 69.95(12) |  | O6 | Dy1 | O3 | 107.12(8) |
| O11 | Dy1 | O31 | 70.65(8) |  | O61 | Dy1 | O3 | 68.71(8) |
| O1 | Dy1 | O31 | 113.20(8) |  | O6 | Dy1 | O61 | 51.93(12) |
| O1 | Dy1 | O3 | 70.65(8) |  | O6 | Dy1 | C11 | 149.11(9) |
| O11 | Dy1 | O3 | 113.20(8) |  | O61 | Dy1 | C1 | 149.11(9) |
| O1 | Dy1 | C11 | 72.84(9) |  | O61 | Dy1 | C11 | 116.11(10) |
| O1 | Dy1 | C1 | 27.36(10) |  | O6 | Dy1 | C1 | 116.11(10) |
| O11 | Dy1 | C1 | 72.84(9) |  | C11 | Dy1 | C1 | 87.56(15) |
| O11 | Dy1 | C11 | 27.36(10) |  | C1 | O1 | Dy1 | 90.6(2) |
| O21 | Dy1 | O11 | 53.63(8) |  | C1 | O2 | Dy1 | 94.5(2) |
| O21 | Dy1 | O1 | 83.64(8) |  | N3 | O3 | Dy1 | 95.5(2) |
| O2 | Dy1 | O11 | 83.64(8) |  | N3 | O4 | Dy1 | 96.5(2) |
| O2 | Dy1 | O1 | 53.63(8) |  | N4 | O6 | Dy1 | 95.7(2) |
| O21 | Dy1 | O2 | 129.15(12) |  | C14 | N1 | C11 | 127.1(3) |
| O21 | Dy1 | O3 | 70.39(8) |  | C16 | N1 | C11 | 124.8(3) |
| O21 | Dy1 | O31 | 111.62(8) |  | C16 | N1 | C14 | 108.1(3) |
| O2 | Dy1 | O3 | 111.62(8) |  | C16 | N2 | C15 | 109.5(3) |
| O2 | Dy1 | O31 | 70.39(8) |  | O3 | N3 | Dy1 | 58.67(17) |
| O21 | Dy1 | O4 | 121.93(8) |  | O3 | N3 | O4 | 116.3(3) |
| O2 | Dy1 | O41 | 121.93(8) |  | O4 | N3 | Dy1 | 57.68(17) |
| O2 | Dy1 | O4 | 76.46(9) |  | O5 | N3 | Dy1 | 177.6(3) |
| O21 | Dy1 | O41 | 76.46(9) |  | O5 | N3 | O3 | 121.5(3) |
| O2 | Dy1 | O61 | 137.92(8) |  | O5 | N3 | O4 | 122.2(3) |
| O21 | Dy1 | O6 | 137.92(8) |  | O6 | N4 | Dy1 | 58.4(2) |
| O21 | Dy1 | O61 | 91.71(8) |  | O61 | N4 | Dy1 | 58.4(2) |
| O2 | Dy1 | O6 | 91.71(8) |  | O6 | N4 | O61 | 116.8(4) |
| O2 | Dy1 | C1 | 26.52(10) |  | O7 | N4 | Dy1 | 180.0 |
| O2 | Dy1 | C11 | 105.95(10) |  | O7 | N4 | O61 | 121.6(2) |
| O21 | Dy1 | C1 | 105.95(10) |  | O7 | N4 | O6 | 121.6(2) |
| O21 | Dy1 | C11 | 26.52(10) |  | O1 | C1 | Dy1 | 62.09(19) |
| O31 | Dy1 | O3 | 175.63(12) |  | O1 | C1 | C2 | 119.2(3) |
| O31 | Dy1 | C11 | 93.19(10) |  | O2 | C1 | Dy1 | 58.99(19) |
| O3 | Dy1 | C1 | 93.19(10) |  | O2 | C1 | O1 | 120.2(3) |
| O31 | Dy1 | C1 | 89.97(10) |  | O2 | C1 | C2 | 120.5(3) |
| O3 | Dy1 | C11 | 89.97(10) |  | C2 | C1 | Dy1 | 168.2(2) |
| O4 | Dy1 | O1 | 75.10(8) |  | C3 | C2 | C1 | 120.7(3) |
| O4 | Dy1 | O11 | 145.03(9) |  | C3 | C2 | C7 | 118.7(3) |
| O41 | Dy1 | O1 | 145.03(9) |  | C7 | C2 | C1 | 120.3(3) |
| O41 | Dy1 | O11 | 75.10(8) |  | C4 | C3 | C2 | 120.8(3) |
| O41 | Dy1 | O31 | 51.66(8) |  | C3 | C4 | C5 | 120.8(4) |
| O4 | Dy1 | O31 | 126.45(8) |  | C4 | C5 | C6 | 117.8(3) |
| O41 | Dy1 | O3 | 126.45(8) |  | C4 | C5 | C8 | 121.0(3) |
| O4 | Dy1 | O3 | 51.66(8) |  | C6 | C5 | C8 | 121.2(3) |
| O4 | Dy1 | O41 | 139.86(13) |  | C7 | C6 | C5 | 121.1(4) |
| O4 | Dy1 | O6 | 71.61(8) |  | C6 | C7 | C2 | 120.7(4) |
| O41 | Dy1 | O61 | 71.61(8) |  | C9 | C8 | C5 | 121.1(3) |
| O41 | Dy1 | O6 | 72.45(9) |  | C9 | C8 | C13 | 117.7(3) |
| O4 | Dy1 | O61 | 72.45(9) |  | C13 | C8 | C5 | 121.3(3) |
| O4 | Dy1 | C11 | 136.53(10) |  | C10 | C9 | C8 | 121.2(3) |
| O41 | Dy1 | C1 | 136.53(10) |  | C9 | C10 | C11 | 119.5(4) |
| O41 | Dy1 | C11 | 76.69(9) |  | C10 | C11 | N1 | 120.0(3) |
| O4 | Dy1 | C1 | 76.69(10) |  | C12 | C11 | N1 | 119.1(3) |
| O61 | Dy1 | O11 | 136.71(8) |  | C12 | C11 | C10 | 120.9(3) |
| O6 | Dy1 | O11 | 138.20(8) |  | C13 | C12 | C11 | 119.3(3) |
| O61 | Dy1 | O1 | 138.20(8) |  | C12 | C13 | C8 | 121.5(4) |
| O6 | Dy1 | O1 | 136.71(8) |  | C15 | C14 | N1 | 106.8(3) |
| O61 | Dy1 | O31 | 107.12(8) |  | C14 | C15 | N2 | 107.4(4) |
| O6 | Dy1 | O31 | 68.71(8) |  | N2 | C16 | N1 | 108.2(3) |

11/2-X,+Y,3/2-Z

Table 6 Hydrogen Bonds for shI\_4108\_BeDa.

| D | H | A | d(D-H)/Å | d(H-A)/Å | d(D-A)/Å | D-H-A/° |
| --- | --- | --- | --- | --- | --- | --- |
| N2 | H2 | O11 | 0.86 | 1.88 | 2.722(4) | 167.7 |
| C15 | H15 | O31 | 0.93 | 2.57 | 3.244(5) | 130.1 |
| C16 | H16 | O72 | 0.93 | 2.28 | 3.126(4) | 151.8 |

11/2+X,2-Y,-1/2+Z; 21-X,1-Y,1-Z

Table 7 Torsion Angles for shI\_4108\_BeDa.

| A | B | C | D | Angle/˚ |  | A | B | C | D | Angle/˚ |
| --- | --- | --- | --- | --- | --- | --- | --- | --- | --- | --- |
| Dy1 | O1 | C1 | O2 | -10.4(3) |  | C4 | C5 | C8 | C13 | 152.1(3) |
| Dy1 | O1 | C1 | C2 | 166.6(3) |  | C5 | C6 | C7 | C2 | -0.5(5) |
| Dy1 | O2 | C1 | O1 | 10.7(3) |  | C5 | C8 | C9 | C10 | 179.9(3) |
| Dy1 | O2 | C1 | C2 | -166.3(3) |  | C5 | C8 | C13 | C12 | -179.1(3) |
| Dy1 | O3 | N3 | O4 | 1.5(3) |  | C6 | C5 | C8 | C9 | 150.2(4) |
| Dy1 | O3 | N3 | O5 | -177.2(3) |  | C6 | C5 | C8 | C13 | -29.6(5) |
| Dy1 | O4 | N3 | O3 | -1.5(3) |  | C7 | C2 | C3 | C4 | 1.1(5) |
| Dy1 | O4 | N3 | O5 | 177.2(3) |  | C8 | C5 | C6 | C7 | -177.0(3) |
| Dy1 | O6 | N4 | O61 | -0.002(2) |  | C8 | C9 | C10 | C11 | -0.3(6) |
| Dy1 | O6 | N4 | O7 | 180.000(1) |  | C9 | C8 | C13 | C12 | 1.1(5) |
| Dy1 | C1 | C2 | C3 | 88.8(14) |  | C9 | C10 | C11 | N1 | 178.7(3) |
| Dy1 | C1 | C2 | C7 | -85.3(14) |  | C9 | C10 | C11 | C12 | 0.1(5) |
| O1 | C1 | C2 | C3 | -4.3(5) |  | C10 | C11 | C12 | C13 | 0.7(5) |
| O1 | C1 | C2 | C7 | -178.3(3) |  | C11 | N1 | C14 | C15 | 179.4(3) |
| O2 | C1 | C2 | C3 | 172.8(3) |  | C11 | N1 | C16 | N2 | -179.0(3) |
| O2 | C1 | C2 | C7 | -1.3(5) |  | C11 | C12 | C13 | C8 | -1.3(5) |
| N1 | C11 | C12 | C13 | -177.9(3) |  | C13 | C8 | C9 | C10 | -0.3(5) |
| N1 | C14 | C15 | N2 | -0.1(4) |  | C14 | N1 | C11 | C10 | 38.2(5) |
| C1 | C2 | C3 | C4 | -173.0(3) |  | C14 | N1 | C11 | C12 | -143.2(4) |
| C1 | C2 | C7 | C6 | 173.5(3) |  | C14 | N1 | C16 | N2 | 0.9(4) |
| C2 | C3 | C4 | C5 | -0.3(5) |  | C15 | N2 | C16 | N1 | -1.0(4) |
| C3 | C2 | C7 | C6 | -0.7(5) |  | C16 | N1 | C11 | C10 | -141.9(4) |
| C3 | C4 | C5 | C6 | -0.9(5) |  | C16 | N1 | C11 | C12 | 36.7(5) |
| C3 | C4 | C5 | C8 | 177.5(3) |  | C16 | N1 | C14 | C15 | -0.5(4) |
| C4 | C5 | C6 | C7 | 1.3(5) |  | C16 | N2 | C15 | C14 | 0.7(4) |
| C4 | C5 | C8 | C9 | -28.2(5) |  |  |  |  |  |  |

11/2-X,+Y,3/2-Z

Table 8 Hydrogen Atom Coordinates (Å×104) and Isotropic Displacement Parameters (Å2×103) for shI\_4108\_BeDa.

| Atom | *x* | *y* | *z* | U(eq) |
| --- | --- | --- | --- | --- |
| H2 | 8268.83 | 14493.78 | 4060.99 | 21 |
| H3 | 4620.77 | 6775.71 | 8326.59 | 18 |
| H4 | 5394.64 | 8701.95 | 7892.06 | 18 |
| H6 | 5874 | 6820.28 | 5514.88 | 21 |
| H7 | 5080.08 | 4913.9 | 5941.31 | 18 |
| H9 | 5200.21 | 10511.11 | 6903.21 | 22 |
| H10 | 5903 | 12450.65 | 6427.9 | 22 |
| H12 | 8006.77 | 10361.79 | 5209.45 | 20 |
| H13 | 7270.87 | 8421.87 | 5651.39 | 21 |
| H14 | 7628.65 | 14153.75 | 6609.72 | 26 |
| H15 | 8319.98 | 15705.07 | 5563.1 | 29 |
| H16 | 7553.11 | 12304.02 | 4074.05 | 21 |

shI\_4108\_BeDa


# shI\_4108\_BeDa

Table 1 Crystal data and structure refinement for shI\_4108\_BeDa.

| Identification code | shI\_4108\_BeDa |
| Empirical formula | C32H24DyN7O13 |
| Formula weight | 877.08 |
| Temperature/K | 180.05(10) |
| Crystal system | monoclinic |
| Space group | P2/n |
| a/Å | 11.5987(4) |
| b/Å | 10.0803(3) |
| c/Å | 13.9615(5) |
| α/° | 90 |
| β/° | 109.871(4) |
| γ/° | 90 |
| Volume/Å3 | 1535.17(10) |
| Z | 2 |
| ρcalcg/cm3 | 1.897 |
| μ/mm‑1 | 2.520 |
| F(000) | 870.0 |
| Crystal size/mm3 | 0.1 × 0.05 × 0.05 |
| Radiation | Mo Kα (λ = 0.71073) |
| 2Θ range for data collection/° | 3.962 to 50.054 |
| Index ranges | -13 ≤ h ≤ 13, -11 ≤ k ≤ 12, -16 ≤ l ≤ 16 |
| Reflections collected | 11123 |
| Independent reflections | 2713 [Rint = 0.0431, Rsigma = 0.0421] |
| Data/restraints/parameters | 2713/0/241 |
| Goodness-of-fit on F2 | 1.070 |
| Final R indexes [I>=2σ (I)] | R1 = 0.0301, wR2 = 0.0602 |
| Final R indexes [all data] | R1 = 0.0356, wR2 = 0.0622 |
| Largest diff. peak/hole / e Å-3 | 0.55/-0.60 |
