# Supplementary material for: Novel Lanthanide (III) Complexes Derived from an Imidazole–Biphenyl–Carboxylate Ligand: Synthesis, Structure and Luminescence Properties
Source: Molecules. 2021 Nov 17;26(22):6942. doi: 10.3390/molecules26226942 (PMC8625298; doi:10.3390/molecules26226942)
Supplement: Supplementary file 1 [file molecules-26-06942-s001.zip › CRystallografic data/shI_4151_BeDa_tables.html]

shI\_4151\_BeDa


# shI\_4151\_BeDa

Table 1 Crystal data and structure refinement for shI\_4151\_BeDa.

| Identification code | shI\_4151\_BeDa |
| Empirical formula | C32H24HoN7O13 |
| Formula weight | 879.51 |
| Temperature/K | 180.00(14) |
| Crystal system | monoclinic |
| Space group | P2/n |
| a/Å | 11.6138(3) |
| b/Å | 10.0845(2) |
| c/Å | 14.0087(4) |
| α/° | 90 |
| β/° | 109.726(3) |
| γ/° | 90 |
| Volume/Å3 | 1544.41(7) |
| Z | 2 |
| ρcalcg/cm3 | 1.891 |
| μ/mm‑1 | 2.647 |
| F(000) | 872.0 |
| Crystal size/mm3 | 0.25 × 0.05 × 0.05 |
| Radiation | Mo Kα (λ = 0.71073) |
| 2Θ range for data collection/° | 3.956 to 50.044 |
| Index ranges | -13 ≤ h ≤ 13, -12 ≤ k ≤ 12, -16 ≤ l ≤ 16 |
| Reflections collected | 22162 |
| Independent reflections | 2727 [Rint = 0.0557, Rsigma = 0.0343] |
| Data/restraints/parameters | 2727/0/241 |
| Goodness-of-fit on F2 | 1.040 |
| Final R indexes [I>=2σ (I)] | R1 = 0.0304, wR2 = 0.0737 |
| Final R indexes [all data] | R1 = 0.0359, wR2 = 0.0764 |
| Largest diff. peak/hole / e Å-3 | 0.86/-0.66 |

Table 2 Fractional Atomic Coordinates (×104) and Equivalent Isotropic Displacement Parameters (Å2×103) for shI\_4151\_BeDa. Ueq is defined as 1/3 of of the trace of the orthogonalised UIJ tensor.

| Atom | *x* | *y* | *z* | U(eq) |
| --- | --- | --- | --- | --- |
| Ho1 | 7500 | 7573.5(3) | 2500 | 18.49(11) |
| O1 | 6244(3) | 5534(3) | 1859(2) | 18.7(7) |
| O2 | 6075(3) | 6518(3) | 3204(2) | 16.8(6) |
| O3 | 8371(3) | 7675(3) | 4405(2) | 19.0(6) |
| O4 | 9545(3) | 8416(3) | 3615(2) | 20.9(7) |
| O5 | 10148(3) | 8512(3) | 5267(2) | 28.8(8) |
| O6 | 7173(3) | 9824(3) | 3138(2) | 18.1(6) |
| O7 | 7500 | 11695(4) | 2500 | 22.5(10) |
| N1 | 2554(3) | -2788(3) | 4553(3) | 15.3(7) |
| N2 | 1939(3) | -4154(4) | 5458(3) | 19.7(8) |
| N3 | 9378(3) | 8209(3) | 4454(3) | 17.4(8) |
| N4 | 7500 | 10480(5) | 2500 | 16.4(11) |
| C1 | 5868(4) | 5528(4) | 2612(3) | 16.2(9) |
| C2 | 5241(4) | 4338(4) | 2828(3) | 14.0(8) |
| C3 | 5120(4) | 3196(4) | 2251(3) | 15.4(9) |
| C4 | 4662(4) | 2048(4) | 2509(3) | 15.4(9) |
| C5 | 4296(4) | 1997(4) | 3364(3) | 14.5(9) |
| C6 | 4392(4) | 3163(4) | 3932(3) | 15.6(9) |
| C7 | 4860(4) | 4303(4) | 3675(3) | 17.4(9) |
| C8 | 3835(4) | 747(4) | 3661(3) | 14.6(9) |
| C9 | 4236(4) | -487(4) | 3445(3) | 19.3(9) |
| C10 | 3821(4) | -1654(4) | 3728(3) | 17.1(9) |
| C11 | 2979(4) | -1590(4) | 4238(3) | 15.2(9) |
| C12 | 2562(4) | -383(4) | 4458(3) | 18.3(9) |
| C13 | 2995(4) | 766(4) | 4179(3) | 17.2(9) |
| C14 | 2283(4) | -3980(4) | 4031(4) | 24.6(10) |
| C15 | 1903(4) | -4821(4) | 4606(3) | 24.2(10) |
| C16 | 2337(4) | -2941(4) | 5428(3) | 17.9(9) |

Table 3 Anisotropic Displacement Parameters (Å2×103) for shI\_4151\_BeDa. The Anisotropic displacement factor exponent takes the form: -2π2[h2a\*2U11+2hka\*b\*U12+…].

| Atom | U11 | U22 | U33 | U23 | U13 | U12 |
| --- | --- | --- | --- | --- | --- | --- |
| Ho1 | 22.15(17) | 13.97(16) | 24.20(18) | 0 | 14.18(13) | 0 |
| O1 | 26.4(16) | 13.2(15) | 24.2(16) | -3.7(12) | 18.8(14) | -3.7(12) |
| O2 | 23.9(16) | 9.7(14) | 24.0(16) | -3.4(12) | 17.3(13) | -4.8(12) |
| O3 | 21.2(16) | 16.4(15) | 23.4(16) | 0.8(13) | 12.8(13) | 0.3(12) |
| O4 | 23.7(16) | 21.7(16) | 22.3(17) | 0.4(13) | 14.2(14) | -2.0(13) |
| O5 | 25.7(18) | 34.3(19) | 23.7(18) | 4.4(15) | 4.9(15) | 0.1(15) |
| O6 | 27.1(16) | 12.8(14) | 19.7(15) | 2.5(13) | 14.9(13) | 1.1(12) |
| O7 | 32(2) | 10(2) | 28(2) | 0 | 13(2) | 0 |
| N1 | 16.8(17) | 14.0(17) | 17.7(18) | 1.1(14) | 9.2(15) | -0.5(14) |
| N2 | 18.6(18) | 19.9(19) | 24(2) | 10.5(16) | 11.9(16) | 1.2(15) |
| N3 | 19.2(19) | 10.1(17) | 25(2) | 3.8(16) | 10.7(17) | 3.5(15) |
| N4 | 16(2) | 10(2) | 22(3) | 0 | 5(2) | 0 |
| C1 | 14(2) | 14(2) | 22(2) | 1.2(18) | 8.8(18) | 3.2(16) |
| C2 | 15(2) | 11(2) | 17(2) | 0.1(17) | 6.8(17) | -0.6(16) |
| C3 | 17(2) | 17(2) | 14(2) | 0.7(17) | 8.3(17) | 0.6(17) |
| C4 | 16(2) | 10.7(19) | 20(2) | -3.0(17) | 7.0(18) | -2.2(16) |
| C5 | 12(2) | 11(2) | 20(2) | 0.9(17) | 5.4(17) | -1.1(16) |
| C6 | 18(2) | 15(2) | 17(2) | 0.9(17) | 11.5(18) | -1.4(17) |
| C7 | 20(2) | 15(2) | 21(2) | -2.7(18) | 12.6(19) | 0.4(17) |
| C8 | 14(2) | 13(2) | 16(2) | 1.2(17) | 3.7(17) | -0.7(16) |
| C9 | 19(2) | 19(2) | 26(2) | 2.0(19) | 15.8(19) | 2.8(17) |
| C10 | 19(2) | 12(2) | 23(2) | -1.7(18) | 11.2(19) | -1.6(17) |
| C11 | 19(2) | 12(2) | 14(2) | 2.1(17) | 4.7(17) | -4.1(17) |
| C12 | 20(2) | 19(2) | 23(2) | 1.2(18) | 15.5(19) | 0.7(17) |
| C13 | 20(2) | 14(2) | 21(2) | -2.4(18) | 10.1(18) | -0.1(17) |
| C14 | 35(3) | 16(2) | 25(2) | -6.5(19) | 14(2) | -8.8(19) |
| C15 | 29(2) | 16(2) | 27(3) | -1(2) | 10(2) | -7.1(19) |
| C16 | 19(2) | 17(2) | 19(2) | 3.2(18) | 8.7(18) | 3.8(17) |

Table 4 Bond Lengths for shI\_4151\_BeDa.

| Atom | Atom | Length/Å |  | Atom | Atom | Length/Å |
| --- | --- | --- | --- | --- | --- | --- |
| Ho1 | O11 | 2.506(3) |  | N1 | C14 | 1.387(5) |
| Ho1 | O1 | 2.506(3) |  | N1 | C16 | 1.340(5) |
| Ho1 | O21 | 2.438(3) |  | N2 | C15 | 1.359(6) |
| Ho1 | O2 | 2.438(3) |  | N2 | C16 | 1.314(5) |
| Ho1 | O3 | 2.516(3) |  | C1 | C2 | 1.487(6) |
| Ho1 | O31 | 2.516(3) |  | C2 | C3 | 1.387(6) |
| Ho1 | O41 | 2.509(3) |  | C2 | C7 | 1.400(6) |
| Ho1 | O4 | 2.509(3) |  | C3 | C4 | 1.373(6) |
| Ho1 | O61 | 2.514(3) |  | C4 | C5 | 1.400(6) |
| Ho1 | O6 | 2.514(3) |  | C5 | C6 | 1.403(6) |
| Ho1 | C1 | 2.841(4) |  | C5 | C8 | 1.483(6) |
| Ho1 | C11 | 2.841(4) |  | C6 | C7 | 1.371(6) |
| O1 | C1 | 1.271(5) |  | C8 | C9 | 1.397(6) |
| O2 | C1 | 1.268(5) |  | C8 | C13 | 1.399(6) |
| O3 | N3 | 1.267(4) |  | C9 | C10 | 1.379(6) |
| O4 | N3 | 1.271(4) |  | C10 | C11 | 1.394(6) |
| O5 | N3 | 1.226(5) |  | C11 | C12 | 1.383(6) |
| O6 | N4 | 1.268(4) |  | C12 | C13 | 1.371(6) |
| O7 | N4 | 1.226(6) |  | C14 | C15 | 1.344(6) |
| N1 | C11 | 1.429(5) |  |  |  |  |

13/2-X,+Y,1/2-Z

Table 5 Bond Angles for shI\_4151\_BeDa.

| Atom | Atom | Atom | Angle/˚ |  | Atom | Atom | Atom | Angle/˚ |
| --- | --- | --- | --- | --- | --- | --- | --- | --- |
| O11 | Ho1 | O1 | 69.68(13) |  | O6 | Ho1 | O31 | 106.56(9) |
| O1 | Ho1 | O3 | 113.00(9) |  | O61 | Ho1 | O3 | 106.56(9) |
| O1 | Ho1 | O31 | 71.12(9) |  | O61 | Ho1 | O6 | 50.99(13) |
| O11 | Ho1 | O3 | 71.12(9) |  | O61 | Ho1 | C11 | 116.88(10) |
| O11 | Ho1 | O31 | 113.00(9) |  | O6 | Ho1 | C11 | 149.13(11) |
| O11 | Ho1 | O4 | 74.98(10) |  | O6 | Ho1 | C1 | 116.88(10) |
| O1 | Ho1 | O41 | 74.98(10) |  | O61 | Ho1 | C1 | 149.13(11) |
| O1 | Ho1 | O4 | 144.59(10) |  | C1 | Ho1 | C11 | 86.91(16) |
| O11 | Ho1 | O41 | 144.59(10) |  | C1 | O1 | Ho1 | 91.6(2) |
| O1 | Ho1 | O6 | 136.79(9) |  | C1 | O2 | Ho1 | 94.8(2) |
| O11 | Ho1 | O6 | 138.84(9) |  | N3 | O3 | Ho1 | 96.2(2) |
| O1 | Ho1 | O61 | 138.84(9) |  | N3 | O4 | Ho1 | 96.4(2) |
| O11 | Ho1 | O61 | 136.79(9) |  | N4 | O6 | Ho1 | 95.9(2) |
| O1 | Ho1 | C1 | 26.56(11) |  | C14 | N1 | C11 | 127.6(4) |
| O1 | Ho1 | C11 | 72.72(10) |  | C16 | N1 | C11 | 124.8(4) |
| O11 | Ho1 | C1 | 72.71(10) |  | C16 | N1 | C14 | 107.7(4) |
| O11 | Ho1 | C11 | 26.56(11) |  | C16 | N2 | C15 | 109.8(4) |
| O21 | Ho1 | O1 | 83.64(9) |  | O3 | N3 | Ho1 | 58.39(19) |
| O2 | Ho1 | O11 | 83.64(9) |  | O3 | N3 | O4 | 116.4(3) |
| O2 | Ho1 | O1 | 52.72(9) |  | O4 | N3 | Ho1 | 58.09(19) |
| O21 | Ho1 | O11 | 52.73(9) |  | O5 | N3 | Ho1 | 178.1(3) |
| O2 | Ho1 | O21 | 128.24(13) |  | O5 | N3 | O3 | 121.8(4) |
| O2 | Ho1 | O31 | 111.21(9) |  | O5 | N3 | O4 | 121.8(3) |
| O2 | Ho1 | O3 | 70.97(9) |  | O61 | N4 | Ho1 | 58.6(2) |
| O21 | Ho1 | O31 | 70.97(9) |  | O6 | N4 | Ho1 | 58.6(2) |
| O21 | Ho1 | O3 | 111.21(9) |  | O6 | N4 | O61 | 117.1(4) |
| O2 | Ho1 | O41 | 76.74(10) |  | O7 | N4 | Ho1 | 180.0 |
| O2 | Ho1 | O4 | 121.66(9) |  | O7 | N4 | O61 | 121.4(2) |
| O21 | Ho1 | O4 | 76.74(10) |  | O7 | N4 | O6 | 121.4(2) |
| O21 | Ho1 | O41 | 121.66(9) |  | O1 | C1 | Ho1 | 61.8(2) |
| O21 | Ho1 | O6 | 137.91(9) |  | O1 | C1 | C2 | 120.1(4) |
| O2 | Ho1 | O6 | 92.63(9) |  | O2 | C1 | Ho1 | 58.8(2) |
| O21 | Ho1 | O61 | 92.63(9) |  | O2 | C1 | O1 | 119.8(4) |
| O2 | Ho1 | O61 | 137.91(9) |  | O2 | C1 | C2 | 120.0(4) |
| O21 | Ho1 | C1 | 105.19(10) |  | C2 | C1 | Ho1 | 168.2(3) |
| O2 | Ho1 | C1 | 26.41(10) |  | C3 | C2 | C1 | 120.8(4) |
| O2 | Ho1 | C11 | 105.19(10) |  | C3 | C2 | C7 | 118.3(4) |
| O21 | Ho1 | C11 | 26.41(10) |  | C7 | C2 | C1 | 120.6(4) |
| O3 | Ho1 | O31 | 175.32(13) |  | C4 | C3 | C2 | 121.3(4) |
| O3 | Ho1 | C11 | 92.93(11) |  | C3 | C4 | C5 | 120.9(4) |
| O31 | Ho1 | C11 | 90.47(11) |  | C4 | C5 | C6 | 117.7(4) |
| O31 | Ho1 | C1 | 92.93(11) |  | C4 | C5 | C8 | 120.9(4) |
| O3 | Ho1 | C1 | 90.47(11) |  | C6 | C5 | C8 | 121.3(4) |
| O4 | Ho1 | O3 | 50.85(9) |  | C7 | C6 | C5 | 121.1(4) |
| O4 | Ho1 | O31 | 127.13(9) |  | C6 | C7 | C2 | 120.7(4) |
| O41 | Ho1 | O31 | 50.85(9) |  | C9 | C8 | C5 | 121.2(4) |
| O41 | Ho1 | O3 | 127.13(9) |  | C9 | C8 | C13 | 117.7(4) |
| O41 | Ho1 | O4 | 140.41(14) |  | C13 | C8 | C5 | 121.1(4) |
| O41 | Ho1 | O61 | 72.40(10) |  | C10 | C9 | C8 | 121.6(4) |
| O4 | Ho1 | O61 | 72.00(9) |  | C9 | C10 | C11 | 118.8(4) |
| O41 | Ho1 | O6 | 72.00(9) |  | C10 | C11 | N1 | 119.7(4) |
| O4 | Ho1 | O6 | 72.41(10) |  | C12 | C11 | N1 | 119.4(4) |
| O41 | Ho1 | C11 | 136.08(11) |  | C12 | C11 | C10 | 120.9(4) |
| O4 | Ho1 | C1 | 136.08(11) |  | C13 | C12 | C11 | 119.4(4) |
| O4 | Ho1 | C11 | 76.78(11) |  | C12 | C13 | C8 | 121.6(4) |
| O41 | Ho1 | C1 | 76.78(11) |  | C15 | C14 | N1 | 107.0(4) |
| O61 | Ho1 | O31 | 68.97(9) |  | C14 | C15 | N2 | 107.2(4) |
| O6 | Ho1 | O3 | 68.97(9) |  | N2 | C16 | N1 | 108.3(4) |

13/2-X,+Y,1/2-Z

Table 6 Hydrogen Bonds for shI\_4151\_BeDa.

| D | H | A | d(D-H)/Å | d(H-A)/Å | d(D-A)/Å | D-H-A/° |
| --- | --- | --- | --- | --- | --- | --- |
| N2 | H2 | O11 | 0.86 | 1.89 | 2.739(4) | 167.7 |
| C15 | H15 | O32 | 0.93 | 2.57 | 3.256(5) | 131.1 |
| C16 | H16 | O73 | 0.93 | 2.27 | 3.110(5) | 150.3 |

1-1/2+X,-Y,1/2+Z; 21-X,-Y,1-Z; 31-X,1-Y,1-Z

Table 7 Torsion Angles for shI\_4151\_BeDa.

| A | B | C | D | Angle/˚ |  | A | B | C | D | Angle/˚ |
| --- | --- | --- | --- | --- | --- | --- | --- | --- | --- | --- |
| Ho1 | O1 | C1 | O2 | 10.3(4) |  | C4 | C5 | C8 | C13 | -151.3(4) |
| Ho1 | O1 | C1 | C2 | -166.6(3) |  | C5 | C6 | C7 | C2 | 0.8(6) |
| Ho1 | O2 | C1 | O1 | -10.6(4) |  | C5 | C8 | C9 | C10 | 179.3(4) |
| Ho1 | O2 | C1 | C2 | 166.3(3) |  | C5 | C8 | C13 | C12 | -180.0(4) |
| Ho1 | O3 | N3 | O4 | -2.0(3) |  | C6 | C5 | C8 | C9 | -149.7(4) |
| Ho1 | O3 | N3 | O5 | 177.7(3) |  | C6 | C5 | C8 | C13 | 29.3(6) |
| Ho1 | O4 | N3 | O3 | 2.1(3) |  | C7 | C2 | C3 | C4 | -1.3(6) |
| Ho1 | O4 | N3 | O5 | -177.7(3) |  | C8 | C5 | C6 | C7 | 177.7(4) |
| Ho1 | O6 | N4 | O61 | 0.002(1) |  | C8 | C9 | C10 | C11 | 0.2(6) |
| Ho1 | O6 | N4 | O7 | 180.000(1) |  | C9 | C8 | C13 | C12 | -0.9(6) |
| Ho1 | C1 | C2 | C3 | -92.7(14) |  | C9 | C10 | C11 | N1 | -178.9(4) |
| Ho1 | C1 | C2 | C7 | 81.5(15) |  | C9 | C10 | C11 | C12 | 0.1(6) |
| O1 | C1 | C2 | C3 | 3.7(6) |  | C10 | C11 | C12 | C13 | -0.7(6) |
| O1 | C1 | C2 | C7 | 177.8(4) |  | C11 | N1 | C14 | C15 | -179.3(4) |
| O2 | C1 | C2 | C3 | -173.2(4) |  | C11 | N1 | C16 | N2 | 179.0(4) |
| O2 | C1 | C2 | C7 | 0.9(6) |  | C11 | C12 | C13 | C8 | 1.1(6) |
| N1 | C11 | C12 | C13 | 178.3(4) |  | C13 | C8 | C9 | C10 | 0.2(6) |
| N1 | C14 | C15 | N2 | 0.1(5) |  | C14 | N1 | C11 | C10 | -38.6(6) |
| C1 | C2 | C3 | C4 | 173.0(4) |  | C14 | N1 | C11 | C12 | 142.4(4) |
| C1 | C2 | C7 | C6 | -173.6(4) |  | C14 | N1 | C16 | N2 | -0.6(5) |
| C2 | C3 | C4 | C5 | 0.3(6) |  | C15 | N2 | C16 | N1 | 0.7(5) |
| C3 | C2 | C7 | C6 | 0.7(6) |  | C16 | N1 | C11 | C10 | 141.9(4) |
| C3 | C4 | C5 | C6 | 1.3(6) |  | C16 | N1 | C11 | C12 | -37.1(6) |
| C3 | C4 | C5 | C8 | -178.2(4) |  | C16 | N1 | C14 | C15 | 0.3(5) |
| C4 | C5 | C6 | C7 | -1.8(6) |  | C16 | N2 | C15 | C14 | -0.5(5) |
| C4 | C5 | C8 | C9 | 29.7(6) |  |  |  |  |  |  |

13/2-X,+Y,1/2-Z

Table 8 Hydrogen Atom Coordinates (Å×104) and Isotropic Displacement Parameters (Å2×103) for shI\_4151\_BeDa.

| Atom | *x* | *y* | *z* | U(eq) |
| --- | --- | --- | --- | --- |
| H2 | 1731.81 | -4478 | 5945.04 | 24 |
| H3 | 5354.43 | 3208.19 | 1678.23 | 18 |
| H4 | 4593.13 | 1294.83 | 2110.69 | 18 |
| H6 | 4133.9 | 3162.05 | 4492.27 | 19 |
| H7 | 4925.18 | 5061.3 | 4068.18 | 21 |
| H9 | 4796.24 | -522.9 | 3103.37 | 23 |
| H10 | 4098.14 | -2467.9 | 3581 | 21 |
| H12 | 1994.02 | -350.75 | 4792.64 | 22 |
| H13 | 2724.18 | 1576.54 | 4338.95 | 21 |
| H14 | 2352.6 | -4162.2 | 3401.52 | 29 |
| H15 | 1659.63 | -5696.21 | 4449.79 | 29 |
| H16 | 2449.22 | -2297.99 | 5926.96 | 21 |

shI\_4151\_BeDa


# shI\_4151\_BeDa

Table 1 Crystal data and structure refinement for shI\_4151\_BeDa.

| Identification code | shI\_4151\_BeDa |
| Empirical formula | C32H24HoN7O13 |
| Formula weight | 879.51 |
| Temperature/K | 180.00(14) |
| Crystal system | monoclinic |
| Space group | P2/n |
| a/Å | 11.6138(3) |
| b/Å | 10.0845(2) |
| c/Å | 14.0087(4) |
| α/° | 90 |
| β/° | 109.726(3) |
| γ/° | 90 |
| Volume/Å3 | 1544.41(7) |
| Z | 2 |
| ρcalcg/cm3 | 1.891 |
| μ/mm‑1 | 2.647 |
| F(000) | 872.0 |
| Crystal size/mm3 | 0.25 × 0.05 × 0.05 |
| Radiation | Mo Kα (λ = 0.71073) |
| 2Θ range for data collection/° | 3.956 to 50.044 |
| Index ranges | -13 ≤ h ≤ 13, -12 ≤ k ≤ 12, -16 ≤ l ≤ 16 |
| Reflections collected | 22162 |
| Independent reflections | 2727 [Rint = 0.0557, Rsigma = 0.0343] |
| Data/restraints/parameters | 2727/0/241 |
| Goodness-of-fit on F2 | 1.040 |
| Final R indexes [I>=2σ (I)] | R1 = 0.0304, wR2 = 0.0737 |
| Final R indexes [all data] | R1 = 0.0359, wR2 = 0.0764 |
| Largest diff. peak/hole / e Å-3 | 0.86/-0.66 |
