# Supplementary material for: Novel Lanthanide (III) Complexes Derived from an Imidazole–Biphenyl–Carboxylate Ligand: Synthesis, Structure and Luminescence Properties
Source: Molecules. 2021 Nov 17;26(22):6942. doi: 10.3390/molecules26226942 (PMC8625298; doi:10.3390/molecules26226942)
Supplement: Supplementary file 1 [file molecules-26-06942-s001.zip › molecule-1430069 - Supplimentary material_updated 25.11.2021.pdf]

Electronic supplementary material for

Novel Lanthanide (III) Complexes Derived from an Imidazole–Biphenyl–Carboxylate Ligand: Synthesis, Structure and Luminescence Properties

Monica-Cornelia Sardaru [1], Narcisa Laura Marangoci [1], Sergiu Shova [2], Dana Bejan[1,*]

1 Centre of Advanced Research in Bionanoconjugates and Biopolymers, “Petru Poni” Institute of Macromolecular Chemistry, Romanian Academy, Gr. Ghica Voda Alley, Iasi 700487, Romania; sardaru.monica@icmpp.ro (M.C.S.); nmarangoci@icmpp.ro (N.L.M.)

2 Department of Inorganic Polymers, “Petru Poni” Institute of Macromolecular Chemistry, Romanian Academy, Gr. Ghica Voda Alley, Iasi 700487, Romania; shova@icmpp.ro

*bejan.dana@icmp.ro (D.B.)

Content

1. Powder X-ray Diffraction P2–P3
2. Infrared spectroscopy (ATR) P4–P7
3. Thermal (TG/DTG) Analysis P8–P9
4. Luminiscence properties P10

1. Powder X-ray Diffraction Strategy A

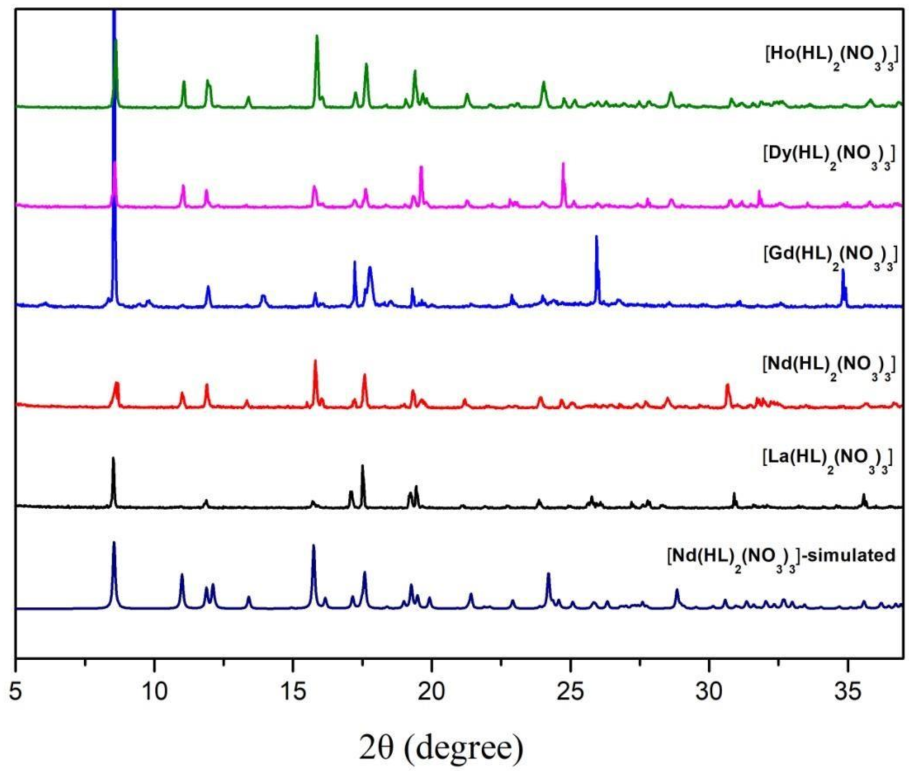

Figure S1. PXRD patterns of the isostructural compounds versus simulated powder PXRD pattern generated from a single crystal cif file of **[Nd(HL)$_2$(NO$_3$)$_3$] (3)** compound (in EtOH/H_2O as presented in Scheme 1, Strategy A)

Strategy B

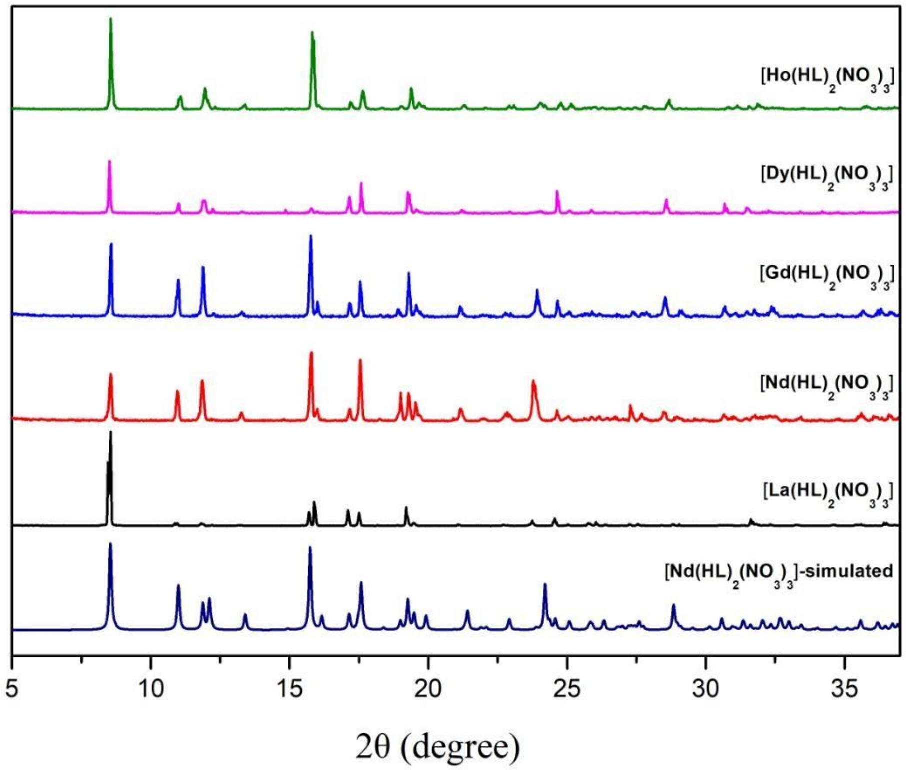

Figure S2. PXRD patterns of the isostructural compounds versus simulated powder PXRD pattern generated from a single crystal cif file of **[Nd(HL)$_2$(NO$_3$)$_3$] (3)** compound (in CH_3CN/H_2O as presented in Scheme 1, Strategy B)

2. Infrared spectroscopy (ATR)

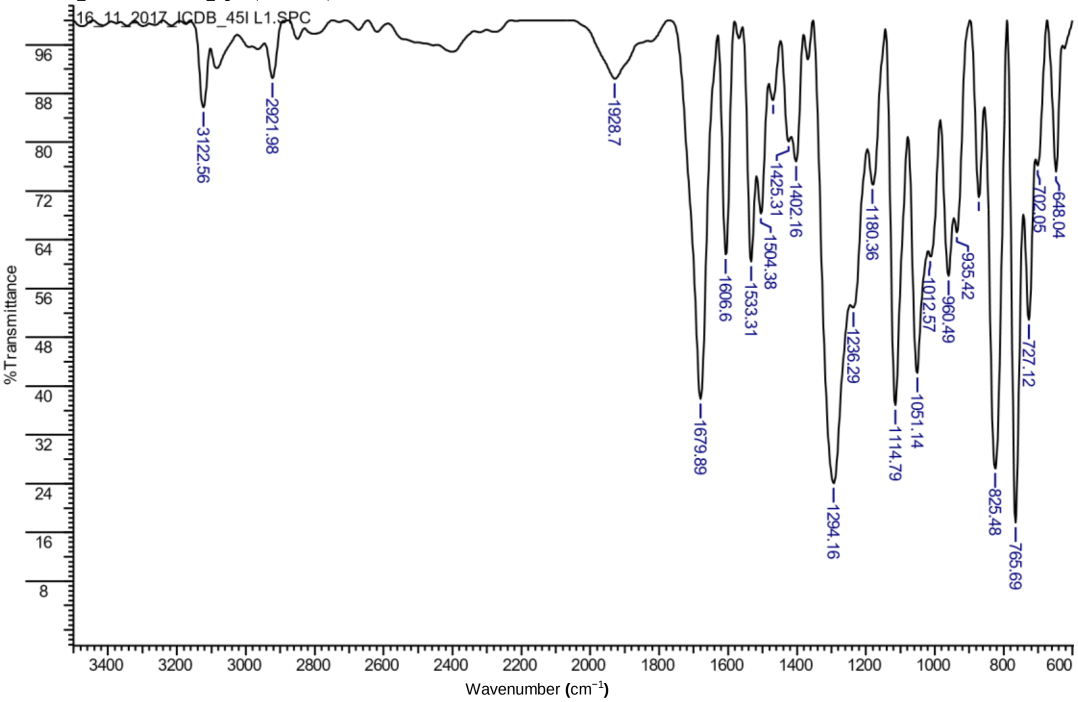

Figure S3. IR-spectrum of 4'-(1*H*-imidazol-1-yl)biphenyl-4-carboxylic acid (**HL**).

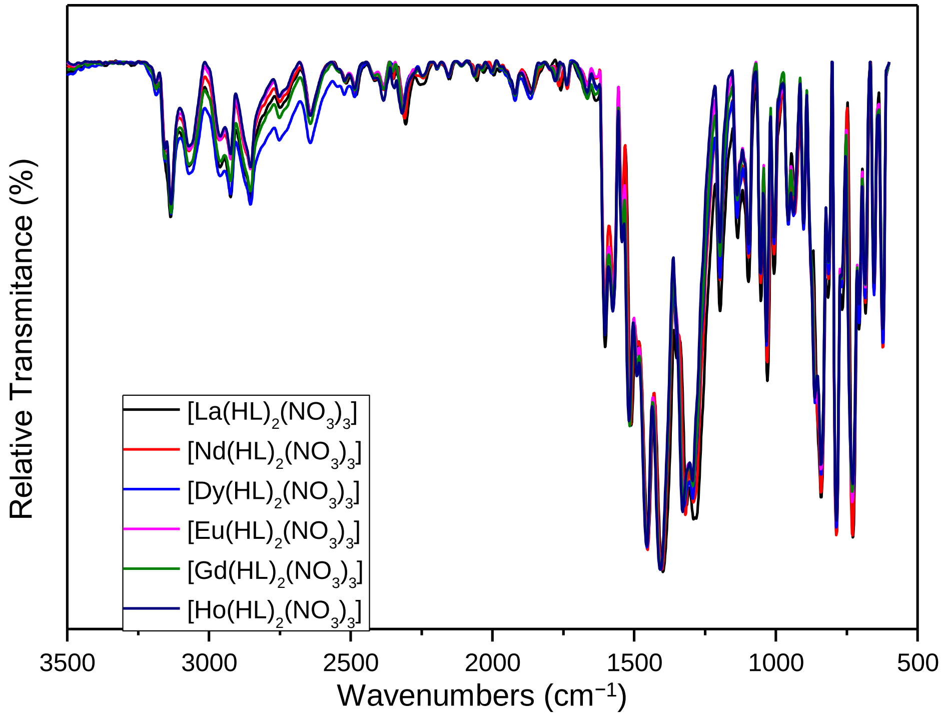

Figure S4. IR-spectra of **[Ln(HL)$_2$(NO$_3$)$_3$] (Ln = La, Nd, Dy, Eu, Gd, Ho)**

[La(HL)$_2$(NO$_3$)$_3$] (1)

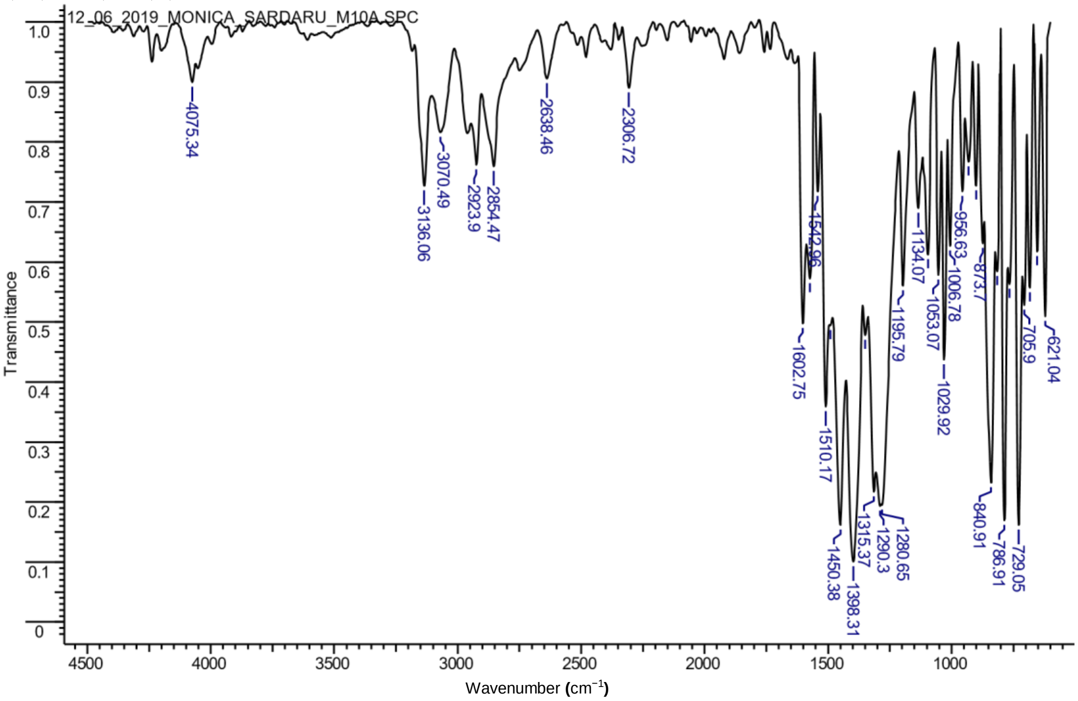

Figure S5. IR-spectrum of **[La(HL)$_2$(NO$_3$)$_3$]**

[Nd(HL)$_2$(NO$_3$)$_3$] (3)

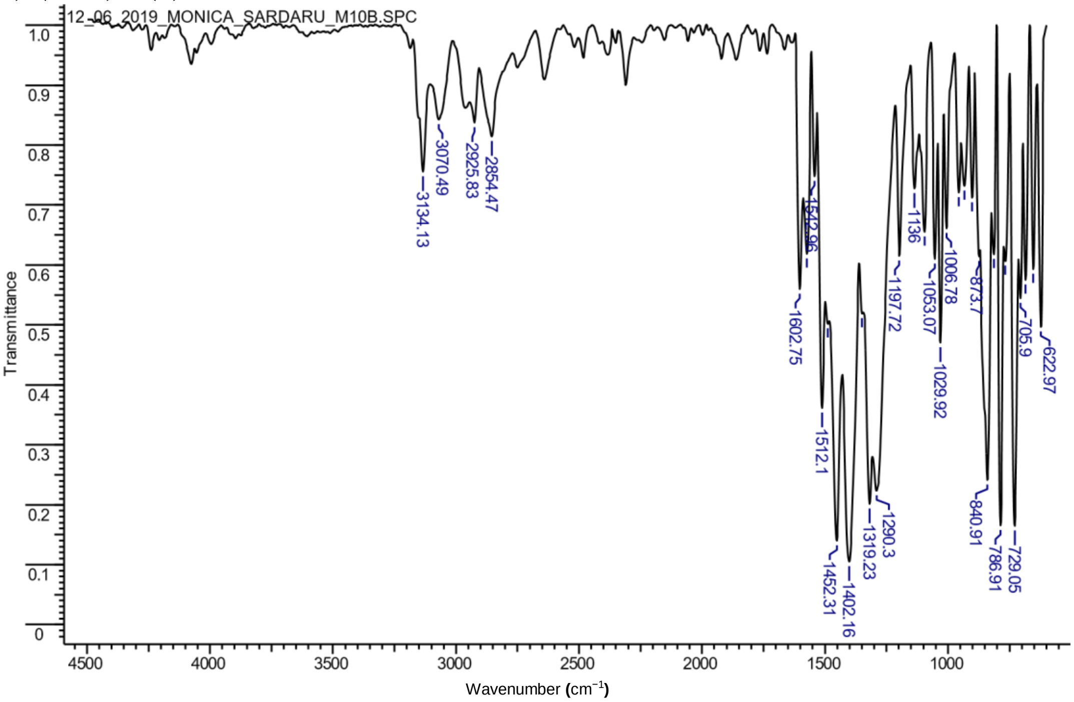

Figure S6. IR-spectrum of **[Nd(HL)$_2$(NO$_3$)$_3$]   [Gd(HL)$_2$(NO$_3$)$_3$] (5)**

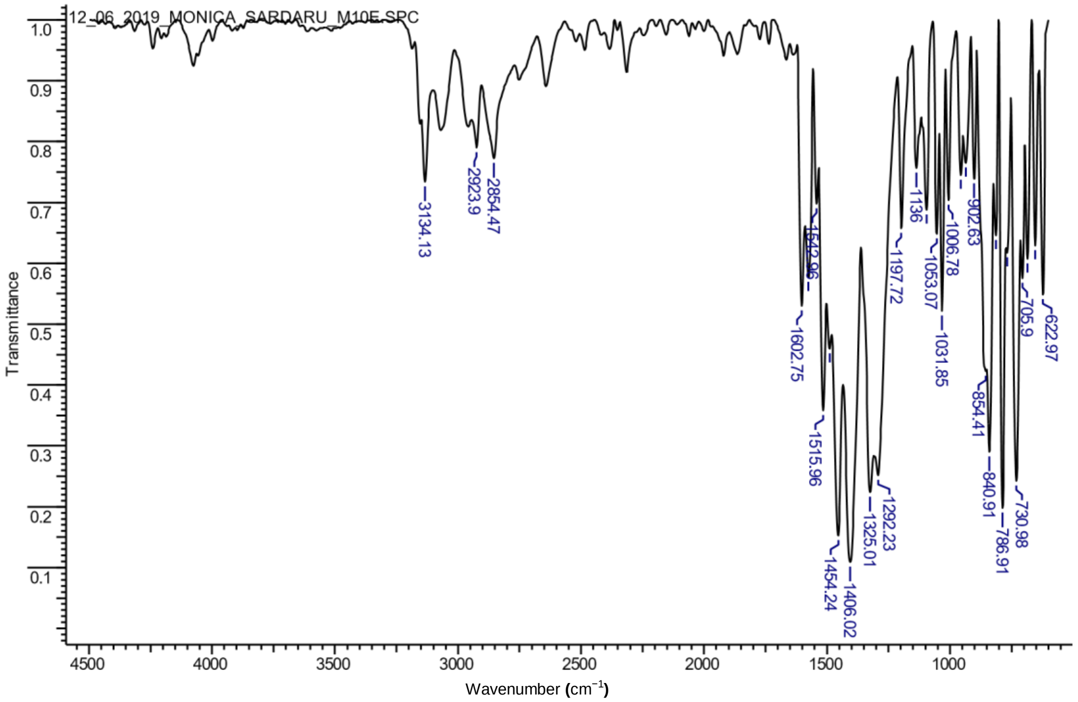

Figure S7. IR-spectrum of **[Gd(HL)$_2$(NO$_3$)$_3$]**

[Dy(HL)$_2$(NO$_3$)$_3$] (7)

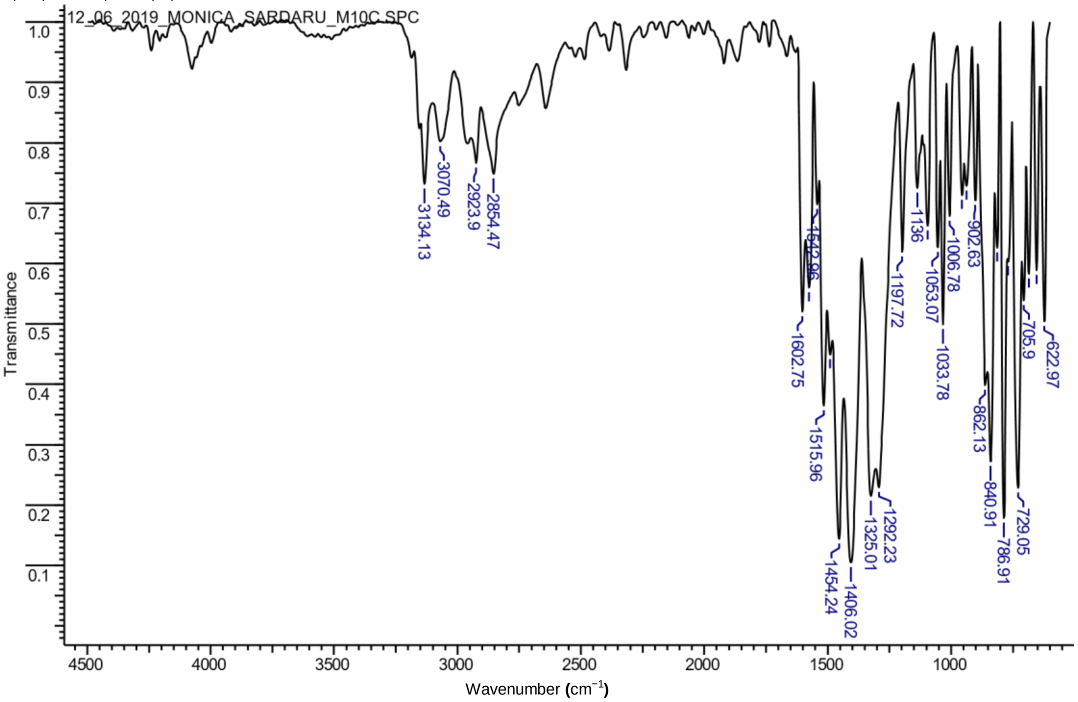

Figure S8. IR-spectrum of **[Dy(HL)$_2$(NO$_3$)$_3$] [Ho(HL)$_2$(NO$_3$)$_3$] (8)**

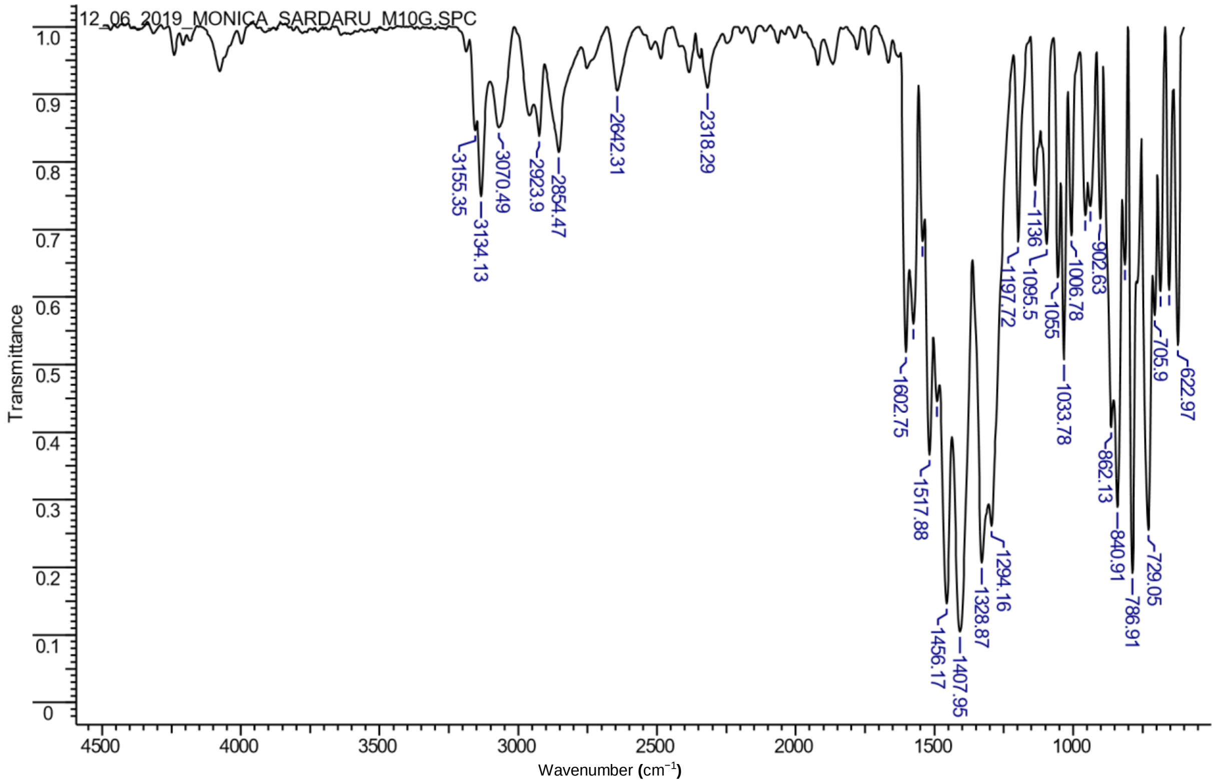

Figure S9. IR-spectrum of **[Ho(HL)$_2$(NO$_3$)$_3$]**

3. Thermal (TG/DTG) Analysis [Nd(HL)$_2$(NO$_3$)$_3$] (3)

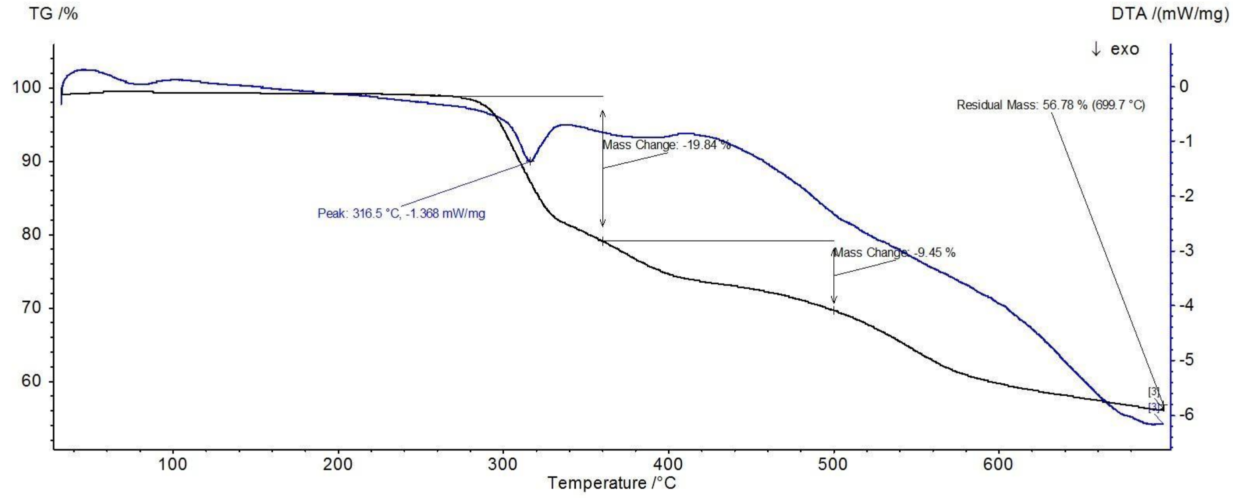

Figure S10. Thermogravimetric analysis of compound **[Nd(HL)$_2$(NO$_3$)$_3$] (3)**

[Gd(HL)$_2$(NO$_3$)$_3$] (5)

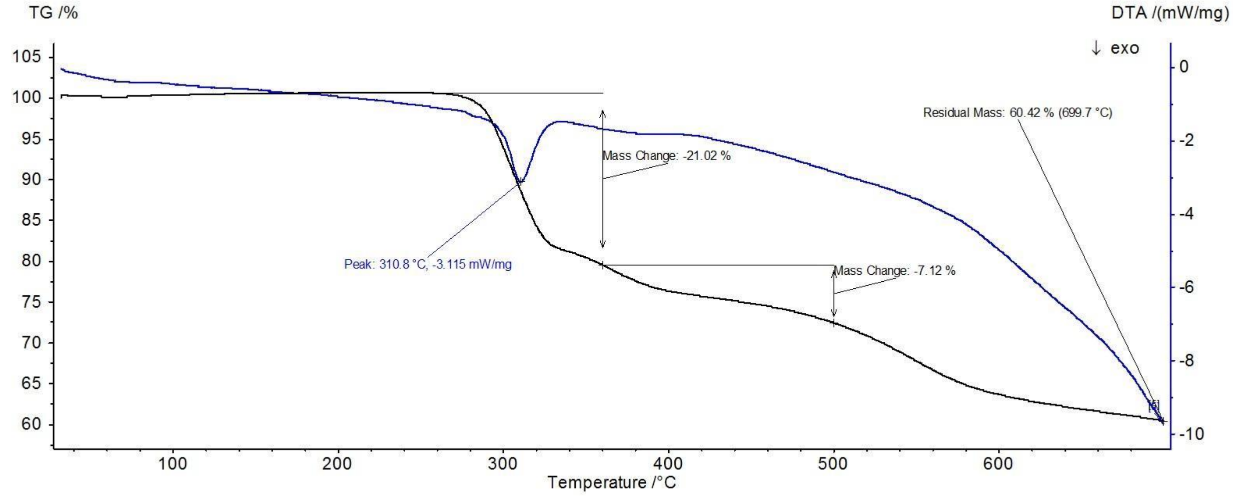

Figure S11**.** Thermogravimetric analysis of compound **[Gd(HL)$_2$(NO$_3$)$_3$] (5)**.

[Dy(HL)$_2$(NO$_3$)$_3$] (7)

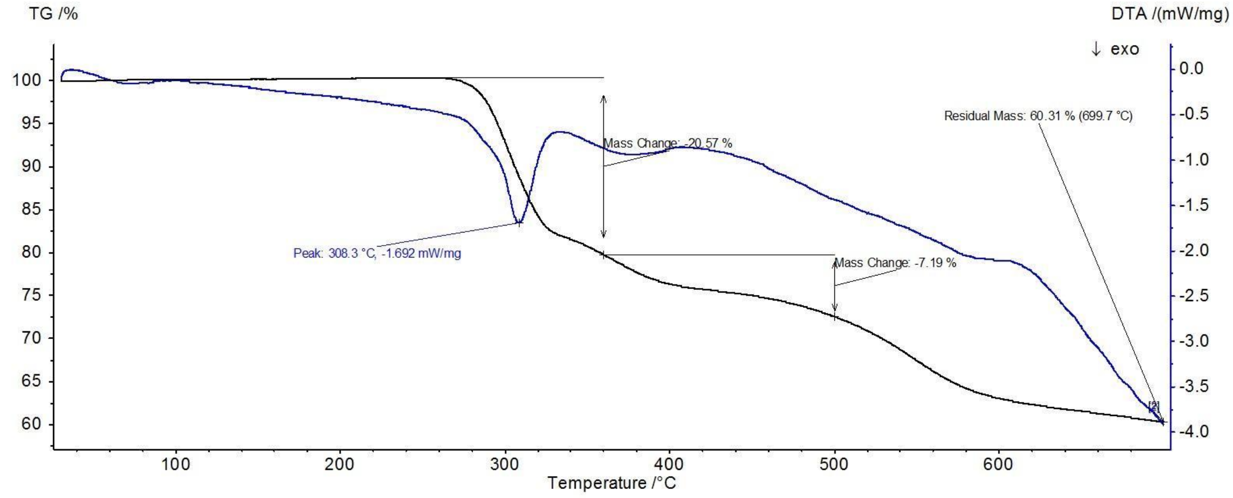

Figure S12. Thermogravimetric analysis of compound **[Dy(HL)$_2$(NO$_3$)$_3$] (7)**.

[Ho(HL)$_2$(NO$_3$)$_3$] (8)

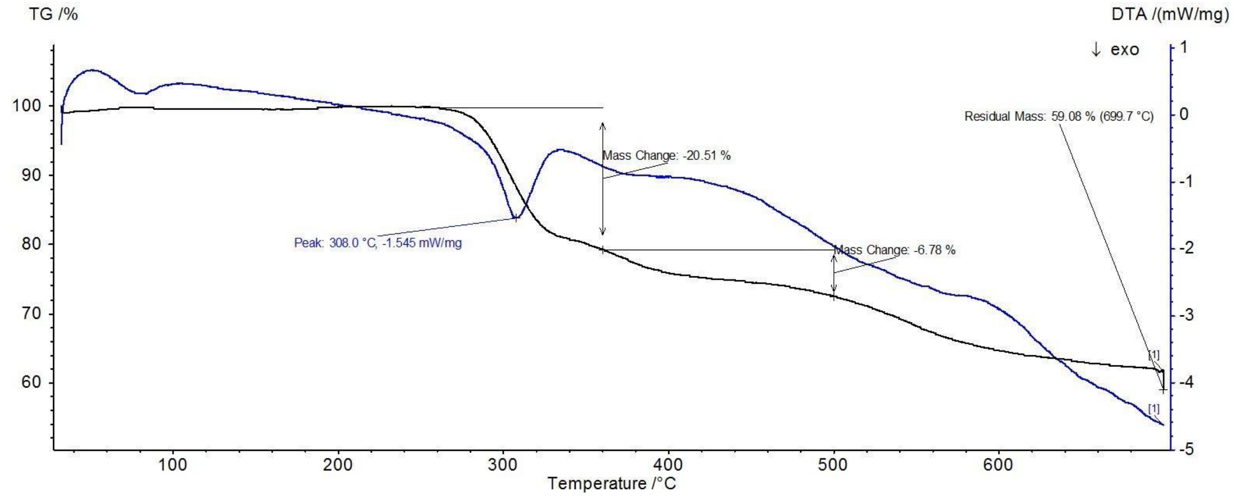

Figure S13. Thermogravimetric analysis of compound **[Ho(HL)$_2$(NO$_3$)$_3$] (8)**.

4. Luminiscence properties

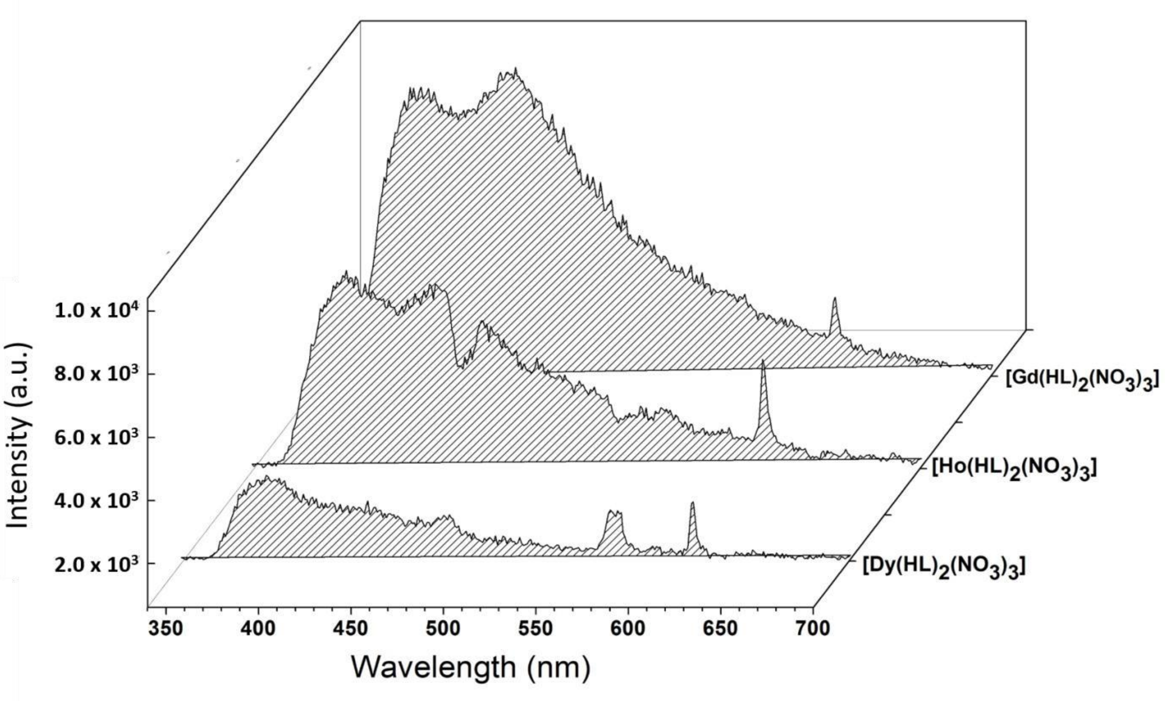

Figure S14. Emission spectra of three complexes (Gd, Ho, Dy) obtained under excitation at 315 nm.

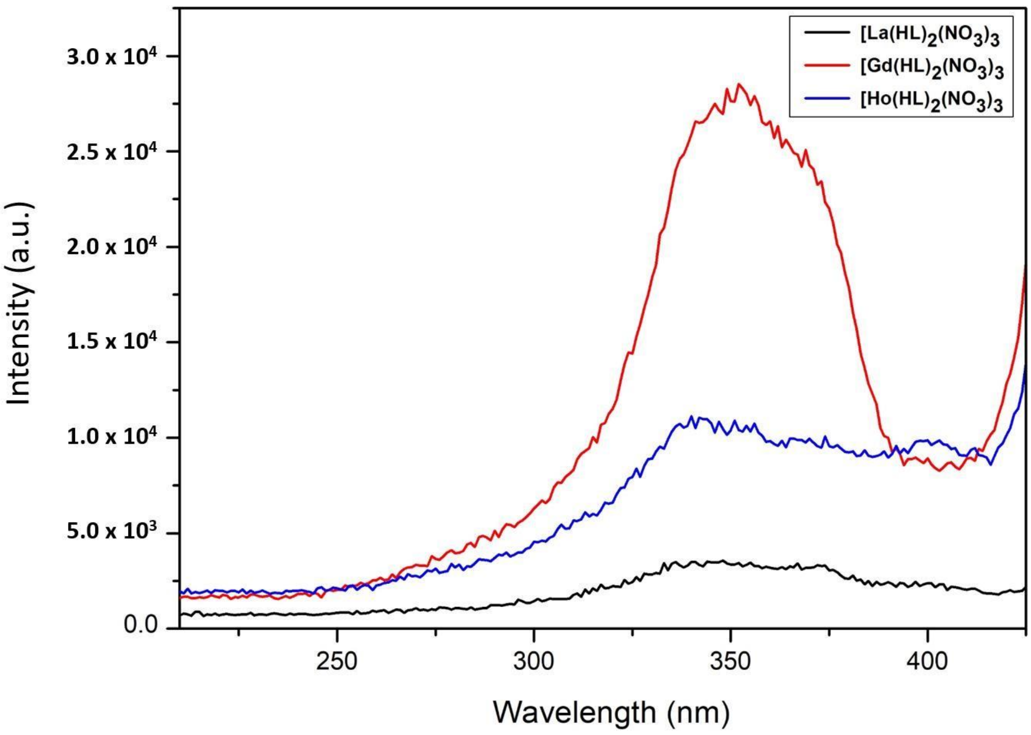

Figure S15. Excitation spectra of three complexes (Eu, Gd, Ho) obtained at 440 nm (2D chart).
